# A Miocene impact ejecta layer in the pelagic Pacific Ocean

**DOI:** 10.1038/s41598-019-52709-1

**Published:** 2019-11-20

**Authors:** Tatsuo Nozaki, Junichiro Ohta, Takaaki Noguchi, Honami Sato, Akira Ishikawa, Yutaro Takaya, Jun-Ichi Kimura, Qing Chang, Kazuhiko Shimada, Jun-ichiro Ishibashi, Kazutaka Yasukawa, Katsunori Kimoto, Koichi Iijima, Yasuhiro Kato

**Affiliations:** 1 Submarine Resources Research Center, Research Institute for Marine Resources Utilization, Japan Agency for Marine-Earth Science and Technology (JAMSTEC), 2-15 Natsushima-cho, Yokosuka, Kanagawa 237-0061 Japan; 2 Frontier Research Center for Energy and Resources (FRCER), School of Engineering, The University of Tokyo, 7-3-1 Hongo, Bunkyo-ku, Tokyo 113-8656 Japan; 3 Department of Planetology, Kobe University, 1-1 Rokkodai-cho, Nada-ku, Kobe, Hyogo 657-8501 Japan; 4 Ocean Resources Research Center for Next Generation, Chiba Institute of Technology, 2-17-1 Tsudanuma, Narashino, Chiba 275-0016 Japan; 5 Department of Systems Innovation, School of Engineering, The University of Tokyo, 7-3-1 Hongo, Bunkyo-ku, Tokyo 113-8656 Japan; 6 Volcanoes and Earth’s Interior Research Center, Research Institute for Marine Geodynamics, Japan Agency for Marine-Earth Science and Technology (JAMSTEC), 2-15 Natsushima-cho, Yokosuka, Kanagawa 237-0061 Japan; 7 Division for Experimental Natural Science, Faculty of Arts and Science, Kyushu University, 744 Motooka, Nishi-ku, Fukuoka 819-0395 Japan; 8 Department of Earth and Planetary Sciences, Tokyo Institute of Technology, 2-12-1 Ookayama, Meguro-ku, Tokyo 152-8550 Japan; 9 Department of Resources and Environmental Engineering, School of Creative Science and Engineering, Waseda University, 3-4-1 Okubo, Shinjuku-ku, Tokyo 169-8555 Japan; 10 Department of Earth and Planetary Sciences, Faculty of Science, Kyushu University, 744 Motooka, Nishi-ku, Fukuoka 819-0395 Japan; 11 Earth Surface System Research Center, Research Institute for Global Change, Japan Agency for Marine-Earth Science and Technology (JAMSTEC), 2-15 Natsushima-cho, Yokosuka, Kanagawa 237-0061 Japan

**Keywords:** Geochemistry, Stratigraphy, Mineralogy, Palaeontology, Sedimentology

## Abstract

Meteorite impacts have caused catastrophic perturbations to the global environment and mass extinctions throughout the Earth’s history. Here, we present petrographic and geochemical evidence of a possible impact ejecta layer, dating from about 11 Ma, in deep-sea clayey sediment in the Northwest Pacific. This clay layer has high platinum group element (PGE) concentrations and features a conspicuous negative Os isotope anomaly (^187^Os/^188^Os as low as ~0.2), indicating an influx of extraterrestrial material. It also contains abundant spherules that include pseudomorphs suggestive of porphyritic olivine as well as spinel grains with euhedral, dendritic and spherical forms and NiO contents as great as 23.3 wt%, consistent with impact ejecta. Osmium isotope stratigraphy suggests a most plausible depositional age of ~11 Ma (Miocene) for this layer, as determined by fitting with the seawater evolution curve. No large impact crater of this age is known on land, even within the relatively large uncertainty range of the relative Os age. Thus, we suggest that an unrecognised impact event in the middle or late Miocene produced the impact ejecta layer of the Northwest Pacific.

## Introduction

Extraterrestrial objects larger than 1 km in diameter collide with Earth roughly once every million years, as estimated from astrophysical models or craters preserved on land^1,2^. However, only 28 distal impact ejecta or spherule layers have been confirmed from the Earth’s surface^3^ from the presence of shocked quartz, Ni-rich spinel, anomalous PGE enrichments or negative excursions of Os isotope composition. Although oceanic impact events must be frequent from a geological perspective, only one, the 2.51 ± 0.07 Ma Eltanin impact in the Southern Ocean, has been confirmed by mineralogical, geochemical, lithostratigraphic and geophysical studies^4–7^. Deep-sea sediments, however, should preserve ejecta from both oceanic and on-land impacts as their slow deposition rate minimises dilution of impactor debris, especially in settings deeper than the carbonate compensation depth. We here present petrographic and geochemical evidence of a possible impact ejecta layer produced by a middle or late Miocene impact event at around 11 Ma.

The ejecta layer was observed within a layer of pelagic clay in the Northwest Pacific Ocean that was cored in 2014 during the cruise MR14-E02 of *R/V Mirai*^8,9^ (Supplementary Figs S1 and S2; see Materials and methods). A conspicuous negative Os isotope excursion and an abnormal enrichment of Os were found in a ~40-cm-thick layer of clay ~350 cm below the seafloor (cmbsf) in piston core 11 (PC11). Below, we report bulk major- and trace-element compositions, Re-Os isotope data and PGE concentrations in the piston core samples, as well as detailed petrographic observations of this layer, that collectively indicate the presence of extraterrestrial material.

## Results

### Re and Os geochemistry

**Figure 1:**
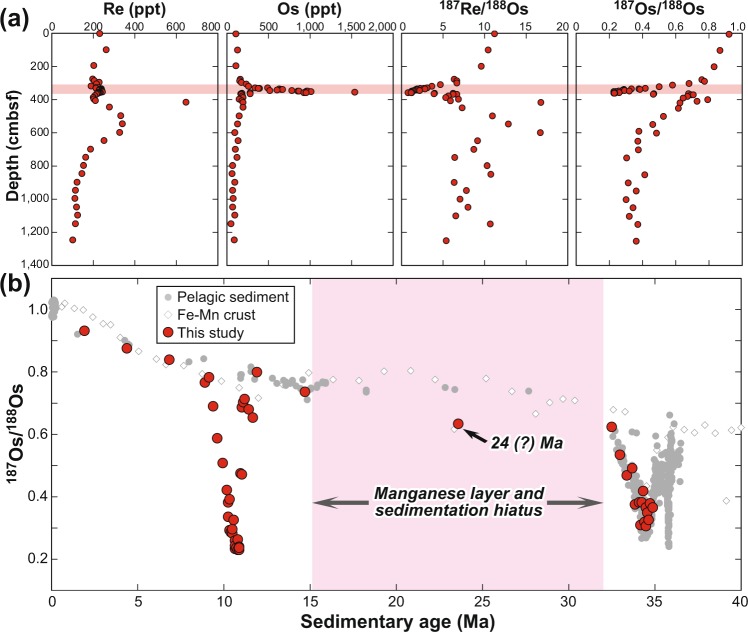
Re-Os geochemistry of piston core samples. (**a**) Re and Os concentrations and isotope ratios. (**b**) Depositional age determined by Os isotope stratigraphy. Os isotope data for pelagic sediment and Fe-Mn crusts are from refs^11,18,48–57^. Measurements were made by the sparging method plus MC-ICP-MS and ICP-QMS (see Materials and methods). Coloured bars indicate stratigraphic and age interval characterised by high Mn concentrations and depositional hiatus.

The PC11 samples contain 101–649 ppt Re and 53.2–1,536 ppt Os (Fig. 1a and Supplementary Table S1), ranges that exceed those of the average upper continental crust (Re = 198 ppt, Os = 31 ppt)^10^ by as much as 3.3 times for Re and 50 times for Os. The range of isotope ratios in these samples is 0.781–16.9 for ^187^Re/^188^Os and 0.2274–0.927 for ^187^Os/^188^Os.

Because the site of core PC11 is below the carbonate compensation depth, the core contains almost no age-diagnostic calcareous microfossils. Instead, we used Os isotope stratigraphy to constrain the depositional age of the core samples. Seafloor mud preserves both the Os isotopic composition of seawater and its low initial Re/Os ratio at the time of deposition^11^. The decay of ^187^Re to ^187^Os since deposition can change ^187^Os/^188^Os ratios only by as much as 2.86%, even assuming an initial depositional age of 50 Ma (Supplementary Table S1). Depositional ages are determined by comparing the Os isotope compositions of core samples to the secular variation curve of global seawater^11–14^. Because the marine Os isotope curve changed so little between 20 and 10 Ma, the precision of age estimates is poor for that time interval. However, we estimated the most plausible depositional age of the Os isotope excursion to be ~11 Ma on the basis of the pre- and post-excursion ^187^Os/^188^Os values of 0.78–0.80 as well as lithological features and thickness of strata (Fig. 1b) (see Materials and methods). We cannot rule out the possibility that the depositional age of the layer with the Os isotope excursion is as much as several million years younger than the most plausible age (~11 Ma), but a depositional age older than ~13 Ma is unlikely based on the post-excursion ^187^Os/^188^Os ratio of 0.796 ± 0.005, which is significantly more radiogenic (beyond analytical error) than that of pelagic sediment dated at 13–16 Ma (Fig. 1b).

Since the first application of the Os isotope system to impact events^15^, many studies have used Re-Os isotope systematics for better understanding of impactites and the effects of impact events on the Earth as well as for estimates of the impactor size^16–20^. Arguments based on Os geochemistry suggest that Os concentrations exceeding 1,000 ppt and conspicuous negative ^187^Os/^188^Os excursions as low as ~0.23 can only be produced by large influxes of unradiogenic Os from ultramafic rocks or meteoritic materials^17,20,21^. The pelagic clay with the negative Os isotope excursion is in the depth interval 308.9–360.9 cmbsf of PC11 and consists predominantly of clay minerals (>80%), with trace amounts of zeolite and biogenic calcium phosphate (BCP) and rare mafic minerals and glass derived from volcanic materials (<1%). Because typical ultramafic rocks (e.g., peridotite reference materials UB-N and JP-1^22,23^) contain ~4,000 ppt Os and have ^187^Os/^188^Os ratios of ~0.125, it would require unrealistic amounts of ultramafic rock to account for the observed Os isotope excursion and Os enrichment. Moreover, there is no major- and trace-element evidence of ultramafic material at the level of the Os isotope excursion except for a small positive Cr peak (Supplementary Fig. S3 and Table S2). Therefore, we attribute the Os isotope excursion and Os enrichment to an influx of meteoritic material.

### Platinum group element geochemistry

**Figure 2:**
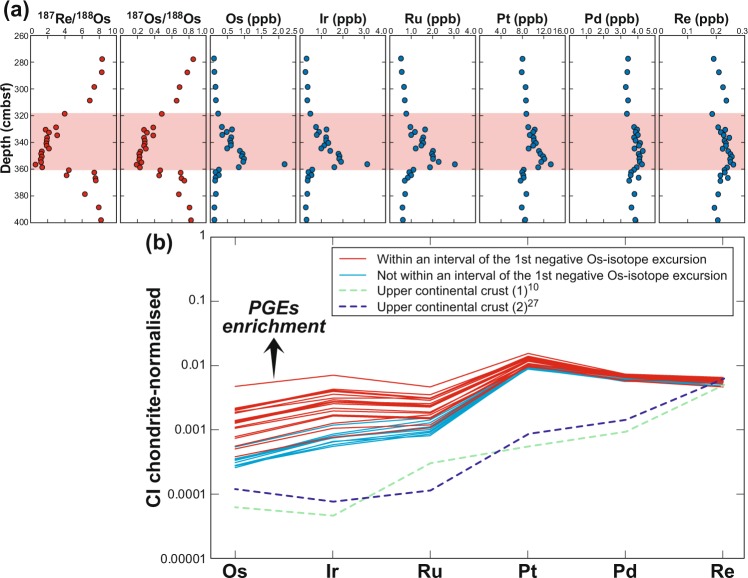
PGE concentrations and CI chondrite-normalised PGE patterns of core samples. (**a**) Depth profiles of PGE and Re concentrations and Re-Os isotope ratios. Measurements were made by the solvent extraction method plus TIMS and DF-ICP-MS (see Materials and methods). (**b**) Chondrite-normalised PGE patterns. Red and blue curves denote samples within and outside the shaded area in (**a**), respectively. PGE and Re concentrations of upper continental crust and CI chondrites are from refs^10,27,59^.

We determined the PGE concentrations of 29 samples from the 278.1–398.3 cmbsf interval of PC11 (Supplementary Table S3), including 21 samples taken at 2 cm intervals continuously from 328.9 to 368.9 cmbsf (Fig. 2a). We conducted additional Re-Os isotope analyses to cross-check the data quality using different spike solutions and analytical methods. PGE concentrations were well above background levels, reaching 2.19 ppb Os, 3.16 ppb Ir, 3.05 ppb Ru, 13.4 ppb Pt, 4.28 ppb Pd and 0.261 ppb Re (Supplementary Table S3). The maximum Ir concentration was comparable to that in ejecta from the Late Triassic impact event^24,25^ and only slightly lower than that of ejecta at the Cretaceous-Paleogene boundary^26^. The greatest PGE enrichments coincided with the peak negative Os isotope excursion (^187^Os/^188^Os = 0.189 in sample PC11-4_62-64), excepting only Pd, which was at its second-highest concentration among the 29 samples.

The 29 samples from 278.1–398.3 cmbsf showed a pattern of enrichment in platinum subgroup of PGE (PPGE; Pd, Pt and Rh) (Fig. 2b), typical in pelagic sediment and the upper continental crust^9,27^. The 12 samples outside the Os isotope excursion had highly PPGE-enriched patterns typical of pelagic clay in this area (blue lines in Fig. 2b). The 17 samples within the Os isotope excursion had greater and more consistent PGE enrichments, evident as flatter PGE patterns (red lines in Fig. 2b). The high concentrations of Os and Ir in these samples could be explained by a mixture of 99.5% pelagic clay and 0.5% CI-chondrite, but such a mixture does not account for the observed iridium subgroup of PGE (IPGE; Ir, Os and Ru) enrichment. However, a mixture of pelagic clay and a PPGE-rich terrestrial impact melt, itself a mixture of a chondritic impactor and oceanic crust plus pelagic clay, could readily account for the observed PGE patterns.

### Microscopic observation and analysis

Based on the results of Re-Os isotope and PGE concentration analyses, we conducted a microscopic X-ray computed tomography (micro-XCT) analysis to observe constituent minerals of the clay layer in a 0.211 g wet subsample (0.116 g dry weight) of sample PC11-4_62-64, which had the highest PGE concentrations and most unradiogenic Os isotope composition. The micro-XCT examination revealed the presence of spherule grains, BCP and Fe-Mn oxides (Supplementary Fig. S4). Cross-section images of the largest spherule grain showed an inner structure consisting of tabular hexagonal shapes surrounded by higher radiodensity grains several to a dozen micrometres in size (Supplementary Fig. S4b,c). Using radiodensity and morphology to distinguish BCP and Fe-Mn oxides from the spherules, we counted 610 spherules in this subsample. Their longest dimension was less than 50 μm in 90% of the spherules, but two spherules were longer than 350 μm (Supplementary Fig. S5).

**Figure 3:**
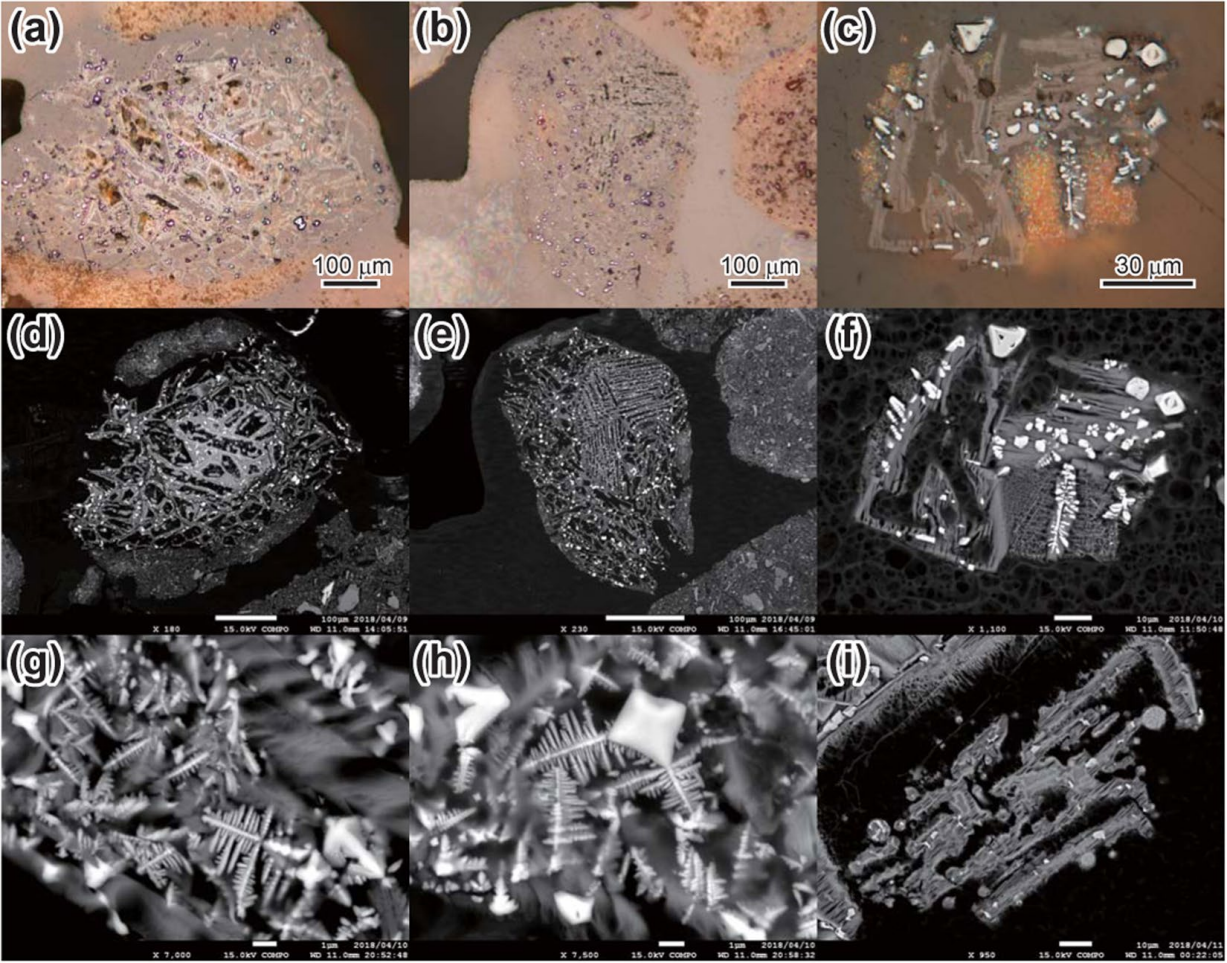
Reflected-light photomicrographs (**a–c**) and back-scatter electron (BSE) images of spinel grains in spherules (**d–i)**. Spinel-rich spherules are mantled by pelagic sediment. (**a**,**d**) Spinel grains are concentrated at the outer rim of olivine pseudomorphs replaced by clay minerals. (**b**,**e**) Spherules often exhibit a barred olivine-like texture. (**c**,**f–i**) Morphologies of spinel grains in the spherules are classified into euhedral, dendritic, intermediate, and spherical. (**g**,**h**) Dendritic spinel formed by partial melting and quenching of euhedral spinel grains. (**i**) Spherical spinel with high NiO content.

We extracted the coarse fractions (>62 μm) of the five samples between 349.9 and 359.9 cmbsf, which had the highest PGE concentrations and most unradiogenic Os isotope values, and mounted them in acrylic resin for petrographic examination (Figs 3, S6). Each of these extracts contained dozens of reddish-brown spherules as large as 300 μm in diameter (Fig. 3a–c). The coarse fraction from sample PC11-4_62-64, with the most unradiogenic Os, contained the most spherules. The spherules were covered by a thin layer of clay-size particles consisting of quartz, zeolite, magnetite, Fe-Mn oxides, BCP, illite and other trace minerals. Their interiors consisted mainly of pseudomorphs suggestive of porphyritic olivine, completely replaced by clay minerals (Figs 3d, S6a,d). The rims of the pseudomorphs contained many spinel grains less than 10 μm in diameter (Fig. 3d–f), displaying euhedral (Figs 3f, S6e) to dendritic shapes (Figs 3f–h, S6b,c,f), consistent with the micro-XCT cross-section images (Supplementary Fig. S4b,c). Dendritic spinel commonly protruded from euhedral spinel grains (Figs 3g,h, S6g–k).

Spinel grains ranged widely in composition from hercynite (FeAl_2_O_4_) to magnetite (Fe_3_O_4_) and chromite (FeCr_2_O_4_). Major-element concentrations determined by electron probe microanaliser (EPMA) were 0.14–16.52 wt% Al_2_O_3_, 0.13–8.61 wt% MgO, 34.42–92.41 wt% FeO* (total iron as FeO) and 0.09–41.80 wt% Cr_2_O_3_ (Supplementary Fig. S7 and Table S4). Euhedral grains had cores with higher Cr_2_O_3_ contents than dendritic grains, which had compositions similar to magnetite (Supplementary Figs S7–S9 and Table S4). Assuming spinel stoichiometry^28^ for seven constituent cations (Mg, Al, Fe, Cr, Ni, Mn and Ti), most of the dendritic spinel grains had a magnetite component (Fe_3_O_4_) greater than 80%, whereas most of the euhedral spinel grains exceeded the dendritic grains in the chromite component (FeCr_2_O_4_) (Supplementary Fig. S8 and Table S5). The NiO contents ranged from 0.04 to 4.78 wt% in both euhedral and dendritic grains, whereas three small spherical spinel grains on the rims of olivine pseudomorphs (Figs 3I, S6l) had NiO contents ranging from 3.30 to 23.29 wt%.

**Figure 4:**
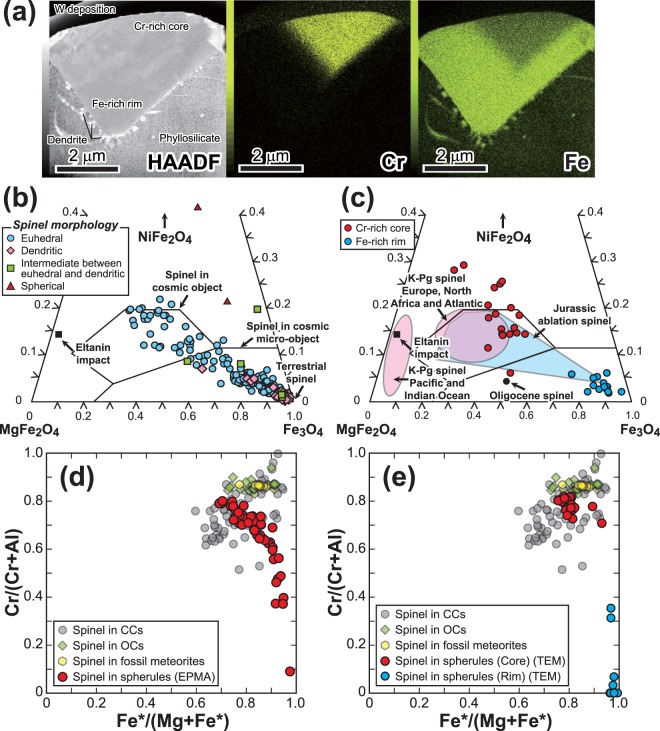
High-angle annular dark field (HAADF)–scanning transmission electron microscopy image with TEM-EDS elemental mapping images and compositional diagrams of spinel grains. (**a**) HAADF image with TEM-EDS elemental mapping images of Cr and Fe in a chromite component–rich euhedral spinel crystal associated with dendritic spinels. (**b**,**c**) NiFe_2_O_4_–MgFe_2_O_4_–Fe_3_O_4_ (trevorite–magnesioferrite–magnetite) ternary diagrams of spinel grains obtained by (**b**) EPMA and (**c**) TEM analyses. Compositional fields of spinels are from refs^29,60^ and references therein. (**d,e**) Fe*/(Mg + Fe*) vs. Cr/(Al + Cr) diagrams of spinel grains obtained by (**d**) EPMA and (**e**) TEM analyses. Spinel compositions in carbonaceous chondrites (CCs), ordinary chondrites (OCs) and Ordovician fossil meteorites are from refs^31–33^.

Thin sections of two Cr-rich euhedral spinel grains were prepared by a focused ion beam scanning electron microscope (FIB-SEM) and observed by transmission electron microscope (TEM). Elemental mapping analyses using TEM energy-dispersive X-ray spectrometry showed a clear boundary in one of these grains between a Cr-rich core and an Fe-rich rim in addition to Fe-rich dendritic growths on the rim (Figs 4a, S10). We interpret these textural and chemical features as the result of partial melting of euhedral spinel originating from an impactor and the formation of dendritic growths during rapid quenching, consistent with textures formed by meteorite impacts. In this interpretation, the Cr-rich core was a relict grain from the impact melt and the Fe-rich rim and dendrites were produced from the impact melt by a combined quenching and oxidation process. Even the cores of the Cr-rich spinels contained some Fe^3+^ and Ni, consistent with their formation, and thus oxidation, in the lower atmosphere, as spinels in chondritic meteorites usually do not contain detectable NiO except in CK and R chondrites^29^. On NiFe_2_O_4_–MgFe_2_O_4_–Fe_3_O_4_ (trevolite–magnesioferrite–magnetite) ternary diagrams, spinel grains from the spherules in core PC11 exhibit a wide range in composition from cosmic to terrestrial (Fig. 4b,c). Both EPMA and TEM data from Cr-rich cores in the euhedral spinels plot mostly in the field of Cretaceous-Paleogene boundary spinel from Europe, North Africa and the Atlantic^28,30^ or in the field of Jurassic ablation spinel^29^, both of which clearly differ from the compositional fields of spinel in cosmic microscopic objects such as cosmic spherules. Small spherical spinels plot well outside all of these trends with a more trevolite-rich component than euhedral and dendritic spinels, suggesting that these spinels, with their high NiO concentrations, were derived from oxidation of Fe-Ni-rich metal. Both petrographic and geochemical lines of evidence indicate that these abundant spherules with pseudomorphs suggestive of porphyritic olivine, as well as spinel grains, were formed by a meteorite impact event on the Earth’s surface at ~11 Ma. On Fe*/(Mg + Fe) vs. Cr/(Al + Cr) diagrams (Fig. 4d,e), where the spinel grains in the spherules are compared with spinels in carbonaceous chondrites (CCs)^31^, ordinary chondrites (OCs)^32^ and Ordovician fossil meteorites^33^, spinel grains from PC11 exhibit a wide compositional range, as they do in the NiFe_2_O_4_–MgFe_2_O_4_–Fe_3_O_4_ ternary diagrams (Fig. 4b,c), due to partial melting. Rims of the spinel grains have high Fe*/(Mg + Fe) and low Cr/(Al + Cr) ratios reflecting a large magnetite component, whereas the core compositions overlap those of spinels in CCs and OCs (Fig. 4e). Because spinel compositions in CCs and OCs are indistinguishable from each other, the spinel compositions from the spherules are consistent with a CC or OC impactor.

Impact ejecta layers of middle Miocene age (11.7 Ma) have been locally recognised in offshore sediments near New Zealand and Antarctica^34^. If the impact ejecta layer reported here is synchronous with those, despite the uncertainty of ages based on Os isotope stratigraphy, the middle Miocene impact event may be a previously overlooked global event. However, no large crater of that age has been reported from land; indeed, only the Ries and Steinheim craters (ca. 15.1 Ma), with diameters less than 24 km, have been reported from the entire Miocene Epoch^35^. Further surveys of deep-sea sediments as well as on-land craters, combined with studies using multiple geochronometers, may produce more evidence bearing upon the origin of Miocene impact ejecta in the pelagic Pacific Ocean.

## Summary

A ~40 cm clayey layer in a sediment core from the Northwest Pacific Ocean exhibits high PGE concentrations, including values exceeding those for average upper continental crust by up to 3.3 times for Re and 50 times for Os. The core interval most enriched in Os has a conspicuous negative Os isotope anomaly as low as ~0.2 and coincides with the greatest PGE enrichments. Abundant spherules in this layer include pseudomorphs suggestive of porphyritic olivine as well as euhedral, dendritic and spherical spinel grains with NiO contents as great as 23.3 wt%. These petrographic and geochemical lines of evidence indicate the presence of extraterrestrial impact ejecta. A determination based on Os isotope stratigraphy yielded a most plausible depositional age of ~11 Ma for this ejecta layer. Because only two terrestrial craters of Miocene age (ca. 15.1 Ma) have been reported^35^, both of which are unlikely sources of this ejecta layer, additional surveys of deep-sea sediments in different parts of the world ocean will be needed to reveal more details of this possible impact event.

## Materials and Methods

### Samples

Our samples are from the 11th piston core (PC11) obtained during the cruise MR14-E02 by *R/V Mirai* in 2014 in the Exclusive Economic Zone around Minamitorishima Island^36–39^ at 154°00.9804′E, 22°59.0161′N and 5,647 m water depth (Supplementary Fig. S1). The PC11 core is 1,311.5 cm long and was divided onboard the ship into 13 sections plus the core catcher (CC) section (Supplementary Fig. S2). The onboard visual core description using smear slides classified the lithology of the core as follows. Sections 1 to 4 and the top of section 5 are composed of clay with trace amounts of zeolite and BCP. Section 5 contains a manganese-rich layer at 412.3–433.3 cmbsf, including sample PC11-5_24-26, beneath which the lithology changes to clay with more than 10% zeolite. Sections 6–9 consist of clay with zeolite, sections 10 and 11 consist of clay with more than 10% BCP and zeolite, section 12 consists of clay with zeolite, and sections 13 and CC consist of clay.

### Analytical methods

Micro-XCT analysis was conducted at JAMSTEC and Research Microscopy Solutions Tokyo, Carl Zeiss Co. To count and measure spherule grains, we used the micro-XCT instrument (Scan Xmate-DF160TSS105, Comscantecno Co.) installed at JAMSTEC, using X-ray tube voltage of 80 kV and current of ~45 μA, corresponding to a target current of ~10 μA. For higher resolution analysis, we used a micro-XCT instrument (Zeiss Xradia 520 Versa, Carl Zeiss Co.) installed at Research Microscopy Solutions Tokyo, Carl Zeiss Co., with an X-ray tube voltage of 60 kV and current of 84 μA (equivalent to 5 W energy). The coarse fractions (>62 μm) of five samples from the 349.9–359.9 cmbsf interval were sieved, mounted in acrylic resin (Acryl Monomer, Nichika Inc.) and prepared as polished sections. After observation under a reflected-light microscope and a scanning electron microscope (SEM; Hitachi Miniscope TM3000, Hitachi Inc.), the chemical compositions of each constituent mineral were determined by a field emission electron probe microanaliser (FE-EPMA; JEOL JXA-8530F) at Kyushu University using a 0.1 μm beam size, 15 kV acceleration voltage and 6 nA beam current. X-ray mapping by FE-EPMA was conducted with 15 kV accelerating voltage, 50 nA beam current, 1 μm beam size and 10 ms acquisition time per point. Selected spinel grains with high Cr_2_O_3_ concentrations were sliced into sections ~150 nm thick by focused ion beam (FIB-SEM; JEOL JIB-4501), and damage layers of the sections were removed by low-acceleration Ar ion milling (Fischione NanoMill 1040). TEM observations and chemical compositions were obtained with a TEM (FEI Tecnai G2-F20) equipped with an energy-dispersive X-ray spectrometer (EDS), and elemental distribution maps were obtained by a scanning TEM (JEOL JEM-3200FSK) equipped with EDS at the Ultramicroscopy Analysis Center, Kyushu University.

Sediment samples for bulk chemical analyses were dried at 40 °C and pulverised with an agate mortar and pestle until thoroughly homogenised. Then each sample was split, with one half used for major-element analysis and the other for trace-element analysis. Samples were further dried at 110 °C for ~12 h, and loss on ignition was determined from the loss in sample weight after ignition at 950 °C for over 6 h. Then bulk major-element concentrations were determined using the multiple glass bead method by X-ray fluorescence (XRF; Rigaku ZSX Primus II) at the University of Tokyo. Bulk trace-element compositions were measured by inductively coupled plasma quadrupole mass spectrometry (ICP-QMS; Agilent 7500c and iCAP-Q, Thermo Fisher Scientific) at the University of Tokyo through the mixed acid digestion method (HF, HClO_4_ and HNO_3_). Sample preparation and analytical procedures for bulk chemical analyses are detailed in refs^39–41^. Concentrations and isotope compositions of Re and Os were determined in powdered samples weighing ~1 g by the isotope dilution method combined with Carius tube (borate silica glass) digestion^42^, introduction of OsO_4_ by sparging^43–45^, followed by Os removal by drying the sample solution and Re purification in an anion exchange column^46^. The Os isotope ratio was measured by multi-collector ICP-MS (MC-ICP-MS; NEPTUNE, Thermo Fisher Scientific) using a multi-ion counter or Faraday cups^44,45^, and Re isotopes were measured by ICP-QMS (Agilent 7500ce) at JAMSTEC. 1RSD errors of the Os concentrations and Os isotope ratios are 0.17–0.84% and 0.12–1.67%, respectively. For selected intervals, concentrations of PGEs in powdered samples weighing ~0.1 g were determined by isotope dilution with Carius tube (quartz glass) digestion^47^. PGEs were purified by anion and cation exchange resins, and PGE concentrations and Re isotope ratios were measured by double-focusing single collector ICP-MS (DF-ICP-MS; ELEMENT-XR, Thermo Fisher Scientific) at the University of Tokyo. The Os concentration and isotope ratio were also independently determined by using thermal ionisation mass spectrometry (TIMS: TRITON and TRITON Plus, Thermo Fisher Scientific) at JAMSTEC. 2RSE errors of the Os concentrations and Os isotope ratios are 0.45–7.51% and 1.79–7.35%, respectively. These chemical compositions are listed in Supplementary Tables S1–S5.

Concentrations and isotopic compositions of Re and Os were determined using two different analytical procedures on the same samples, and the results were nearly equivalent within their respective error ranges. Most of the Re-Os data were plotted on the direct proportion line with a slope of 1 except for sample PC11-4_62-64, which had the highest Os concentration and the most unradiogenic Os isotope ratio (Supplementary Fig. S11). Approximately 1 g of powdered sample material was used for the analyses by the sparging method, MC-ICP-MS and ICP-QMS, and ~0.1 g of powdered sample material was used for PGE and Os isotope analyses by solvent extraction of Re and Os, TIMS and DF-ICP-MS. Thus, we attribute the relatively large discrepancy for results from sample PC11-4_62-64 to inhomogeneous distribution (nugget effect) of PGEs in the sample, which is commonly observed even in reference rock materials^22^ and is consistent with the sparse distribution of large spherule grains shown by the micro-XCT image (Supplementary Fig. S4). Moreover, the larger errors in the results from solvent extraction, TIMS and DF-ICP-MS arise from the relatively large Os blank contribution to the relatively small samples.

### Os isotope dating and extraterrestrial Os mass accumulation rate

Ages determined by Os isotope stratigraphy are relative rather than numerical ages, and are determined by their fit with the record of secular changes in the global Os isotope composition of seawater^11–14^. These ages can be further constrained based on lithological features and thickness of strata^11^. However, pelagic clays commonly have hiatuses that represent periods of extremely slow or no sedimentation, which may result in imprecise or incorrect age assignments. We identified the age of individual samples by comparing their ^187^Os/^188^Os ratios with the marine ^187^Os/^188^Os curve and making slight adjustments to accommodate unusual changes in sedimentation rates.

Figure 1b shows that core PC11 contains a manganese-rich layer at 19–40 cm in section 5 (412.3–433.3 cmbsf), including sample PC11-5_24-26, with lithological changes indicating at least one hiatus. We assumed continuous sedimentation in the intervals above and below the manganese layer.

In the interval above the manganese layer, the depositional ages of the uppermost sample (PC11-1_4-6) and the two lowermost samples (PC11-5_4-6 and PC11-5_14-16) were dated at 2.0, 12.0 and 14.8 Ma, respectively, by fitting the seawater Os isotope curve. When fitting the secular change curve, we relied first on the seawater Os isotope curve based on pelagic sediment^18,48–55^. In intervals without Os isotope data from pelagic sediment, we used Os isotope compositions of Fe-Mn crusts in the Pacific Ocean^56,57^. Depositional ages of the 30 intervening samples were calculated by linear extrapolation along with their stratigraphic positions (Fig. 1b). The sharp negative Os isotope excursion in section 4 was thus assigned a date of ~11 Ma.

Below the manganese layer, a broad negative Os isotope shift was assigned to the Eocene-Oligocene boundary rather than the late Eocene meteorite impact event, which could not have produced a sustained negative Os isotope anomaly over the time span required to deposit ~7.5 m of clay. Seven samples in this interval were assigned ages by fitting with the secular curve: PC11-5_54-56 (32.5 Ma), PC11-6_4-6 (33.0 Ma), PC11-6_54-56 (33.4 Ma), PC11-7_4-6 (33.7 Ma), PC11-8_4-6 (34.1 Ma), PC11-8_54-56 (34.2 Ma) and PC11-13_54-56 (34.9 Ma). All other samples were dated by interpolation. Sample PC11-5_24-26, in the manganese layer, was tentatively assigned an age of 23.7 Ma at the midpoint between samples PC11-5_14-26 (14.8 Ma) and PC11-6_4-6 (32.5 Ma).

In sample PC11-4_62-64, having a sedimentation rate of 0.04 cm/kyr based on Os isotope dating, a dry density of ~0.5 g/cm^3^ and an excess Os (extraterrestrial Os) concentration of 1,366 ppt, the accumulation rate of extraterrestrial Os was calculated to be 27.3 ng/cm^2^/Myr, which is much higher than the background cosmic spherule flux of 4 ng/cm^2^/Myr^58^. Our Os-dating method yields a relative age instead of an absolute age and does not offer sufficient accuracy and precision for quantitative discussions. Nonetheless, we consider it unlikely for a cosmic spherule shower event to produce such a thick clay layer characterised by abundant spherules and spinel grains, high PGE concentrations and unradiogenic Os isotope compositions.

## Supplementary information

Supplementary Information
