# Supplementary material for: A Miocene impact ejecta layer in the pelagic Pacific Ocean
Source: Sci Rep. 2019 Nov 20;9:16111. doi: 10.1038/s41598-019-52709-1 (PMC6868271; doi:10.1038/s41598-019-52709-1)
Supplement: Supplementary file 1 — Supplementary Information [file 41598_2019_52709_MOESM1_ESM.pdf]

A Miocene impact ejecta layer in the pelagic Pacific Ocean

Tatsuo Nozaki[1,2,3,4], Junichiro Ohta[4,2,5,6], Takaaki Noguchi[7], Honami Sato[4,1], Akira Ishikawa[8,1,4], Yutaro Takaya[9,1,4], Jun-Ichi Kimura[6], Qing Chang[6], Kazuhiko Shimada[10], Jun-ichiro Ishibashi[10], Kazutaka Yasukawa[2,5,4], Katsunori Kimoto[11], Koichi Iijima[1] & Yasuhiro Kato[2,5,1,4]

[1]Submarine Resources Research Center, Research Institute for Marine Resources Utilization, Japan Agency for Marine-Earth Science and Technology (JAMSTEC), 2-15 Natsushima-cho, Yokosuka, Kanagawa 237-0061, Japan

[2]Frontier Research Center for Energy and Resources (FRCER), School of Engineering, The University of Tokyo, 7-3-1 Hongo, Bunkyo-ku, Tokyo 113-8656, Japan

[3]Department of Planetology, Kobe University, 1-1 Rokkodai-cho, Nada-ku, Kobe, Hyogo 657-8501, Japan

[4]Ocean Resources Research Center for Next Generation, Chiba Institute of Technology, 2-17-1 Tsudanuma, Narashino, Chiba 275-0016, Japan

[5]Department of Systems Innovation, School of Engineering, The University of Tokyo, 7-3-1 Hongo, Bunkyo-ku, Tokyo 113-8656, Japan

[6]Volcanoes and Earth's Interior Research Center, Research Institute for Marine Geodynamics, Japan Agency for Marine-Earth Science and Technology (JAMSTEC), 2-15 Natsushima-cho, Yokosuka, Kanagawa 237-0061, Japan

[7]Division for Experimental Natural Science, Faculty of Arts and Science, Kyushu University, 744 Motooka, Nishi-ku, Fukuoka 819-0395, Japan

[8]Department of Earth and Planetary Sciences, Tokyo Institute of Technology, 2-12-1 Ookayama, Meguro-ku, Tokyo 152-8550, Japan

[9]Department of Resources and Environmental Engineering, School of Creative Science and Engineering, Waseda University, 3-4-1 Okubo, Shinjuku-ku, Tokyo 169-8555, Japan

[10]Department of Earth and Planetary Sciences, Faculty of Science, Kyushu University, 744 Motooka, Nishi-ku, Fukuoka 819-0395, Japan

[11]Earth Surface System Research Center, Research Institute for Global Change, Japan Agency for Marine-Earth Science and Technology (JAMSTEC), 2-15 Natsushima-cho, Yokosuka, Kanagawa 237-0061, Japan

Correspondence and requests for materials should be addressed to T.Noz. (email: nozaki@jamstec.go.jp)

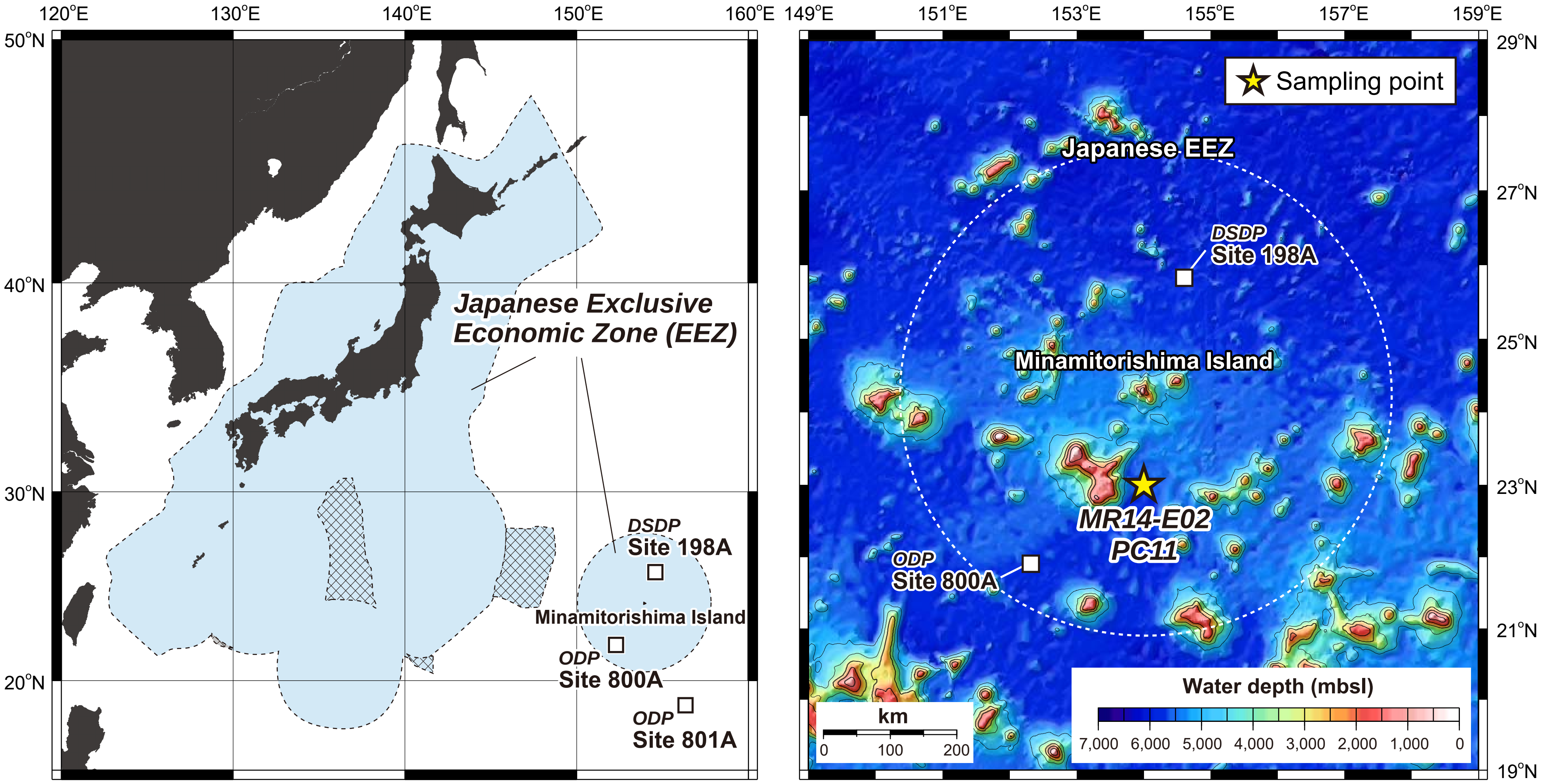

Supplementary Fig. S1 Location and bathymetric map of the core site. The Japanese Exclusive Economic Zone with Deep Sea Drilling Project and Ocean Drilling Program drill sites is also shown. Modified from refs. 36 and 37. Bathymetric map was generated using GMT (Generic Mapping Tools: http://gmt.soest.hawaii.edu/home).

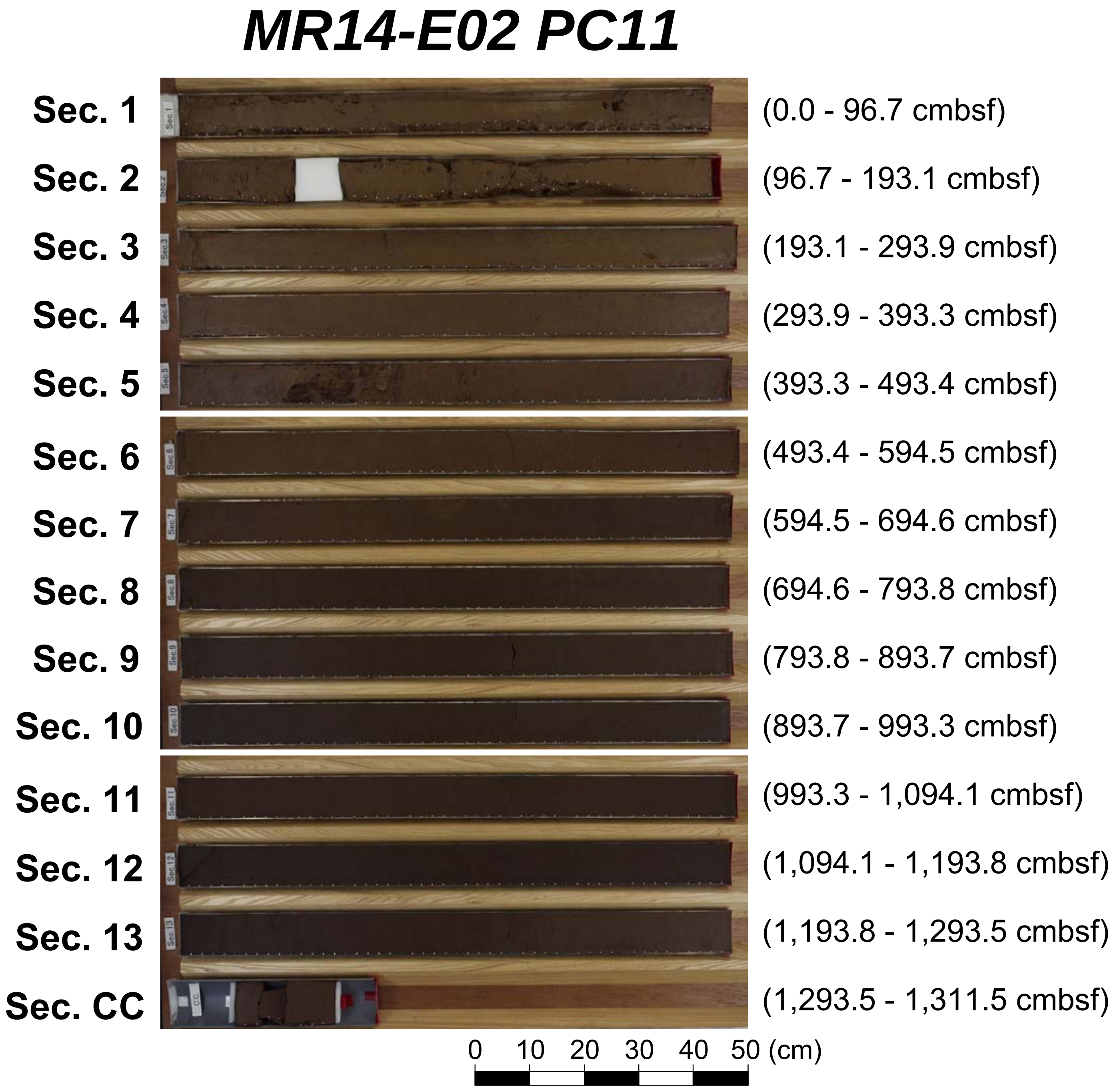

Supplementary Fig. S2 Photograph of PC11 segments. Anomalous PGE enrichment, negative Os isotope excursion and abundant spherules were found in Section 4.

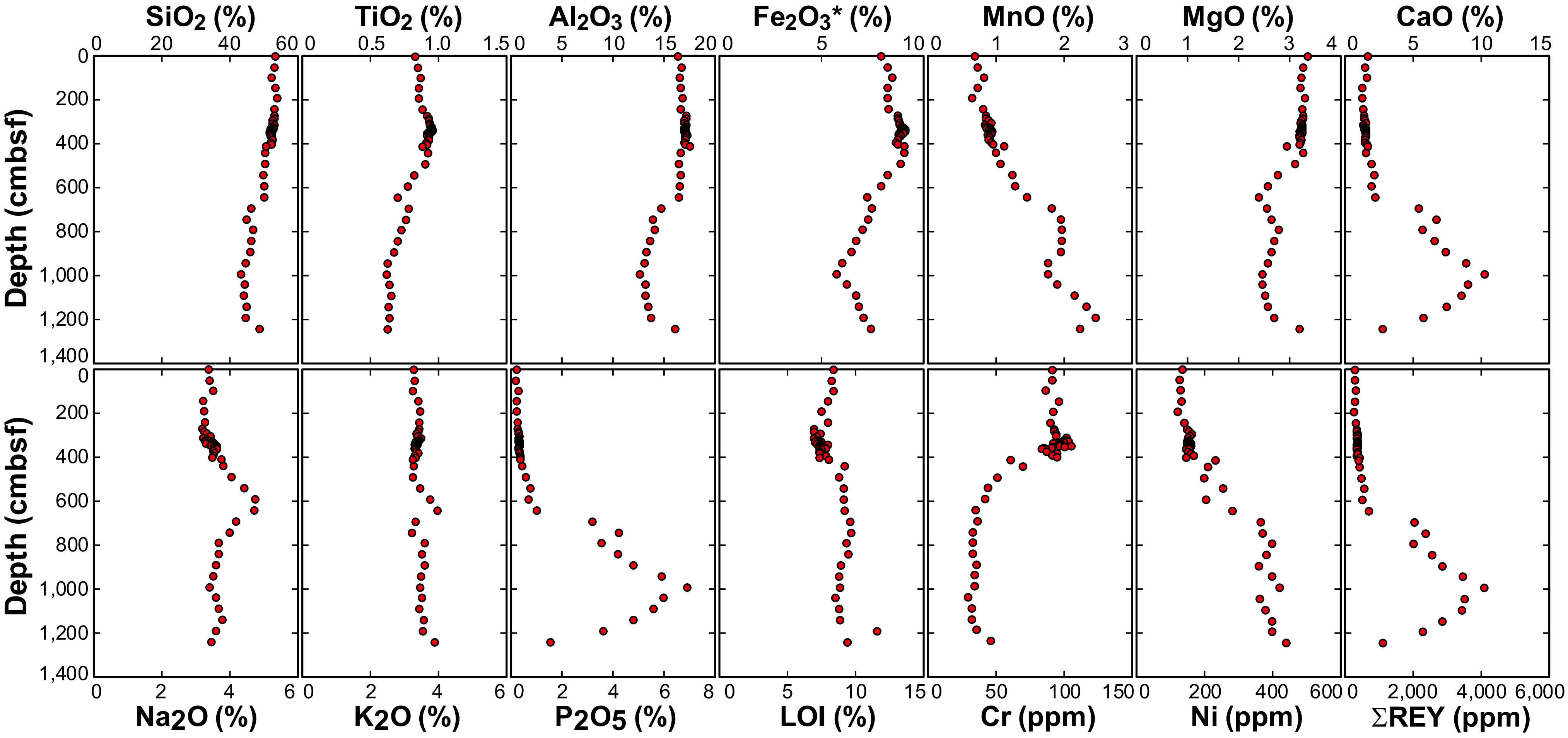

Supplementary Fig. S3 Depth profiles of major elements, loss on ignition (LOI) and trace elements in PC11. There is no clear anomaly associated with the spherule-rich layer except for a small peak in Cr concentration. Fe_2O_3*, total iron as Fe_2O_3.

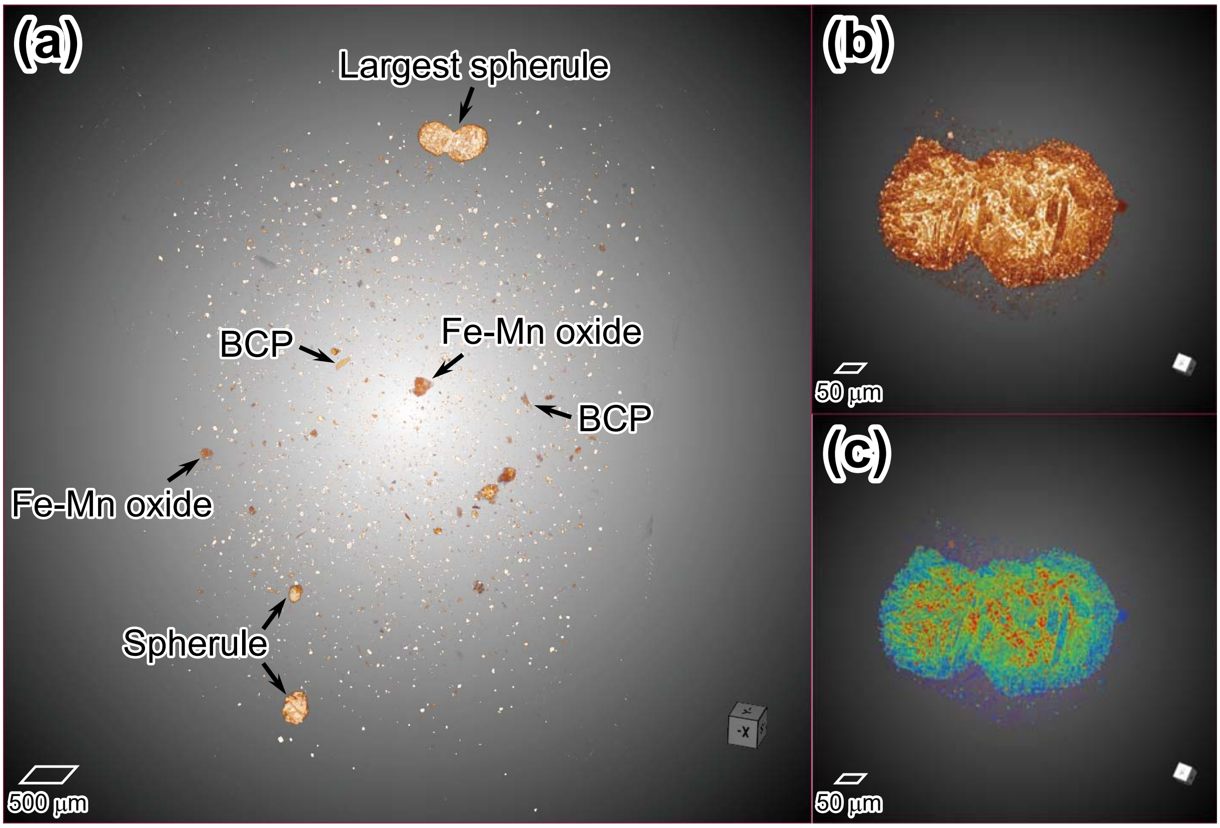

Supplementary Fig. S4 Micro-XCT images. The wet 0.211 g subsample of sample PC11-4_62-64, with the highest PGE concentration and most unradiogenic Os isotope composition, was used for the micro-XCT analysis. (**a**) Image of 3D model with a resolution of 4.6 μm/voxel. (**b**) Cross-section image of the largest spherule grain in (**a**) with a resolution of 1.0 μm/voxel. (**c**) Same cross-section as (**b**) imaged using a different colour gradation. BCP, biogenic calcium phosphate.

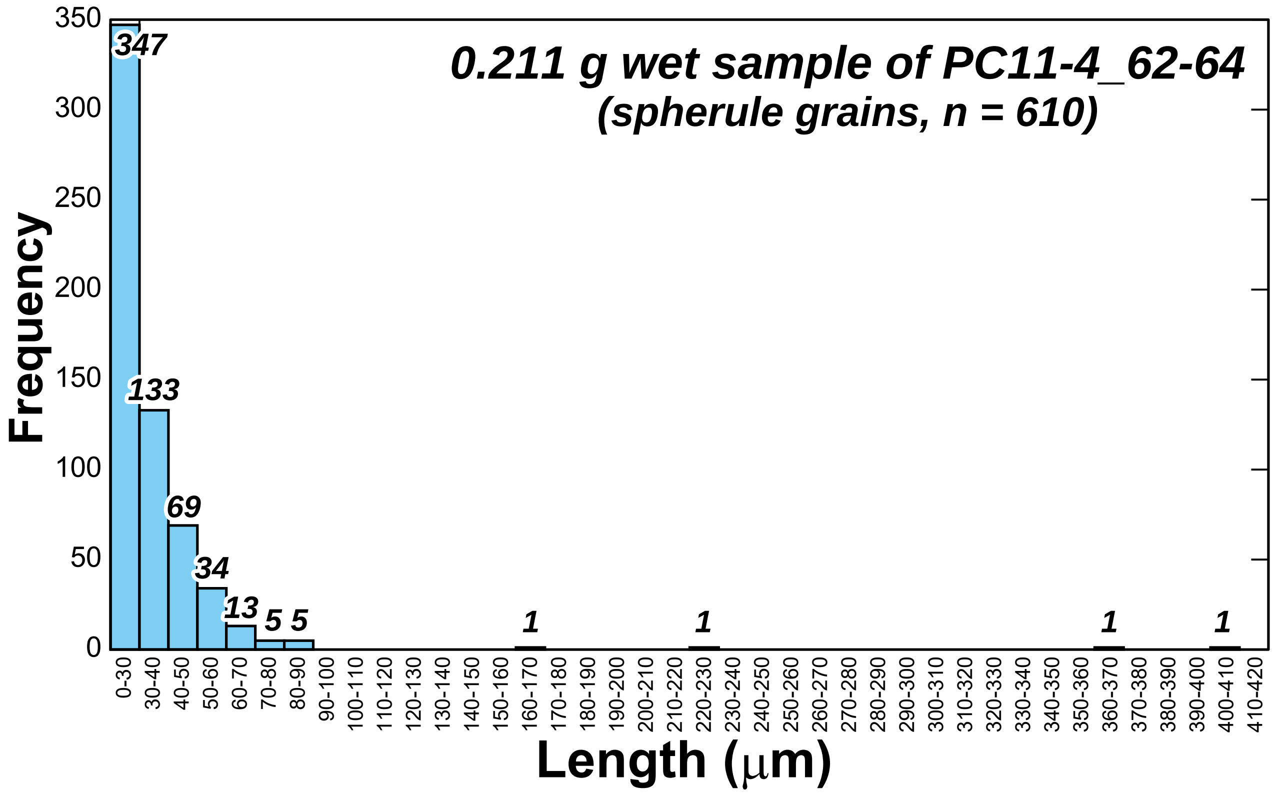

Supplementary Fig. S5 Histogram of the longest dimension of spherule grains identified in the micro-XCT analysis.

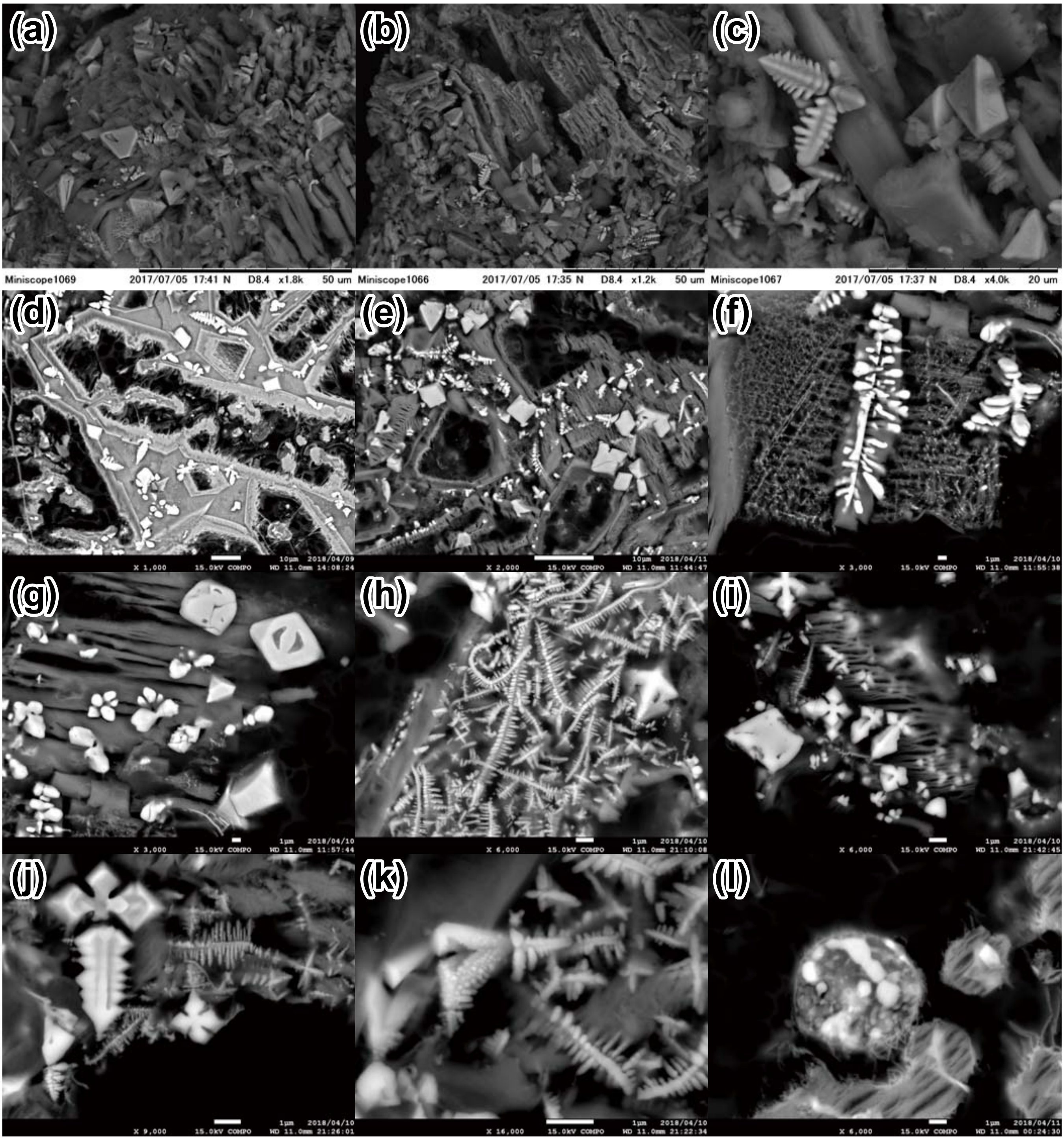

Supplementary Fig. S6 SEM and BSE images of spinel grains in the spherules. (**a–c**) SEM images of euhedral and dendritic spinel within olivine pseudomorphs replaced by clay minerals. (**c**) is a detail of (**b**). (**d, e**) BSE images of spinel grains concentrated on the outer rim of the pseudomorphs. (**f–k**) BSE images showing various shapes owing to partial melting and quenching. (**l**) BSE image of minor spherical spinel grains.

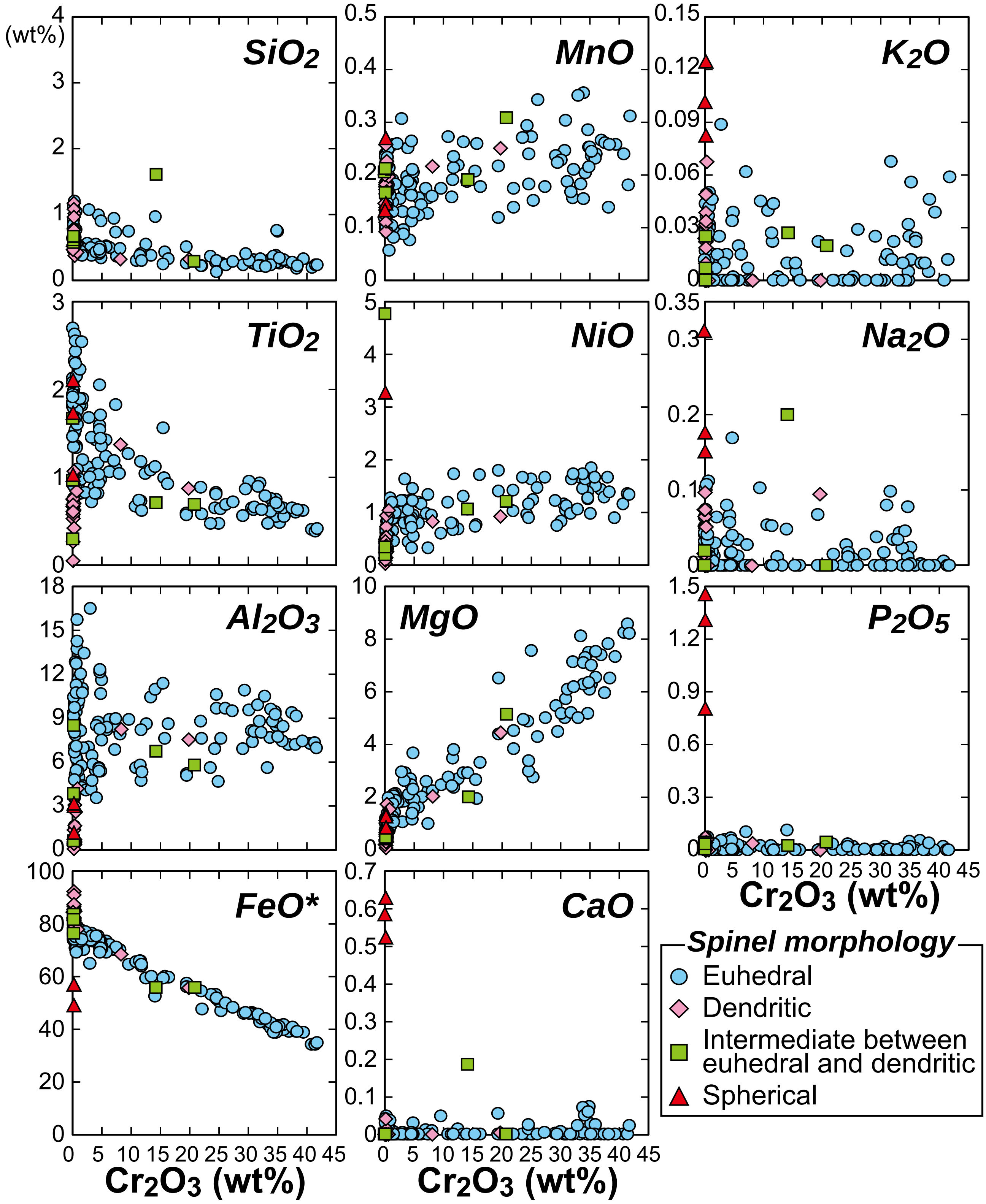

Supplementary Fig. S7 Diagrams of Cr_2O_3 vs. major oxides (wt%) for spinel grains in spherules. FeO*, total iron as FeO.

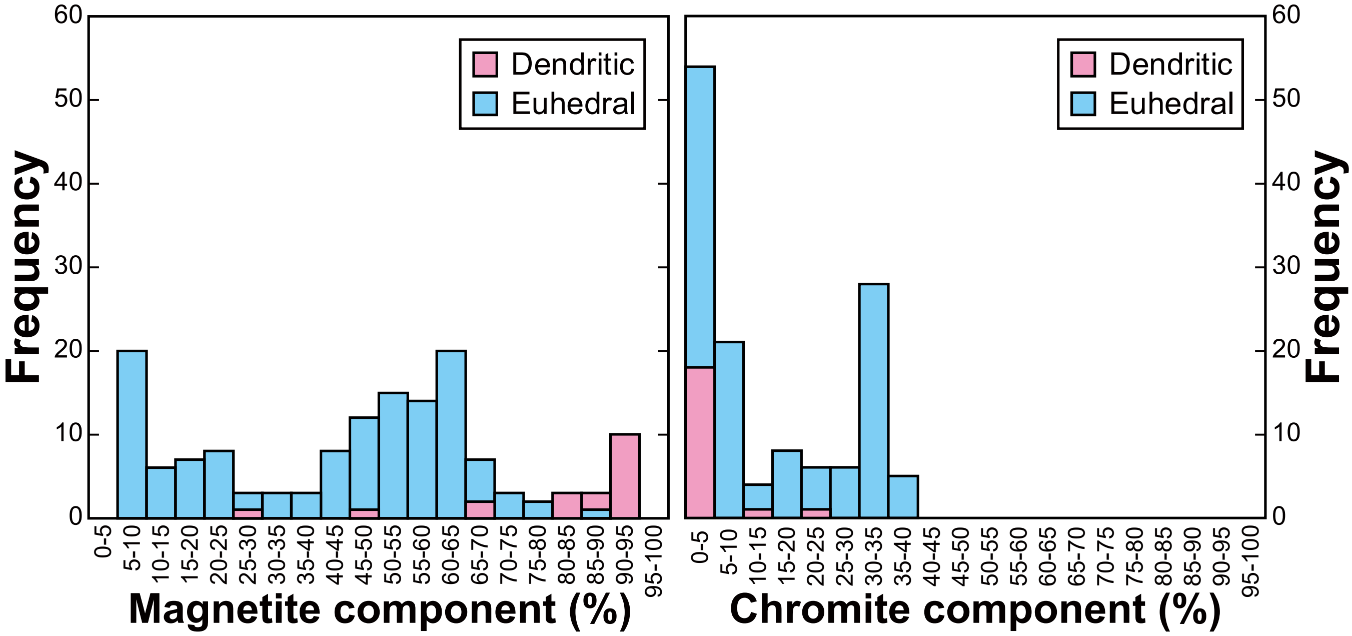

Supplementary Fig. S8 Histograms of magnetite and chromite components in spinel grains. EPMA data with higher than 80% total wt% were selected and calculated by assuming a spinel stoichiometry (AB_2O_4) for seven constituent cations (Mg, Al, Fe, Cr, Ni, Mn and Ti). Most of dendritic spinels are composed of higher than 80% of magnetite component.

MR14-E02 PC11-4_56-58

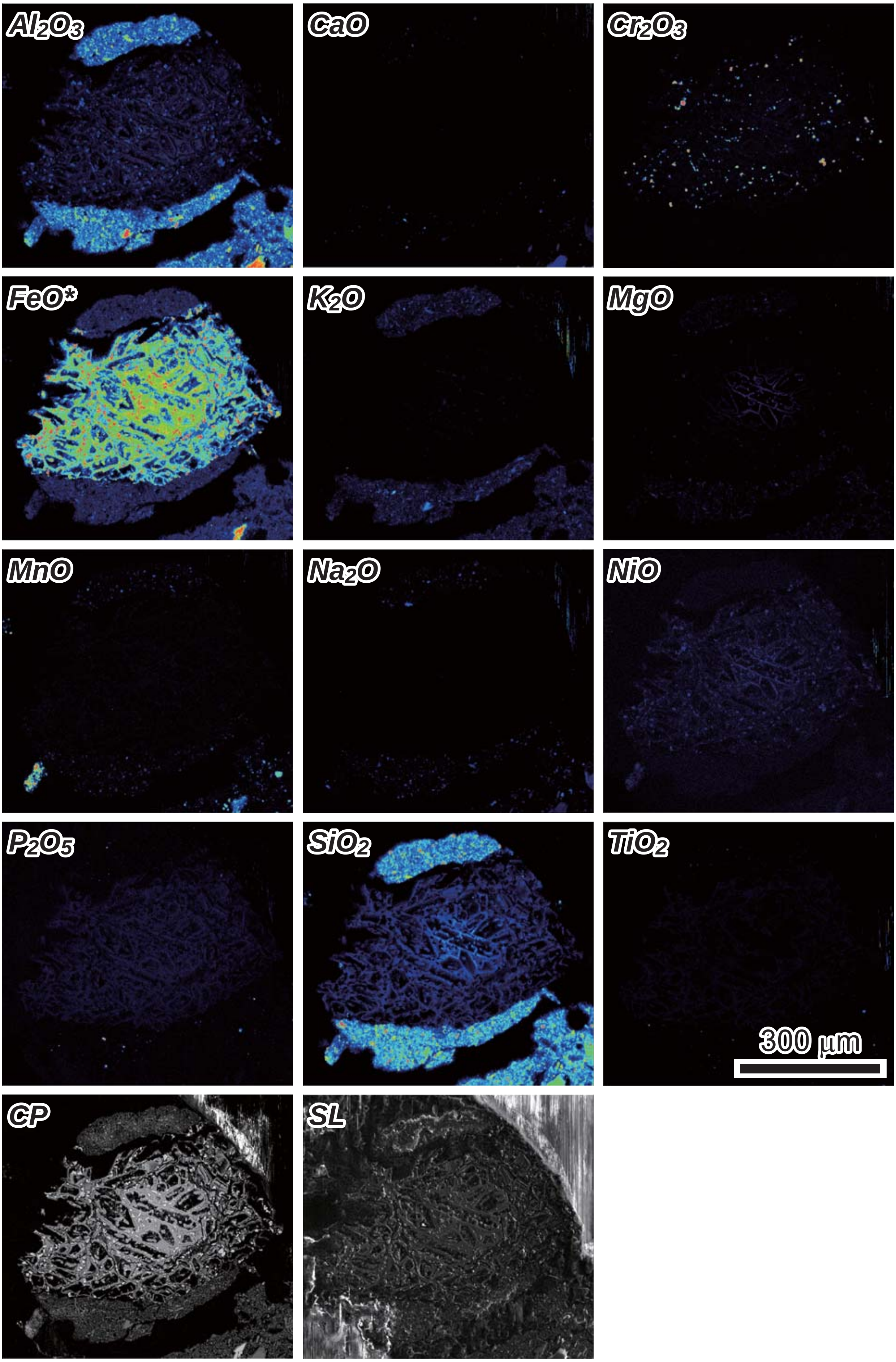

Supplementary Fig. S9 EPMA mapping data for a spinel-rich spherule from sample PC11-4_56-58. FeO*, total iron as FeO; CP, composition; SL, SEM level.

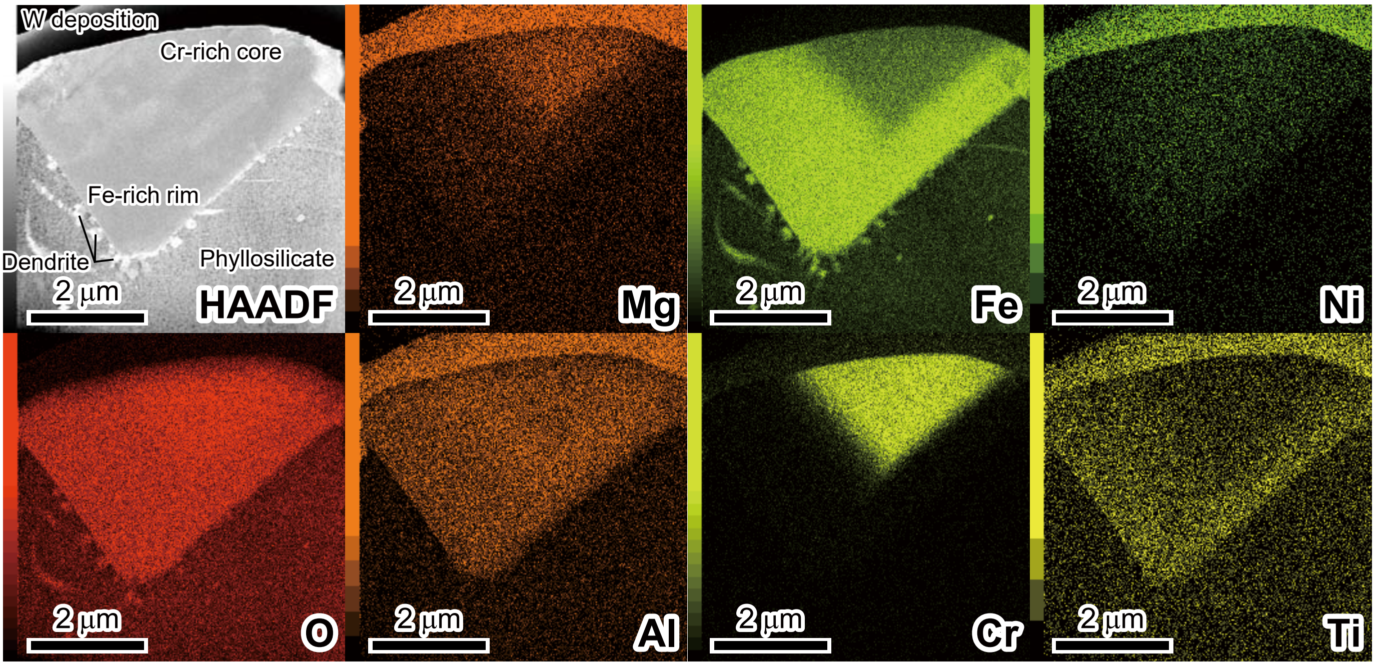

Supplementary Fig. S10 TEM-EDS elemental mapping data for a chromite component-rich spinel grain from sample PC11-4_56-58. HAADF, high-angle annular dark field–scanning transmission electron microscopy image. The HAADF, Fe and Cr images also appear in text Fig. 4a.

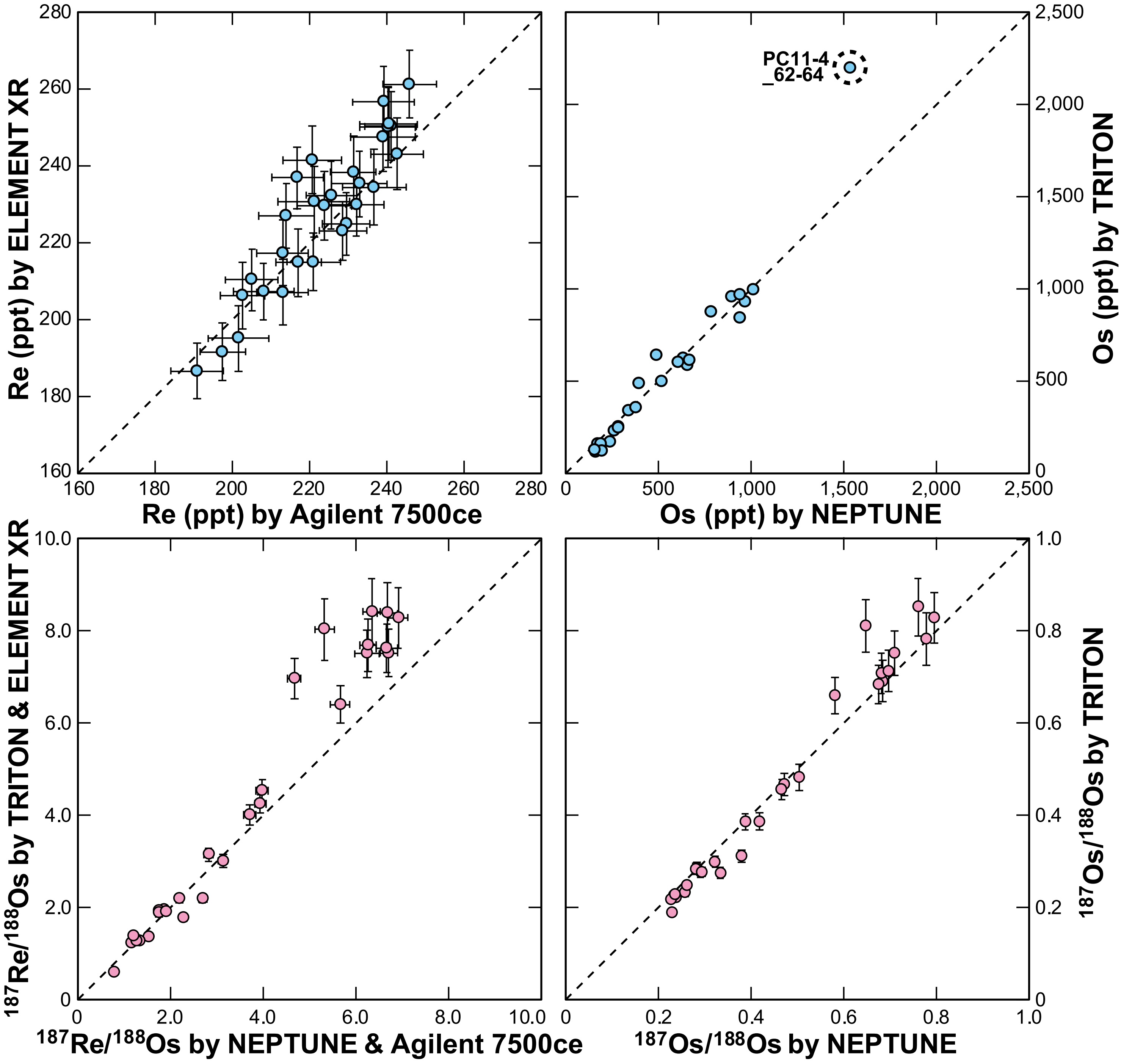

Supplementary Fig. S11 Comparison of Re-Os data obtained by two different analytical methods and spike solutions. One dataset (Supplementary Table S1) was measured by the sparging method, MC-ICP-MS and ICP-QMS, and the other dataset (Supplementary Table S3) was measured by solvent extraction of Re and Os, TIMS and DF-ICP-MS with different spike solutions. Dashed lines show the direct proportion lines with a slope of 1.

Table S1: Re-Os concentrations and isotope ratios from core MR14-E02 PC11. See text for analytical procedures

Sample No.	Depth (cmbsf)	Weight (g)	Re (ppt)	1SD	Os (ppt)	1SD	$^{187}Re/^{188}Os$	1SD	$^{187}Os/^{188}Os$	1SD
PC11-1_4-6	5.0	1.00898	232	6	109.9	0.7	11.2	0.3	0.927	0.008
PC11-2_4-6	101.7	1.02050	264	7	133.2	0.6	10.48	0.26	0.871	0.005
PC11-3_4-6	198.1	1.01626	202	6	110.5	0.7	9.64	0.27	0.835	0.007
PC11-3_84-86	278.1	1.01666	197	6	162.3	0.7	6.34	0.19	0.761	0.003
PC11-3_94-96	288.1	1.04378	205	7	160.4	0.6	6.68	0.22	0.7790	0.0026
PC11-4_4-6	298.9	1.02589	229	6	176.8	0.8	6.71	0.18	0.684	0.004
PC11-4_14-16	308.9	1.00209	217	7	237.0	0.6	4.67	0.14	0.5822	0.0019
PC11-4_24-26	318.9	1.01429	191	7	261.1	0.6	3.70	0.13	0.5038	0.0014
PC11-4_34-36	328.9	1.04003	213	7	340.6	0.9	3.13	0.10	0.4187	0.0018
PC11-4_36-38	330.9	1.00635	226	7	491.2	1.0	2.27	0.07	0.3338	0.0009
PC11-4_38-40	332.9	1.02305	213	7	395.1	0.9	2.68	0.08	0.3801	0.0013
PC11-4_40-42	334.9	1.00954	214	7	378.8	0.8	2.81	0.09	0.3873	0.0011
PC11-4_42-44	336.9	1.00883	239	8	633.6	1.4	1.86	0.06	0.2909	0.0007
PC11-4_44-46	338.9	1.03428	232	7	657.3	1.5	1.74	0.05	0.2833	0.0006
PC11-4_46-48	340.9	1.02613	237	8	668.2	1.1	1.74	0.06	0.2813	0.0006
PC11-4_48-50	342.9	1.01083	233	7	606.1	1.1	1.89	0.06	0.2939	0.0008
PC11-4_50-52	344.9	1.00938	228	6	516.9	1.1	2.18	0.06	0.3224	0.0009
PC11-4_52-54	346.9	1.06320	243	7	783.1	1.6	1.52	0.04	0.2582	0.0006
PC11-4_54-56	348.9	1.02661	242	6	854.5	2.7	1.38	0.04	0.2398	0.0006
PC11-4_54-56_dup1	348.9	1.03163	240	7	961.4	2.2	1.22	0.04	0.2298	0.0004
PC11-4_54-56_dup2	348.9	1.03466	242	7	868.7	1.7	1.36	0.04	0.2411	0.0004
PC11-4_56-58	350.9	1.05233	239	8	969.5	1.9	1.20	0.04	0.2331	0.0006
PC11-4_58-60	352.9	1.01189	240	7	1,015.3	2.2	1.15	0.03	0.2274	0.0004
PC11-4_60-62	354.9	1.01383	240	7	942.5	1.7	1.25	0.04	0.2613	0.0006
PC11-4_62-64	356.9	1.03564	246	7	1,536.4	3.0	0.781	0.022	0.22870	0.00026
PC11-4_64-66	358.9	1.03740	234	5	927.4	2.4	1.234	0.028	0.236	0.0004
PC11-4_64-66_dup1	358.9	1.00112	228	6	951.2	1.7	1.17	0.03	0.233	0.0004
PC11-4_66-68	360.9	1.00548	224	7	283.5	2.4	3.97	0.13	0.4718	0.0028
PC11-4_68-70	362.9	1.02155	221	9	183.1	0.7	6.24	0.26	0.682	0.004
PC11-4_70-72	364.9	1.00844	221	7	282.7	0.6	3.93	0.12	0.4667	0.0018
PC11-4_72-74	366.9	1.17474	221	8	171.3	0.7	6.66	0.23	0.698	0.003
PC11-4_74-76	368.9	1.01271	217	6	179.7	1.0	6.26	0.17	0.710	0.004
PC11-4_84-86	378.9	1.17520	208	8	189.7	0.5	5.66	0.21	0.6753	0.0024
PC11-4_94-96	388.9	1.00642	202	8	195.0	0.6	5.32	0.21	0.6481	0.0024
PC11-5_4-6	398.3	1.02077	203	6	153.6	0.8	6.91	0.20	0.796	0.005
PC11-5_14-16	408.3	1.01416	212	6	186.7	0.6	5.91	0.17	0.7323	0.0026
PC11-5_24-26	418.3	1.00772	649	23	197.5	0.7	16.9	0.6	0.629	0.003
PC11-5_54-56	448.3	1.01411	278	8	194.6	0.8	7.32	0.22	0.619	0.003
PC11-6_4-6	498.4	1.02041	334	11	154.1	0.7	11.0	0.4	0.529	0.004
PC11-6_54-56	548.4	1.02614	341	11	132.6	0.7	12.9	0.4	0.466	0.005
PC11-7_4-6	599.5	1.02373	329	7	99.3	0.6	16.7	0.4	0.488	0.006
PC11-7_54-56	649.5	1.02352	253	6	136.7	0.6	9.19	0.22	0.373	0.004
PC11-8_4-6	699.6	1.02202	188	5	107.1	0.6	8.72	0.26	0.378	0.004
PC11-8_54-56	749.6	1.02445	165	5	126.6	0.6	6.44	0.20	0.307	0.003
PC11-9_4-6	798.8	1.04781	154	5	73.9	0.5	10.4	0.3	0.380	0.005
PC11-9_54-56	848.8	1.02602	146	4	67.6	0.5	10.8	0.3	0.415	0.006
PC11-10_4-6	898.7	1.02898	122	4	95.0	0.5	6.34	0.22	0.315	0.004
PC11-10_54-56	948.7	1.03494	114	4	72.4	0.5	7.8	0.3	0.363	0.006
PC11-11_4-6	998.3	1.02688	112	4	78.6	0.5	7.05	0.23	0.305	0.005
PC11-11_54-56	1,048.3	1.04418	120	4	74.2	0.5	8.05	0.29	0.346	0.005
PC11-12_4-6	1,099.1	1.02340	127	4	95.5	0.5	6.55	0.23	0.322	0.004
PC11-12_54-56	1,149.1	1.02314	115	4	53.2	0.4	10.7	0.4	0.375	0.006
PC11-13_54-56	1,248.8	1.01597	101	3	92.7	0.5	5.43	0.18	0.364	0.004

All data are blank corrected with error propagation. Errors are determined on the basis of the results of refs. 44 and 45.

Table S2: Major- and trace-element compositions from core MR14-E02 PC11 determined by XRF and ICP-QMS

Element	Unit	PC11-1_4-6	PC11-1_54-56	PC11-2_4-6	PC11-2_54-56	PC11-3_4-6	PC11-3_54-56	PC11-3_84-86	PC11-3_94-96	PC11-4_4-6†	PC11-4_14-16	PC11-4_24-26
Depth	cmbsf	5.0	55.0	101.7	151.7	198.1	248.1	278.1	288.1	298.9	308.9	318.9
SiO_2	wt%	53.63	53.37	52.74	53.87	54.21	53.57	53.50	53.48	53.00	53.01	53.46
TiO_2	wt%	0.83	0.85	0.87	0.86	0.86	0.88	0.92	0.93	0.93	0.94	0.94
Al_2O_3	wt%	16.48	16.79	16.64	16.78	16.91	16.74	17.31	17.27	17.14	17.08	17.24
Fe_2O_3*	wt%	7.94	8.29	8.51	8.29	8.26	8.34	8.79	8.78	8.84	8.87	8.85
MnO	wt%	0.70	0.73	0.83	0.73	0.66	0.82	0.85	0.85	0.89	0.93	0.84
MgO	wt%	3.38	3.28	3.25	3.24	3.32	3.27	3.29	3.29	3.26	3.24	3.27
CaO	wt%	1.68	1.46	1.63	1.30	1.26	1.32	1.39	1.41	1.45	1.52	1.37
Na_2O	wt%	3.39	3.41	3.51	3.22	3.24	3.27	3.19	3.25	3.32	3.43	3.21
K_2O	wt%	3.30	3.33	3.27	3.43	3.47	3.45	3.46	3.44	3.37	3.40	3.51
P_2O_5	wt%	0.24	0.22	0.31	0.23	0.22	0.28	0.29	0.30	0.32	0.34	0.30
LOI	wt%	8.43	8.28	8.45	8.05	7.59	8.05	7.01	7.01	7.47	7.23	7.00
Sc	ppm	20.95	21.69	21.88	21.11	20.12	20.66	22.20	22.87	23.39	24.12	23.50
V	ppm	164.66	171.24	168.85	173.32	164.13	165.20	176.11	182.32	190.38	185.66	188.07
Cr	ppm	91.34	91.65	86.38	96.72	92.31	89.95	92.96	93.18	94.26	94.08	101.79
Co	ppm	70.77	71.05	77.00	70.68	62.68	77.85	84.46	86.37	91.16	96.16	88.32
Ni	ppm	134.93	127.68	129.71	131.63	121.77	140.84	150.10	154.60	162.37	158.14	152.91
Cu	ppm	207.86	206.90	230.18	200.33	192.99	235.01	248.53	256.17	263.51	264.32	252.21
Zn	ppm	146.60	144.58	141.46	143.74	139.89	143.42	143.06	148.15	151.73	147.70	154.55
As	ppm	19.50	22.60	23.66	24.07	22.38	23.57	22.59	22.64	23.98	25.62	24.83
Rb	ppm	133.92	137.41	128.09	142.57	136.91	134.04	129.06	131.11	132.02	131.26	138.28
Sr	ppm	171.97	173.02	180.33	167.88	154.22	164.31	174.65	182.15	187.06	191.82	178.81
Y	ppm	45.94	44.71	55.53	48.50	43.85	54.79	60.92	63.97	68.97	73.62	65.11
Zr	ppm	105.74	111.12	116.67	117.21	112.16	119.16	130.29	134.76	139.26	137.65	139.28
Nb	ppm	13.79	14.46	14.58	15.29	14.79	15.61	16.16	16.58	16.55	16.39	16.94
Mo	ppm	9.18	15.02	18.82	21.82	19.81	22.87	25.36	26.52	27.70	28.74	27.18
Cs	ppm	13.08	13.55	12.76	13.92	12.96	13.40	12.99	13.12	12.94	12.65	13.17
Ba	ppm	756.12	731.97	627.39	680.58	674.83	611.91	618.62	591.14	547.52	528.47	576.50
La	ppm	42.00	42.64	46.38	45.18	42.15	47.45	54.15	54.41	57.17	59.04	56.68
Ce	ppm	91.86	92.28	93.23	97.37	89.93	95.73	105.36	108.27	111.61	111.01	112.36
Pr	ppm	11.91	12.03	13.14	12.60	11.71	13.40	13.34	13.44	14.33	14.70	14.04
Nd	ppm	44.08	44.42	49.06	46.81	43.12	50.19	53.70	54.40	59.22	59.75	56.30
Sm	ppm	10.23	10.09	11.33	10.53	9.78	11.54	11.49	11.44	12.67	13.01	11.87
Eu	ppm	2.33	2.34	2.66	2.45	2.21	2.65	2.64	2.74	2.93	3.05	2.79
Gd	ppm	10.02	9.85	11.50	10.38	9.54	11.60	11.46	11.73	12.69	13.32	12.04
Tb	ppm	1.51	1.50	1.74	1.57	1.44	1.76	1.71	1.77	1.94	2.03	1.80
Dy	ppm	9.26	9.15	10.70	9.65	8.80	10.81	10.66	11.16	11.94	12.76	11.25
Ho	ppm	1.87	1.84	2.20	1.98	1.80	2.21	2.17	2.21	2.40	2.59	2.27
Er	ppm	5.39	5.25	6.38	5.78	5.27	6.42	6.34	6.56	7.05	7.40	6.74
Tm	ppm	0.78	0.77	0.91	0.82	0.75	0.92	0.88	0.92	1.00	1.06	0.95
Yb	ppm	4.99	4.99	5.96	5.35	4.92	5.97	5.87	6.08	6.47	6.78	6.12
Lu	ppm	0.78	0.76	0.92	0.83	0.75	0.92	0.90	0.94	1.01	1.05	0.95
Hf	ppm	2.85	2.96	3.06	3.15	3.03	3.22	2.95	2.90	2.99	2.84	2.98
Ta	ppm	0.98	1.02	0.99	1.08	1.06	1.10	0.94	0.91	0.92	0.89	0.96
Pb	ppm	33.19	34.96	36.67	35.51	33.24	36.24	34.50	34.20	36.06	35.18	34.11
Th	ppm	14.41	14.45	13.97	15.13	14.38	14.93	11.84	11.55	11.86	11.34	11.85
U	ppm	2.44	2.54	2.55	2.68	2.64	2.68	2.20	2.12	2.26	2.15	2.24
ΣREY	ppm	282.96	282.61	311.63	299.79	276.01	316.37	341.61	350.04	371.41	381.17	361.27

*, Total iron as Fe_2O_3; LOI, loss on ignition.

† Although compositions of these samples were reported by ref. 39, they were re-measured in this study along with the neighboring samples. Reproducibilities of replicate analyses were typically within 5%.

Table S2 (continued)

Element	PC11-4_34-36	PC11-4_36-38	PC11-4_38-40	PC11-4_40-42	PC11-4_42-44	PC11-4_44-46	PC11-4_46-48	PC11-4_48-50	PC11-4_50-52	PC11-4_52-54	PC11-4_54-56†	PC11-4_56-58
Depth	328.9	330.9	332.9	334.9	336.9	338.9	340.9	342.9	344.9	346.9	348.9	350.9
SiO_2	53.04	52.77	53.09	53.14	52.44	52.56	52.45	52.67	52.83	52.48	52.11	52.49
TiO_2	0.95	0.95	0.95	0.95	0.95	0.95	0.96	0.96	0.95	0.95	0.93	0.95
Al_2O_3	17.18	17.12	17.21	17.22	17.12	17.09	17.05	17.14	17.21	17.08	17.10	17.12
Fe_2O_3*	8.94	9.05	9.01	8.99	9.08	9.08	9.16	9.10	9.08	9.12	8.92	9.04
MnO	0.86	0.91	0.88	0.86	0.93	0.91	0.93	0.92	0.90	0.94	0.92	0.95
MgO	3.27	3.26	3.26	3.27	3.25	3.26	3.25	3.24	3.27	3.23	3.26	3.24
CaO	1.42	1.49	1.45	1.43	1.52	1.50	1.52	1.50	1.48	1.54	1.50	1.55
Na_2O	3.32	3.39	3.27	3.28	3.48	3.49	3.50	3.38	3.30	3.48	3.57	3.50
K_2O	3.45	3.40	3.44	3.44	3.35	3.39	3.40	3.40	3.41	3.37	3.33	3.39
P_2O_5	0.32	0.34	0.32	0.32	0.35	0.34	0.35	0.34	0.34	0.36	0.34	0.36
LOI	7.24	7.33	7.12	7.10	7.54	7.43	7.43	7.36	7.24	7.45	8.01	7.41
Sc	24.25	23.72	24.74	24.58	23.30	24.14	23.16	21.98	22.89	22.21	22.09	22.39
V	186.43	186.64	186.83	190.05	184.37	186.60	182.75	182.01	180.04	180.04	181.58	180.51
Cr	101.87	100.11	103.46	103.47	96.02	100.08	97.54	92.25	96.18	96.55	98.09	96.99
Co	89.51	92.90	91.80	90.91	94.10	94.24	94.64	92.60	90.82	92.41	93.60	93.73
Ni	155.89	154.24	155.54	149.39	153.91	153.00	153.19	159.92	157.00	150.18	157.06	156.89
Cu	256.18	270.30	263.21	257.23	268.36	269.88	271.05	265.74	260.49	260.90	268.06	264.99
Zn	151.54	153.20	154.19	155.67	157.62	160.74	157.46	156.02	158.72	153.80	154.55	154.11
As	26.08	25.58	28.15	28.00	27.45	26.75	27.97	25.07	24.78	25.96	25.35	26.67
Rb	136.29	133.05	135.94	137.58	131.62	133.79	131.30	131.77	133.42	127.53	134.52	130.97
Sr	184.20	190.70	186.06	188.45	187.79	191.50	188.17	188.82	183.27	189.87	191.24	187.62
Y	66.90	71.08	69.41	68.77	74.77	72.63	74.49	71.15	69.68	74.16	73.03	75.07
Zr	139.49	139.17	143.72	144.60	138.79	142.89	138.42	137.11	138.18	142.17	145.82	140.63
Nb	16.57	16.32	16.94	17.09	16.63	16.80	16.53	16.23	16.58	16.42	16.42	16.20
Mo	28.16	29.26	27.99	27.47	29.63	29.85	28.87	29.19	28.52	29.74	30.05	30.90
Cs	12.90	12.72	13.01	12.94	12.54	12.97	12.73	12.63	12.73	12.55	12.62	12.48
Ba	573.32	542.01	568.32	573.69	535.03	567.32	547.35	536.35	545.40	523.78	523.18	501.11
La	57.76	58.71	58.81	57.76	59.66	60.46	57.99	56.68	57.11	58.76	58.99	58.83
Ce	110.51	111.49	112.56	111.72	111.07	112.15	108.28	108.66	109.13	108.69	110.25	107.77
Pr	14.26	14.51	14.72	14.69	14.74	14.90	14.72	14.47	14.50	14.82	14.94	14.58
Nd	56.83	60.16	58.92	57.75	61.27	61.49	60.74	60.78	59.48	61.03	60.97	60.72
Sm	12.42	13.01	12.89	12.51	13.39	13.32	13.08	12.92	12.82	12.96	12.63	12.88
Eu	2.88	3.02	3.09	2.99	3.16	3.16	3.14	3.07	3.01	3.13	3.07	3.11
Gd	12.53	13.33	13.60	13.32	14.14	14.04	13.87	13.55	13.30	13.73	13.44	13.49
Tb	1.87	2.03	2.01	1.97	2.09	2.10	2.09	2.04	1.98	2.07	2.03	2.02
Dy	11.73	12.58	12.44	12.09	13.12	12.91	13.01	12.60	12.33	12.81	12.43	12.66
Ho	2.36	2.54	2.51	2.48	2.66	2.59	2.61	2.54	2.50	2.64	2.58	2.62
Er	6.93	7.39	7.38	7.21	7.69	7.51	7.51	7.32	7.26	7.64	7.41	7.51
Tm	0.98	1.06	1.04	1.03	1.08	1.08	1.06	1.03	1.02	1.06	1.05	1.07
Yb	6.39	6.83	6.79	6.50	7.07	6.93	6.85	6.67	6.53	6.95	6.89	6.96
Lu	0.99	1.04	1.03	1.01	1.07	1.06	1.06	1.03	1.03	1.07	1.07	1.08
Hf	3.02	3.01	3.06	3.02	3.05	3.05	3.04	2.97	3.04	3.11	3.12	3.03
Ta	0.94	0.92	0.94	0.93	0.90	0.90	0.90	0.91	0.91	0.91	0.91	0.88
Pb	34.30	36.30	34.57	33.84	35.45	35.28	35.79	36.14	34.91	35.54	35.35	35.29
Th	11.56	11.31	11.64	11.49	11.37	11.25	11.18	11.34	11.32	11.32	11.23	11.08
U	2.20	2.20	2.22	2.21	2.18	2.18	2.20	2.15	2.17	2.17	2.19	2.12
ΣREY	365.34	378.78	377.20	371.77	386.99	386.32	380.51	374.50	371.69	381.53	380.77	380.38

*, Total iron as Fe_2O_3; LOI, loss on ignition.

† Although compositions of these samples were reported by ref. 39, they were re-measured in this study along with the neighboring samples. Reproducibilities of replicate analyses were typically within 5%.

Table S2 (continued)

Element	PC11-4_58-60	PC11-4_60-62	PC11-4_62-64	PC11-4_64-66	PC11-4_66-68	PC11-4_68-70	PC11-4_70-72	PC11-4_72-74	PC11-4_74-76	PC11-4_84-86	PC11-4_94-96	PC11-5_4-6†
Depth	352.9	354.9	356.9	358.9	360.9	362.9	364.9	366.9	368.9	378.9	388.9	398.3
SiO_2	52.57	52.53	52.31	52.24	52.31	52.33	52.29	52.53	52.42	52.80	52.82	52.51
TiO_2	0.95	0.94	0.94	0.92	0.93	0.93	0.92	0.93	0.93	0.93	0.93	0.91
Al_2O_3	17.19	17.22	17.18	17.27	17.27	17.24	17.23	17.39	17.26	17.18	17.16	17.14
Fe_2O_3*	9.05	9.00	9.05	8.86	8.83	8.88	8.81	8.89	8.89	8.84	8.78	8.68
MnO	0.93	0.93	0.94	0.93	0.92	0.93	0.89	0.88	0.91	0.91	0.90	0.94
MgO	3.23	3.23	3.23	3.27	3.24	3.23	3.21	3.22	3.22	3.22	3.25	3.24
CaO	1.52	1.55	1.54	1.54	1.52	1.55	1.54	1.54	1.55	1.51	1.45	1.50
Na_2O	3.43	3.45	3.54	3.62	3.63	3.62	3.58	3.56	3.61	3.52	3.53	3.50
K_2O	3.38	3.35	3.35	3.35	3.36	3.35	3.32	3.33	3.34	3.36	3.42	3.33
P_2O_5	0.35	0.35	0.35	0.34	0.33	0.32	0.32	0.32	0.32	0.34	0.33	0.36
LOI	7.40	7.47	7.58	7.66	7.66	7.62	7.89	7.40	7.55	7.40	7.43	7.89
Sc	21.36	22.22	23.03	22.48	21.74	22.55	23.15	23.11	22.72	23.29	24.37	22.94
V	174.77	179.28	182.42	178.46	178.19	177.01	176.47	178.51	176.80	176.63	182.57	176.58
Cr	98.51	97.37	105.39	100.26	91.77	85.21	90.83	86.56	83.98	87.37	94.75	91.36
Co	90.97	91.71	94.91	91.81	88.87	90.62	88.46	88.01	93.35	90.86	94.39	96.67
Ni	153.14	155.87	159.70	156.99	158.80	156.81	154.46	146.75	154.28	152.68	159.94	168.70
Cu	262.02	264.47	272.21	262.66	261.88	261.80	265.69	260.27	266.84	263.80	271.44	276.15
Zn	151.54	152.48	155.88	146.94	148.96	149.11	154.06	154.34	160.29	160.22	169.07	161.68
As	25.70	26.12	25.76	24.55	22.51	23.49	23.35	23.75	24.20	24.52	25.08	26.13
Rb	129.36	127.05	124.93	121.70	118.03	116.44	120.33	120.13	121.84	123.68	130.82	128.73
Sr	186.07	185.13	191.48	185.89	185.93	182.25	189.51	185.63	191.85	181.62	184.08	179.99
Y	72.74	73.28	72.92	70.74	67.72	67.98	66.62	67.04	66.81	71.33	73.87	81.69
Zr	135.98	135.22	140.98	137.65	138.91	134.05	133.49	134.85	139.30	138.46	145.47	139.15
Nb	16.00	15.65	15.93	15.60	15.30	15.22	15.37	15.18	15.57	15.94	16.96	16.41
Mo	29.87	29.52	30.13	29.03	28.48	28.38	27.48	26.82	27.80	28.41	31.64	32.34
Cs	12.33	12.22	12.47	12.17	12.01	11.70	11.80	11.75	11.85	12.04	12.76	12.58
Ba	489.63	486.94	504.20	495.69	515.05	455.60	464.13	461.87	477.24	483.27	519.53	461.18
La	56.99	57.83	57.78	56.90	55.13	54.92	54.45	54.46	54.29	57.81	59.70	61.01
Ce	105.30	104.79	108.15	105.50	105.16	104.78	103.10	103.84	106.53	107.90	111.91	108.56
Pr	14.38	14.44	14.67	14.05	13.61	13.70	13.57	13.67	13.65	14.48	15.13	15.18
Nd	60.92	59.39	58.47	57.12	55.54	55.55	54.10	54.32	54.91	58.02	59.59	60.68
Sm	12.54	12.62	12.37	12.20	11.75	11.74	11.59	11.54	11.91	12.50	12.80	13.21
Eu	3.01	3.02	2.99	3.00	2.90	2.88	2.80	2.82	2.82	3.04	3.07	3.15
Gd	13.21	13.31	13.28	13.08	12.51	12.51	12.19	12.31	12.39	13.13	13.47	13.94
Tb	2.00	2.00	1.98	1.96	1.90	1.89	1.86	1.83	1.87	1.97	2.01	2.11
Dy	12.30	12.53	12.19	12.17	11.65	11.71	11.50	11.52	11.59	12.39	12.78	13.36
Ho	2.49	2.58	2.52	2.51	2.38	2.38	2.31	2.35	2.33	2.51	2.60	2.74
Er	7.27	7.34	7.33	7.29	6.92	6.91	6.70	6.76	6.84	7.18	7.49	7.93
Tm	1.05	1.05	1.03	1.03	0.98	0.97	0.96	0.96	0.97	1.03	1.04	1.12
Yb	6.75	6.78	6.64	6.59	6.31	6.30	6.10	6.13	6.17	6.74	6.83	7.26
Lu	1.07	1.06	1.06	1.03	0.98	0.99	0.95	0.97	0.97	1.05	1.06	1.12
Hf	3.04	3.00	3.10	3.11	3.10	3.01	2.99	2.91	3.02	3.00	3.07	3.02
Ta	0.89	0.88	0.90	0.89	0.88	0.87	0.86	0.85	0.86	0.88	0.91	0.91
Pb	35.30	35.86	35.39	35.03	33.91	33.33	33.65	32.67	34.15	33.45	33.80	34.57
Th	11.20	11.14	11.24	10.97	10.98	10.90	11.00	10.73	10.88	11.14	11.40	11.41
U	2.09	2.10	2.16	2.15	2.13	2.06	2.06	2.03	2.08	2.08	2.13	2.15
ΣREY	372.02	372.00	373.38	365.17	355.44	355.21	348.82	350.52	354.05	371.08	383.35	393.06

*, Total iron as Fe_2O_3; LOI, loss on ignition.

† Although compositions of these samples were reported by ref. 39, they were re-measured in this study along with the neighboring samples. Reproducibilities of replicate analyses were typically within 5%.

Table S2 (continued)

Element	PC11-5_14-16	PC11-5_24-26	PC11-5_54-56	PC11-6_4-6	PC11-6_54-56	PC11-7_4-6	PC11-7_54-56	PC11-8_4-6	PC11-8_54-56	PC11-9_4-6	PC11-9_54-56	PC11-10_4-6
Depth	408.3	418.3	448.3	498.4	548.4	599.5	649.5	699.6	749.6	798.8	848.8	898.7
SiO_2	52.69	51.08	50.64	50.70	50.08	50.37	50.37	46.64	45.33	47.29	46.58	46.35
TiO_2	0.92	0.89	0.93	0.90	0.83	0.78	0.70	0.78	0.77	0.73	0.70	0.67
Al_2O_3	17.15	17.65	16.72	16.52	16.70	16.66	16.54	14.82	14.02	14.22	13.73	13.40
Fe_2O_3*	8.80	9.11	9.11	8.92	8.28	7.95	7.27	7.51	7.33	7.04	6.72	6.51
MnO	0.97	1.12	1.00	1.07	1.25	1.28	1.46	1.82	1.95	1.98	1.98	1.96
MgO	3.22	2.97	3.28	3.12	2.79	2.59	2.41	2.57	2.67	2.80	2.72	2.67
CaO	1.59	1.69	1.52	1.98	2.14	1.93	2.26	5.45	6.75	5.69	6.59	7.45
Na_2O	3.49	3.74	3.80	4.04	4.43	4.76	4.72	4.19	3.99	3.68	3.67	3.58
K_2O	3.34	3.28	3.30	3.28	3.49	3.79	3.99	3.34	3.24	3.61	3.53	3.61
P_2O_5	0.39	0.38	0.45	0.60	0.77	0.70	1.02	3.21	4.23	3.56	4.22	4.84
LOI	7.45	8.08	9.24	8.87	9.23	9.19	9.25	9.67	9.72	9.40	9.57	8.97
Sc	25.22	23.66	24.33	25.26	28.43	26.73	27.54	48.03	53.04	49.00	55.84	59.84
V	183.86	170.02	168.48	149.63	148.00	146.71	119.69	133.65	120.78	119.46	115.27	106.04
Cr	95.26	61.06	70.17	50.93	44.35	41.87	35.42	36.92	33.23	33.16	33.14	35.77
Co	101.41	100.07	102.45	111.30	140.46	137.83	143.61	198.67	196.46	191.43	189.21	180.65
Ni	146.38	234.00	209.41	199.46	255.37	204.53	281.88	365.64	372.86	399.02	382.08	361.30
Cu	268.18	406.17	332.88	307.06	351.61	342.48	373.15	362.98	314.20	306.91	302.99	289.65
Zn	125.17	130.03	165.03	155.14	153.93	138.38	136.81	168.10	159.00	170.38	164.99	160.18
As	29.83	28.28	27.21	22.81	23.86	25.24	20.30	21.21	18.21	16.21	15.57	14.50
Rb	129.39	109.52	115.81	94.48	87.82	89.97	82.58	74.27	61.80	74.00	72.98	70.06
Sr	178.79	193.53	188.69	191.56	191.77	179.01	172.04	299.06	314.85	287.42	329.38	347.91
Y	92.21	83.04	98.73	123.51	152.01	131.20	182.21	610.76	722.21	604.15	767.51	871.93
Zr	141.08	160.43	150.31	136.85	139.27	130.98	132.75	146.75	139.16	143.35	143.88	137.54
Nb	15.84	15.18	15.96	13.38	11.63	11.53	11.05	13.19	12.84	12.95	12.32	11.39
Mo	31.95	31.69	32.28	37.63	47.37	51.07	49.95	65.08	62.97	63.12	62.19	56.97
Cs	12.55	10.01	12.50	9.69	7.19	6.96	5.72	5.74	4.50	5.30	5.07	4.73
Ba	423.27	398.82	361.79	316.88	286.32	271.61	248.53	275.41	258.87	309.25	322.79	310.05
La	65.20	59.61	63.79	71.69	79.73	73.60	99.67	309.48	361.37	306.52	387.08	438.30
Ce	108.42	102.53	96.62	89.48	94.98	93.30	104.25	196.25	213.17	209.46	240.13	250.21
Pr	16.57	15.78	18.03	20.69	23.41	21.47	30.67	92.74	107.11	89.83	114.99	138.71
Nd	69.22	66.39	69.16	81.22	93.69	85.16	122.40	374.76	432.91	362.48	464.24	522.65
Sm	14.80	14.83	16.07	19.43	22.97	20.51	30.14	93.08	107.42	89.76	115.09	129.08
Eu	3.14	3.23	3.97	4.92	5.85	5.17	7.44	23.18	26.63	22.22	28.46	32.10
Gd	15.51	15.48	17.61	22.39	26.68	23.84	34.55	106.17	122.91	102.64	131.32	147.44
Tb	2.31	2.30	2.71	3.41	4.11	3.62	5.19	16.01	18.53	15.50	19.78	22.23
Dy	15.00	14.51	17.14	21.79	26.05	22.84	32.36	99.56	115.65	96.90	123.47	138.54
Ho	3.06	2.91	3.60	4.55	5.51	4.79	6.69	20.41	23.93	20.04	25.49	28.76
Er	9.08	8.53	10.55	13.32	16.07	13.87	19.11	57.68	67.55	56.94	72.18	81.41
Tm	1.27	1.19	1.49	1.89	2.26	1.95	2.64	7.78	9.15	7.74	9.79	11.01
Yb	8.20	7.68	9.69	11.95	14.42	12.47	16.52	47.75	56.28	47.70	60.01	67.68
Lu	1.26	1.20	1.51	1.86	2.25	1.93	2.53	7.20	8.47	7.17	9.02	10.19
Hf	3.32	3.94	3.86	3.55	3.65	3.44	3.54	3.90	3.79	3.94	3.86	3.63
Ta	0.92	0.77	1.01	0.83	0.68	0.67	0.66	0.75	0.75	0.79	0.71	0.65
Pb	36.69	39.47	37.53	33.22	35.80	34.67	35.62	39.10	34.10	38.71	37.84	37.46
Th	12.17	11.29	12.79	11.33	10.35	9.67	12.16	20.03	20.25	18.42	21.72	23.21
U	2.32	2.04	2.60	2.41	2.47	2.34	2.25	4.28	4.51	4.02	4.46	4.62
ΣREY	425.26	399.22	430.67	492.11	570.02	515.75	696.37	2,062.81	2,393.30	2,039.04	2,568.55	2,890.23

*, Total iron as Fe_2O_3; LOI, loss on ignition.

Table S2 (continued)

Element	PC11-10_54-56	PC11-11_4-6	PC11-11_54-56	PC11-12_4-6	PC11-12_54-56	PC11-13_4-6	PC11-13_54-56
Depth	948.7	998.3	1,048.3	1,099.1	1,149.1	1,198.8	1,248.8
SiO_2	45.04	43.55	44.62	44.50	45.38	45.06	49.09
TiO_2	0.63	0.62	0.64	0.66	0.64	0.64	0.63
Al_2O_3	13.18	12.75	13.29	13.27	13.53	13.81	16.15
Fe_2O_3*	6.03	5.78	6.25	6.71	6.85	7.10	7.47
MnO	1.76	1.77	1.90	2.16	2.34	2.47	2.24
MgO	2.59	2.48	2.49	2.54	2.59	2.72	3.23
CaO	8.93	10.31	9.06	8.57	7.50	5.80	2.80
Na_2O	3.51	3.41	3.60	3.67	3.79	3.58	3.46
K_2O	3.51	3.49	3.53	3.44	3.60	3.56	3.91
P_2O_5	5.93	6.93	6.00	5.60	4.84	3.62	1.57
LOI	8.87	8.92	8.61	8.89	8.95	11.63	9.45
Sc	67.57	75.94	69.51	68.92	57.93	50.44	35.37
V	96.61	91.64	99.24	109.23	103.94	114.37	138.20
Cr	34.30	34.68	29.92	32.50	32.66	35.66	46.41
Co	155.84	151.15	153.22	180.14	169.71	197.38	198.21
Ni	399.16	423.01	364.59	379.73	400.71	398.37	441.98
Cu	288.42	277.16	267.84	284.01	277.70	282.58	274.52
Zn	160.83	156.45	151.06	160.13	156.57	159.14	176.13
As	11.99	10.36	13.63	16.73	16.84	17.38	17.61
Rb	69.03	67.71	67.33	68.66	68.27	83.22	123.28
Sr	401.36	454.25	395.25	410.60	342.59	301.28	198.69
Y	1,045.69	1,237.05	1,077.02	1,044.47	851.92	667.94	278.78
Zr	131.55	130.32	129.16	139.72	134.12	144.48	144.28
Nb	10.88	10.86	10.38	11.20	10.41	11.56	11.74
Mo	47.90	43.78	46.39	51.11	51.76	55.23	56.61
Cs	4.63	4.62	4.51	4.59	4.62	6.49	10.72
Ba	287.50	277.15	278.16	296.01	293.07	330.77	386.73
La	526.39	612.35	532.46	525.57	443.02	350.58	159.64
Ce	283.79	312.04	275.56	268.58	229.76	214.87	180.56
Pr	168.85	198.76	173.51	170.58	139.11	108.13	50.55
Nd	649.26	806.55	674.37	657.77	542.47	438.12	201.41
Sm	157.21	185.31	162.27	159.30	134.37	110.32	51.32
Eu	39.16	46.16	40.37	39.70	33.37	27.22	12.47
Gd	178.75	210.89	182.56	179.83	151.27	121.81	54.37
Tb	26.74	31.61	27.51	26.97	22.58	18.15	8.13
Dy	167.27	197.52	170.83	167.68	139.24	110.44	48.94
Ho	34.61	40.74	35.15	34.14	28.29	22.26	9.69
Er	97.97	115.06	99.26	96.10	79.36	61.73	26.99
Tm	13.25	15.55	13.29	12.87	10.55	8.20	3.61
Yb	81.27	95.06	80.91	78.04	63.53	49.25	22.13
Lu	12.17	14.26	12.12	11.66	9.43	7.25	3.31
Hf	3.54	3.49	3.35	3.58	3.41	3.73	3.73
Ta	0.62	0.54	0.57	0.60	0.57	0.64	0.74
Pb	35.21	34.07	35.91	41.62	41.31	45.40	53.76
Th	27.40	30.65	27.35	26.67	23.55	23.80	19.25
U	5.25	5.70	4.99	4.94	4.33	3.97	2.92
ΣREY	3,482.37	4,118.90	3,557.19	3,473.26	2,878.25	2,316.27	1,111.91

*, Total iron as Fe_2O_3; LOI, loss on ignition.

Table S3: PGE and Re concentrations and Re-Os and Os isotope ratios from core MR14-E02 PC11. See text for analytical procedures

Sample No.	Depth (cmbsf)	Weight (g)	$^{187}Re/^{188}Os$	2SE	$^{187}Os/^{188}Os$	2SE	Os (ppt)	2SE	Ir (ppt)	2SE
PC11-3_84-86	278.1	0.10485	8.4	0.7	0.85	0.06	120	9	298	26
PC11-3_94-96	288.1	0.10287	8.3	0.7	0.78	0.06	132	9	250	40
PC11-4_4-6	298.9	0.10714	7.5	0.5	0.69	0.04	155	9	350	30
PC11-4_14-16	308.9	0.10380	6.9	0.4	0.66	0.04	176	9	340	30
PC11-4_24-26	318.9	0.10207	3.99	0.22	0.480	0.028	235	9	470	30
PC11-4_34-36	328.9	0.10404	3.00	0.15	0.385	0.018	344	9	730	60
PC11-4_36-38	330.9	0.10296	1.77	0.07	0.274	0.011	645	9	1,240	100
PC11-4_38-40	332.9	0.10491	2.18	0.09	0.310	0.014	492	9	880	60
PC11-4_40-42	334.9	0.10369	3.13	0.14	0.384	0.018	361	9	770	50
PC11-4_42-44	336.9	0.09998	1.93	0.08	0.275	0.012	628	9	1,220	90
PC11-4_44-46	338.9	0.10267	1.91	0.08	0.284	0.012	590	10	1,220	80
PC11-4_46-48	340.9	0.10273	1.87	0.08	0.283	0.012	615	9	1,320	120
PC11-4_48-50	342.9	0.11288	1.90	0.07	0.276	0.011	609	8	1,130	90
PC11-4_50-52	344.9	0.11322	2.17	0.08	0.298	0.012	505	8	980	70
PC11-4_52-54	346.9	0.10423	1.35	0.05	0.231	0.009	877	9	1,410	190
PC11-4_54-56	348.9	0.10622	1.27	0.05	0.220	0.008	961	9	1,790	150
PC11-4_56-58	350.9	0.10422	1.34	0.05	0.224	0.008	935	9	1,850	160
PC11-4_58-60	352.9	0.10341	1.22	0.05	0.217	0.008	998	9	1,840	180
PC11-4_60-62	354.9	0.10334	1.26	0.05	0.247	0.008	973	9	1,930	170
PC11-4_62-64	356.9	0.10854	0.578	0.019	0.189	0.003	2,193	10	3,160	270
PC11-4_64-66	358.9	0.10315	1.37	0.06	0.227	0.009	848	9	1,610	120
PC11-4_66-68	360.9	0.10785	4.51	0.23	0.465	0.024	256	9	570	40
PC11-4_68-70	362.9	0.10553	7.5	0.5	0.703	0.043	160	9	380	30
PC11-4_70-72	364.9	0.11752	4.23	0.20	0.454	0.022	255	8	530	40
PC11-4_72-74	366.9	0.09936	7.6	0.5	0.71	0.04	165	10	370	30
PC11-4_74-76	368.9	0.10557	7.7	0.6	0.75	0.05	146	9	350	30
PC11-4_84-86	378.9	0.10762	6.4	0.4	0.68	0.04	168	9	340	30
PC11-4_94-96	388.9	0.10298	8.0	0.7	0.81	0.06	128	9	268	26
PC11-5_4-6	398.3	0.10677	8.3	0.7	0.82	0.05	131	9	296	23

All data are blank corrected with error propagation. Errors are determined on the basis of results of ref. 47.

Table S3 (continued)

Sample No.	Depth (cmbsf)	Ru (ppt)	2SE	Pt (ppt)	2SE	Pd (ppt)	2SE	Re (ppt)	2SE
PC11-3_84-86	278.1	540	40	8,100	400	3,410	100	191	7
PC11-3_94-96	288.1	570	40	7,900	300	3,390	130	210	8
PC11-4_4-6	298.9	670	40	8,700	400	3,300	120	225	8
PC11-4_14-16	308.9	710	50	8,700	400	3,430	110	237	8
PC11-4_24-26	318.9	780	40	8,800	300	3,350	100	187	7
PC11-4_34-36	328.9	980	70	9,200	400	3,770	120	207	8
PC11-4_36-38	330.9	1,660	70	10,400	400	3,950	100	232	9
PC11-4_38-40	332.9	1,180	50	10,100	400	3,860	120	217	9
PC11-4_40-42	334.9	1,020	50	9,400	400	4,000	110	227	8
PC11-4_42-44	336.9	1,580	70	10,200	500	3,770	130	247	9
PC11-4_44-46	338.9	1,520	60	10,300	400	3,850	100	230	8
PC11-4_46-48	340.9	1,580	80	10,600	500	4,120	100	234	10
PC11-4_48-50	342.9	1,490	60	10,100	300	4,070	130	235	9
PC11-4_50-52	344.9	1,230	50	9,200	300	3,800	110	223	8
PC11-4_52-54	346.9	2,040	100	11,200	600	4,280	140	243	9
PC11-4_54-56	348.9	1,990	80	11,500	500	4,030	130	250	9
PC11-4_56-58	350.9	2,030	80	12,000	400	4,100	110	256	9
PC11-4_58-60	352.9	2,040	100	12,200	500	4,090	140	250	10
PC11-4_60-62	354.9	2,300	90	11,600	400	4,010	130	250	9
PC11-4_62-64	356.9	3,050	100	13,400	600	4,240	140	261	9
PC11-4_64-66	358.9	1,890	90	11,100	500	3,930	120	238	9
PC11-4_66-68	360.9	1,120	50	8,300	300	3,780	150	229	9
PC11-4_68-70	362.9	930	40	8,000	300	3,600	130	230	9
PC11-4_70-72	364.9	1,010	50	8,300	300	3,580	120	215	8
PC11-4_72-74	366.9	850	40	8,300	300	4,030	150	241	9
PC11-4_74-76	368.9	710	50	7,720	260	3,490	150	215	9
PC11-4_84-86	378.9	730	40	8,800	300	3,800	160	207	7
PC11-4_94-96	388.9	600	30	7,950	280	3,610	120	195	8
PC11-5_4-6	398.3	620	40	8,600	400	3,830	140	206	9

All data are blank corrected with error propagation. Errors are determined on the basis of results of ref. 47.

Table S4: Spinel, matrix and representative mineral phase compositions of piston core samples determined by EPMA

Analytical No.	SiO_2 (wt%)	TiO_2 (wt%)	Al_2O_3 (wt%)	Cr_2O_3 (wt%)	FeO* (wt%)	MnO (wt%)	NiO (wt%)	MgO (wt%)	CaO (wt%)	K_2O (wt%)	Na_2O (wt%)	P_2O_5 (wt%)	Total (wt%)	Comment and sample no.	Target mineral
1	68.375	0.085	19.954	0.033	0	0	0	0	0.359	0.238	11.747	0	100.791	Albite standard	Albite
2	56.266	0.129	2.527	0.374	5.475	0.100	0.020	34.355	0.572	0	0.103	0	99.921	Enstatite standard	Enstatite
3	39.395	0.021	22.06	0	23.421	0.649	0.068	10.755	4.085	0	0	0.010	100.464	Almandine standard	Almandine
4	0.371	1.102	8.447	7.470	71.126	0.158	1.348	2.263	0	0	0	0	92.285	PC11-4_56-58-1-1	Euhedral spinel
5	0.405	1.164	8.765	4.392	75.410	0.077	1.237	2.011	0	0	0	0.058	93.519	PC11-4_56-58-1-2	Euhedral spinel
6	0.270	0.485	8.324	35.325	41.747	0.237	1.857	6.299	0.028	0.012	0.024	0.030	94.638	PC11-4_56-58-1-3	Euhedral spinel
7	0.524	1.240	8.278	5.792	73.527	0.158	1.198	2.009	0	0.020	0.035	0.002	92.783	PC11-4_56-58-1-4	Euhedral spinel
8	0.506	1.062	8.929	6.347	72.416	0.145	1.164	2.028	0	0.015	0	0.021	92.633	PC11-4_56-58-1-5	Euhedral spinel
9	1.184	0.666	0.694	0.150	82.747	0.173	0.494	0.579	0	0.049	0	0.057	86.793	PC11-4_56-58-1-6	Dendritic spinel
10	0.633	0.632	0.788	0.264	85.040	0.199	0.738	0.788	0	0	0	0	89.082	PC11-4_56-58-1-7	Dendritic spinel
11	0.802	0.429	0.460	0.241	92.412	0.139	0.500	0.440	0	0.032	0	0.014	95.469	PC11-4_56-58-1-8	Dendritic spinel
12	0.647	0.612	0.351	0.170	86.975	0.123	0.438	0.193	0	0	0	0.007	89.516	PC11-4_56-58-1-9	Dendritic spinel
13	0.804	0.705	0.628	0.236	85.627	0.166	0.545	0.437	0	0.039	0.053	0	89.240	PC11-4_56-58-1-10	Dendritic spinel
14	0.556	0.753	0.530	0.182	84.980	0.146	0.463	0.383	0	0	0.097	0.033	88.123	PC11-4_56-58-1-11	Dendritic spinel
15	18.211	2.014	4.074	0.202	55.966	0	0	1.707	0.513	0.727	0.324	1.314	85.052	PC11-4_56-58-1-12	Matrix
16	10.335	2.477	3.407	0.150	59.064	0.003	0	1.402	0.628	0.068	0.193	1.543	79.270	PC11-4_56-58-1-13	Matrix
17	18.386	2.089	3.539	0.094	55.863	0	0.019	1.634	0.601	0.827	0.221	1.260	84.533	PC11-4_56-58-1-14	Matrix
18	17.077	2.179	3.283	0.268	56.928	0.017	0	1.587	0.580	0.613	0.321	1.290	84.143	PC11-4_56-58-1-15	Matrix
19	53.954	0.405	2.231	0	8.985	0.514	0.042	13.565	20.946	0.024	0.242	0	100.908	PC11-4_56-58-2-1	Clinopyroxene
20	0.944	1.191	6.884	7.117	70.261	0.175	0.979	2.639	0.013	0.062	0	0.105	90.370	PC11-4_56-58-2-2	Euhedral spinel
21	0.262	0.647	8.215	30.691	46.354	0.247	1.160	5.056	0	0	0.018	0	92.650	PC11-4_56-58-2-3	Euhedral spinel
22	0.487	1.046	7.960	8.095	70.224	0.161	0.945	2.273	0.010	0	0	0.015	91.216	PC11-4_56-58-2-4	Euhedral spinel
23	0.542	1.359	5.607	0.797	73.428	0.057	0.465	1.328	0	0.050	0.032	0.055	83.720	PC11-4_56-58-2-5	Euhedral spinel
24	0.442	0.990	7.677	15.729	60.236	0.260	0.781	1.976	0	0.005	0	0	88.096	PC11-4_56-58-2-6	Euhedral spinel
25	0.521	0.889	5.471	3.864	74.803	0.198	0.696	2.004	0	0.015	0	0.021	88.482	PC11-4_56-58-2-7	Euhedral spinel
26	0.278	0.653	6.950	24.384	53.473	0.294	0.964	4.912	0	0.017	0.028	0.026	91.979	PC11-4_56-58-2-8	Euhedral spinel
27	0.245	0.558	6.960	29.189	46.180	0.237	1.230	5.494	0.003	0.025	0	0	90.121	PC11-4_56-58-2-9	Euhedral spinel
28	0.483	1.335	6.205	0.775	75.307	0.151	0.656	1.458	0	0.032	0	0	86.402	PC11-4_56-58-2-10	Euhedral spinel
29	7.820	2.649	1.069	0.210	37.653	0.281	0.087	1.289	0.511	0.020	0.125	0.995	52.709	PC11-4_56-58-2-11	Matrix
30	7.403	2.461	0.796	0.092	42.505	0.345	0.096	1.606	0.402	0.028	0.182	0.870	56.786	PC11-4_56-58-2-12	Matrix
31	10.78	3.101	1.049	0.050	46.138	0.408	0.103	1.706	0.643	0.018	0.185	1.108	65.289	PC11-4_56-58-2-13	Matrix
32	6.301	2.186	1.418	0.112	54.056	0.064	0	1.321	0.400	0.073	0.154	0.767	66.852	PC11-4_56-58-2-14	Matrix
33	7.588	2.981	2.014	0.062	48.086	0.287	0.071	1.390	0.480	0.025	0.163	1.099	64.246	PC11-4_56-58-2-15	Matrix
34	7.808	2.596	2.289	0.175	44.325	0.188	0.068	1.503	0.526	0.030	0.116	1.129	60.753	PC11-4_56-58-2-16	Matrix
35	0.554	1.506	6.375	0.514	77.668	0.107	0.672	1.415	0	0	0	0	88.811	PC11-4_56-58-4-1	Euhedral spinel
36	0.564	1.120	7.049	2.602	75.525	0.155	1.135	1.844	0	0.025	0	0	90.019	PC11-4_56-58-4-2	Euhedral spinel
37	0.268	0.645	7.646	25.392	47.084	0.152	1.243	2.782	0	0.002	0.009	0.023	85.246	PC11-4_56-58-4-3	Euhedral spinel
38	0.481	1.198	7.009	1.787	75.307	0.094	0.960	2.161	0	0	0.013	0	89.010	PC11-4_56-58-4-4	Euhedral spinel
39	0.230	0.577	7.638	22.053	47.743	0.139	1.432	4.544	0	0	0.015	0	84.371	PC11-4_56-58-4-5	Euhedral spinel
40	0.662	1.350	7.081	0.328	75.785	0.241	0.689	1.036	0	0	0	0.020	87.192	PC11-4_56-58-4-6	Euhedral spinel
41	6.161	1.212	1.489	0.135	41.545	0.034	0.015	0.738	0.747	0.040	0.227	0.546	52.889	PC11-4_56-58-4-7	Matrix
42	6.936	1.930	2.262	0.147	45.177	0.146	0.087	1.095	0.584	0.030	0.268	0.975	59.637	PC11-4_56-58-4-8	Matrix
43	7.179	1.994	2.158	0.075	47.298	0.116	0.019	0.985	0.651	0.030	0.186	0.900	61.591	PC11-4_56-58-4-9	Matrix
44	7.252	1.873	1.983	0.118	41.295	0.174	0	0.973	0.567	0.023	0.255	0.823	55.336	PC11-4_56-58-4-10	Matrix
45	5.862	1.957	1.872	0.174	44.381	0.146	0	0.869	0.488	0.035	0.171	0.679	56.634	PC11-4_56-58-4-11	Matrix
46	6.480	2.064	2.412	0.255	43.394	0.163	0.139	1.104	0.474	0.088	0.166	0.859	57.598	PC11-4_56-58-4-12	Matrix
47	0.202	0.405	7.303	40.851	34.415	0.241	1.172	8.282	0	0	0.006	0.019	92.896	PC11-4_56-58-8-1	Euhedral spinel
48	0.277	0.607	7.251	36.944	39.432	0.267	1.437	7.422	0	0.010	0.003	0.058	93.708	PC11-4_56-58-8-2	Euhedral spinel
49	0.743	1.263	8.974	9.632	64.786	0.175	0.611	2.494	0.049	0.045	0.103	0.028	88.903	PC11-4_56-58-8-3	Euhedral spinel
50	0.551	1.042	8.676	12.539	59.606	0.219	0.685	2.716	0.015	0.002	0.012	0	86.063	PC11-4_56-58-8-4	Euhedral spinel
51	0.553	1.421	8.546	4.049	73.132	0.252	0.444	2.000	0	0	0.032	0	90.429	PC11-4_56-58-8-5	Euhedral spinel
52	0.730	1.822	9.015	7.410	71.159	0.128	0.340	1.037	0	0.007	0.028	0	91.676	PC11-4_56-58-8-6	Euhedral spinel
53	12.152	2.175	1.475	0.120	44.359	0.274	0.034	1.537	0.584	0.097	0.18	1.032	64.019	PC11-4_56-58-8-7	Matrix
54	6.384	1.683	2.371	0.148	35.592	0.089	0	1.269	0.353	0.232	0.097	0.905	49.123	PC11-4_56-58-8-8	Matrix
55	6.745	1.494	1.122	0.168	31.037	0.099	0.006	0.994	0.322	0.099	0.1	0.697	42.883	PC11-4_56-58-8-9	Matrix
56	21.646	1.688	3.575	0.195	41.891	0.028	0	2.070	0.515	1.346	0.214	1.009	74.177	PC11-4_56-58-8-10	Matrix
57	17.821	2.472	2.756	0.159	52.813	0.165	0.106	1.882	0.639	0.753	0.162	1.022	80.750	PC11-4_56-58-8-11	Matrix
58	13.112	2.031	6.093	0.070	57.763	0.034	0.043	1.564	0.148	0.935	0.212	0.764	82.769	PC11-4_56-58-8-12	Matrix
59	0.324	0.633	7.431	30.899	46.149	0.304	1.047	5.754	0	0.012	0	0.023	92.576	PC11-4_58-60-4-1	Euhedral spinel
60	0.510	1.649	6.315	0.636	78.331	0.144	0.821	1.739	0	0	0	0	90.145	PC11-4_58-60-4-2	Euhedral spinel
61	0.326	1.379	8.332	8.194	68.747	0.218	0.838	2.073	0	0	0	0.040	90.147	PC11-4_58-60-4-3	Dendritic spinel
62	0.481	1.456	6.458	3.296	76.469	0.097	0.806	1.864	0	0	0	0.038	90.965	PC11-4_58-60-4-4	Euhedral spinel
63	0.493	0.755	0.625	0.197	85.103	0.258	0.316	0.790	0	0.022	0.017	0	88.576	PC11-4_58-60-4-5	Dendritic spinel
64	0.492	0.610	0.578	0.196	91.391	0.199	0.379	0.412	0	0.049	0.066	0	94.372	PC11-4_58-60-4-6	Dendritic spinel
65	0.282	0.553	8.566	34.843	41.071	0.254	1.261	7.529	0	0	0.012	0	94.371	PC11-4_58-60-4-7	Euhedral spinel
66	0.513	1.423	8.451	5.791	69.370	0.124	0.811	1.646	0	0	0.013	0.029	88.171	PC11-4_58-60-4-8	Euhedral spinel
67	0.333	0.875	7.582	19.812	56.121	0.252	0.929	4.484	0.005	0	0.095	0	90.488	PC11-4_58-60-4-9	Dendritic spinel
68	0.255	0.662	8.139	31.182	45.946	0.189	1.139	6.129	0	0.015	0.08	0	93.736	PC11-4_58-60-4-10	Euhedral spinel
69	8.200	3.439	3.790	0.222	59.080	0.003	0	1.510	0.499	0.063	0.248	1.753	78.807	PC11-4_58-60-4-11	Matrix
70	8.866	2.888	3.143	0.316	58.500	0.068	0	1.475	0.551	0.065	0.175	1.449	77.496	PC11-4_58-60-4-12	Matrix
71	8.649	2.582	2.962	0.240	56.992	0.048	0	1.441	0.564	0.110	0.236	1.513	75.337	PC11-4_58-60-4-13	Matrix
72	9.008	2.605	3.410	0.283	57.758	0.034	0	1.633	0.586	0.083	0.241	1.668	77.309	PC11-4_58-60-4-14	Matrix
73	7.886	1.847	1.534	0.135	36.649	0.136	0.062	1.463	0.419	0.038	0.187	0.939	51.295	PC11-4_58-60-4-15	Matrix
74	0.081	51.114	0.119	0.052	42.279	0.922	0	0.698	0.039	0.038	0.058	0	95.400	PC11-4_58-60-7-1	Ilmenite
75	33.283	1.845	16.995	0	20.029	0.277	0.093	10.006	0.110	4.874	0.279	0.019	87.810	PC11-4_58-60-7-2	Illite
76	33.785	1.563	17.306	0.008	19.736	0.285	0.013	9.985	0.184	4.886	0.322	0	88.073	PC11-4_58-60-7-3	Illite

FeO*, total iron as FeO. Grey shaded columns are not extraterrestrial materials.

Table S4 (continued)

Analytical No.	SiO_2 (wt%)	TiO_2 (wt%)	Al_2O_3 (wt%)	Cr_2O_3 (wt%)	FeO* (wt%)	MnO (wt%)	NiO (wt%)	MgO (wt%)	CaO (wt%)	K_2O (wt%)	Na_2O (wt%)	P_2O_5 (wt%)	Total (wt%)	Comment and sample no.	Target mineral
77	35.662	1.674	16.409	0.097	19.087	0.151	0.042	5.228	0.074	4.742	0.116	0	83.282	PC11-4_58-60-7-4	Illite
78	46.420	0.040	39.657	0	0.706	0	0.036	0.024	0.397	1.204	4.274	0	92.758	PC11-4_58-60-7-5	Zeolite (?)
79	48.188	0.055	39.103	0.062	0.672	0	0.003	0.074	0.328	1.660	4.038	0	94.183	PC11-4_58-60-7-6	Zeolite (?)
80	49.401	0.088	40.967	0.017	0.537	0.044	0.049	0.038	0.403	1.167	4.03	0	96.741	PC11-4_58-60-7-7	Zeolite (?)
81	94.312	0.063	0.766	0.062	1.161	0	0.010	0.198	0.023	0.118	0	0	96.713	PC11-4_58-60-7-8	Quartz
82	96.714	0.019	0.382	0	0.646	0	0	0	0.070	0.023	0.008	0	97.862	PC11-4_58-60-7-9	Quartz
83	34.964	1.061	13.624	0.008	14.857	0.326	0	8.015	0.199	4.270	0.137	0.008	77.469	PC11-4_58-60-7-10	Illite
84	53.848	0.139	27.398	0.025	4.451	0.040	0.006	3.322	0.291	4.605	0.208	0	94.333	PC11-4_58-60-7-11	Zeolite (?) and illite
85	41.669	0.349	24.63	0.041	2.812	0	0.065	1.521	0.210	5.111	0.279	0	76.687	PC11-4_58-60-7-12	Illite
86	54.288	0	31.052	0.058	1.033	0.015	0.036	0.125	12.687	0.215	3.494	0	103.003	PC11-4_58-60-7-13	Zeolite (?)
87	89.350	0.019	0.600	0.025	0.417	0.004	0.029	0.060	0.006	0.059	0.047	0	90.616	PC11-4_58-60-7-14	Quartz
88	90.667	0.100	3.000	0.133	1.559	0.015	0.062	0.480	0.085	0.470	0.024	0	96.595	PC11-4_58-60-7-15	Quartz
89	28.644	0.073	20.853	0.048	28.107	0.075	0.025	11.610	0	0.067	0.005	0	89.507	PC11-4_58-60-7-16	Chlorite/smectite
90	98.455	0	0.154	0.050	0.903	0.004	0.059	0.009	0.029	0	0.026	0	99.689	PC11-4_58-60-7-17	Chlorite/smectite
91	15.700	3.929	6.854	0.197	55.630	0.158	0.062	2.379	0.843	0.520	0.154	1.778	88.204	PC11-4_58-60-7-18	Matrix
102	0.128	2.463	0.894	0.027	83.212	0.618	0.027	1.167	0.068	0	0.023	0.030	88.657	PC11-4_58-60-11-1	Magnetite
103	0.363	2.486	0.833	0.067	82.190	0.704	0.052	1.058	0.190	0.076	0.02	0.016	88.055	PC11-4_58-60-11-2	Magnetite
104	1.195	2.632	1.243	0.119	80.014	0.739	0.043	1.090	0.147	0.265	0	0	87.487	PC11-4_58-60-11-3	Magnetite
105	0.080	2.344	0.825	0.097	82.384	0.799	0.052	1.059	0.162	0.015	0	0.063	87.880	PC11-4_58-60-11-4	Magnetite
106	49.711	0.026	30.565	0.004	1.616	0.033	0	0.048	13.774	0.160	3.325	0	99.262	PC11-4_58-60-11-5	Zeolite (?)
107	50.169	0.126	28.332	0.033	1.484	0.055	0.052	0.130	11.799	0.163	3.932	0	96.275	PC11-4_58-60-11-6	Zeolite (?)
108	51.589	0	30.08	0	1.702	0	0.032	0.005	12.968	0.133	3.051	0	99.560	PC11-4_58-60-11-7	Zeolite (?)
109	49.314	0.115	30.389	0	1.588	0	0	0.022	13.970	0.162	3.202	0	98.762	PC11-4_58-60-11-8	Zeolite (?)
110	0.527	1.893	13.766	0.772	72.915	0.215	0.834	0.771	0	0	0	0	91.693	PC11-4_60-62-7-1	Euhedral spinel
111	0.566	2.113	9.910	0.228	73.790	0.107	0.309	0.386	0	0.030	0	0.014	87.453	PC11-4_60-62-7-2	Euhedral spinel
112	0.423	1.815	11.084	1.659	74.689	0.259	0.995	1.454	0	0	0.028	0	92.406	PC11-4_60-62-7-3	Euhedral spinel
113	0.488	1.538	10.499	0.553	71.019	0.221	0.523	0.500	0.028	0	0.041	0	85.410	PC11-4_60-62-7-4	Euhedral spinel
114	0.521	1.596	11.647	0.890	74.136	0.198	1.059	1.226	0	0.030	0.019	0	91.322	PC11-4_60-62-7-5	Euhedral spinel
115	0.693	1.466	9.424	0.179	75.579	0.241	0.312	0.399	0	0.005	0.032	0	88.330	PC11-4_60-62-7-6	Euhedral spinel
116	0.655	1.504	12.624	0.411	61.384	0.259	0.595	0.207	0	0.010	0.015	0	77.664	PC11-4_60-62-7-7	Euhedral spinel
117	3.073	1.541	0.731	0.006	37.345	0.034	0.076	0.112	0.318	0.131	0.045	0.472	43.884	PC11-4_60-62-7-8	Dendritic spinel
118	0.615	0.531	1.451	0.152	81.323	0.112	0.246	0.126	0	0	0	0	84.556	PC11-4_60-62-7-9	Dendritic spinel
119	0.487	1.162	7.691	0.278	43.015	0.077	0.273	0.514	0	0	0.068	0.058	53.623	PC11-4_60-62-7-10	Dendritic spinel
120	1.388	0.769	4.703	0.159	36.812	0.322	0.015	0.054	0	0.072	0.009	0.032	44.335	PC11-4_60-62-7-11	Euhedral spinel
121	15.599	1.880	1.104	0.080	47.459	0.134	0.121	1.647	0.622	0.122	0.244	1.007	70.019	PC11-4_60-62-7-12	Matrix
122	14.388	2.414	4.000	0.127	51.983	0.079	0	1.460	0.454	0.777	0.246	1.017	76.945	PC11-4_60-62-7-13	Matrix
123	16.773	2.117	1.433	0.137	50.813	0.137	0.211	1.899	0.660	0.361	0.231	1.019	75.791	PC11-4_60-62-7-14	Matrix
124	0.453	1.043	5.898	3.482	73.579	0.174	1.642	2.949	0	0	0	0.059	89.279	PC11-4_60-62-10-1	Euhedral spinel
125	0.347	0.972	5.444	4.834	73.482	0.190	1.459	3.708	0	0.002	0	0	90.438	PC11-4_60-62-10-2	Euhedral spinel
126	0.397	1.019	5.217	0.458	76.380	0.183	0.834	1.056	0	0.007	0	0	85.551	PC11-4_60-62-10-3	Euhedral spinel
127	0.301	0.666	5.664	10.866	65.650	0.274	1.203	2.786	0	0.040	0.054	0	87.504	PC11-4_60-62-10-4	Euhedral spinel
128	0.534	0.917	4.997	1.089	76.927	0.150	0.633	0.958	0	0	0	0.019	86.224	PC11-4_60-62-10-5	Euhedral spinel
129	0.510	0.591	5.240	19.473	57.477	0.218	1.407	4.417	0	0.022	0.068	0	89.423	PC11-4_60-62-10-6	Euhedral spinel
130	3.018	0.970	0.804	0.064	61.497	0.107	0.027	0.546	0.099	0.015	0	0.328	67.475	PC11-4_60-62-10-7	Dendritic spinel
131	0.294	0.694	5.859	20.829	56.008	0.309	1.212	5.171	0	0.020	0	0.044	90.440	PC11-4_60-62-10-8	Euhedral/dendritic spinel
132	1.612	0.713	6.798	14.272	56.010	0.191	1.065	2.035	0.187	0.027	0.201	0.024	83.135	PC11-4_60-62-10-9	Euhedral/dendritic spinel
133	1.004	0.939	5.682	4.505	72.300	0.167	0.805	1.976	0.003	0	0.067	0.070	87.518	PC11-4_60-62-10-10	Euhedral spinel
134	37.115	0.178	14.031	0	13.418	0.282	0.029	4.232	0.247	1.436	0.318	0.021	71.307	PC11-4_60-62-10-11	Chlorite/smectite
135	41.493	0.585	22.531	0.097	7.115	0.066	0	2.297	0.156	4.055	0.189	0.037	78.621	PC11-4_60-62-10-12	Illite
136	3.686	1.666	2.777	0.185	57.091	0.386	0.226	1.229	0.213	0	0.159	0.378	67.996	PC11-4_60-62-10-13	Matrix
137	5.656	2.713	2.722	1.143	46.291	0.436	0.230	1.371	0.398	0.017	0.179	0.745	61.901	PC11-4_60-62-10-14	Matrix
138	0.237	0.383	7.352	41.515	34.555	0.182	1.359	8.612	0	0.012	0	0.001	94.208	PC11-4_60-62-10-15	Euhedral spinel
139	0.246	0.416	6.992	41.800	34.961	0.313	1.351	8.255	0.023	0.059	0	0	94.416	PC11-4_60-62-10-16	Euhedral spinel
140	0.323	1.181	8.259	11.760	64.270	0.214	0.791	2.397	0	0	0	0	89.195	PC11-4_60-62-10-17	Euhedral spinel
141	0.383	1.081	10.485	13.425	60.196	0.264	1.320	2.959	0	0	0	0	90.113	PC11-4_60-62-10-18	Euhedral spinel
142	0.427	1.828	5.798	1.430	74.566	0.149	0.662	1.297	0	0.015	0.019	0	86.191	PC11-4_60-62-10-19	Euhedral spinel
143	0.912	1.355	7.530	4.968	71.635	0.207	0.961	1.928	0.031	0.039	0.17	0	89.736	PC11-4_60-62-10-20	Euhedral spinel
144	0.479	1.978	3.728	0.298	74.869	0.156	0.185	0.506	0	0.010	0.103	0.012	82.324	PC11-4_60-62-10-21	Euhedral spinel
145	0.919	2.132	6.709	0.200	76.287	0.120	0.389	0.503	0	0.044	0.064	0.032	87.399	PC11-4_60-62-10-22	Euhedral spinel
146	0.512	1.589	8.291	4.694	73.743	0.113	0.896	1.375	0	0	0	0	91.213	PC11-4_60-62-10-23	Euhedral spinel
147	0.472	0.267	0.456	0.177	84.586	0.187	0.160	0.374	0	0.034	0.013	0	86.726	PC11-4_60-62-10-24	Dendritic spinel
148	0.643	0.668	0.483	0.183	82.029	0.181	0.042	0.418	0	0.022	0	0.002	84.671	PC11-4_60-62-10-25	Dendritic spinel
149	0.706	0.551	1.654	0.312	85.627	0.122	0.338	0.490	0.048	0.037	0.075	0	89.960	PC11-4_60-62-10-26	Euhedral spinel
150	1.024	1.017	2.640	0.370	78.441	0.228	0.266	0.342	0.015	0.068	0.052	0.059	84.522	PC11-4_60-62-10-27	Dendritic spinel
151	1.093	0.585	1.711	0.199	81.908	0.178	0.248	0.694	0	0.010	0	0.075	86.701	PC11-4_60-62-10-28	Dendritic spinel
152	0.579	2.081	8.890	0.195	76.710	0.203	0.377	0.692	0	0.032	0.041	0	89.800	PC11-4_60-62-10-29	Euhedral spinel
153	0.522	1.936	7.907	0.413	77.063	0.239	0.756	0.882	0	0.049	0	0	89.767	PC11-4_60-62-10-30	Euhedral spinel
154	0.576	0.302	0.672	0.128	83.693	0.207	4.776	0.449	0	0.007	0	0.047	90.857	PC11-4_60-62-10-31	Euhedral/dendritic spinel
155	1.640	0.902	3.864	0.088	61.640	0.139	0.085	0.412	0.018	0.098	0.006	0	68.892	PC11-4_60-62-10-32	Euhedral/dendritic spinel
156	0.614	1.673	8.545	0.163	76.410	0.213	0.194	0.521	0	0.025	0	0.005	88.363	PC11-4_60-62-10-33	Euhedral/dendritic spinel
157	1.536	0.650	1.791	0.152	73.982	0.439	0.097	0.409	0.028	0.002	0.026	0.107	79.219	PC11-4_60-62-10-34	Euhedral/dendritic spinel
158	0.775	1.805	8.330	0.160	76.115	0.100	0.237	0.400	0	0	0	0	87.922	PC11-4_60-62-10-35	Euhedral spinel
159	0.779	0.422	0.675	0.224	85.072	0.184	0.148	0.386	0.043	0.005	0	0	87.938	PC11-4_60-62-10-36	Dendritic spinel
160	0.975	1.124	11.028	14.163	52.852	0.189	1.073	2.956	0	0.010	0.048	0.113	84.531	PC11-4_60-62-10-37	Euhedral spinel
161	0.807	1.905	8.543	0.358	73.955	0.171	0.349	0.887	0.021	0.032	0.003	0.052	87.083	PC11-4_60-62-10-38	Euhedral spinel
162	1.051	1.731	9.488	0.605	71.399	0.248	0.462	0.733	0.023	0.037	0.028	0.075	85.880	PC11-4_60-62-10-39	Euhedral spinel

FeO*, total iron as FeO. Grey shaded columns are not extraterrestrial materials.

Table S4 (continued)

Analytical No.	SiO_2 (wt%)	TiO_2 (wt%)	Al_2O_3 (wt%)	Cr_2O_3 (wt%)	FeO* (wt%)	MnO (wt%)	NiO (wt%)	MgO (wt%)	CaO (wt%)	K_2O (wt%)	Na_2O (wt%)	P_2O_5 (wt%)	Total (wt%)	Comment and sample no.	Target mineral
163	0.667	0.960	3.872	0.152	81.876	0.167	0.352	0.487	0	0	0.02	0.038	88.591	PC11-4_60-62-10-40	Euhedral/dendritic spinel
164	11.234	3.795	1.183	0.068	38.206	0.864	0.087	1.395	0.745	0	0.197	1.068	58.842	PC11-4_60-62-10-41	Matrix
165	11.126	3.825	1.121	0.167	38.942	0.739	0.044	1.735	0.712	0.025	0.218	1.089	59.743	PC11-4_60-62-10-42	Matrix
166	9.400	3.550	1.327	0.187	36.032	0.483	0.143	1.453	0.530	0.058	0.24	1.072	54.475	PC11-4_60-62-10-43	Matrix
167	9.994	3.730	1.169	0.089	36.401	0.631	0.115	1.460	0.622	0	0.155	1.157	55.523	PC11-4_60-62-10-44	Matrix
168	12.567	3.726	1.151	0.068	44.486	0.872	0.016	1.640	0.820	0.048	0.171	1.048	66.613	PC11-4_60-62-10-45	Matrix
169	11.211	3.371	0.927	0.054	40.983	0.709	0.125	1.649	0.679	0.025	0.267	0.848	60.848	PC11-4_60-62-10-46	Matrix
170	8.162	3.615	1.520	0.147	41.350	0.483	0.081	1.107	0.625	0.061	0.171	1.102	58.424	PC11-4_60-62-10-47	Matrix
171	9.853	3.291	1.124	0.223	37.712	0.753	0.118	1.700	0.655	0.013	0.126	0.950	56.518	PC11-4_60-62-10-48	Matrix
172	10.047	3.548	1.391	0.058	37.328	0.644	0.121	1.606	0.637	0.033	0.23	1.025	56.668	PC11-4_60-62-10-49	Matrix
173	10.114	3.594	1.567	0.092	35.545	0.699	0.156	1.286	0.603	0.074	0.205	0.923	54.858	PC11-4_60-62-10-50	Matrix
174	0.481	0.821	4.428	1.588	75.993	0.096	0.887	2.128	0.008	0	0	0	86.430	PC11-4_62-64-5-1	Euhedral spinel
175	1.130	0.726	3.879	0.232	74.529	0.235	0.345	1.037	0	0	0.019	0.039	82.171	PC11-4_62-64-5-2	Euhedral spinel
176	0.272	0.570	5.144	19.47	56.300	0.120	1.803	6.544	0.056	0	0	0	90.279	PC11-4_62-64-5-3	Euhedral spinel
177	0.465	1.089	5.446	0.845	79.224	0.169	0.999	1.693	0.013	0.042	0.112	0	90.097	PC11-4_62-64-5-4	Euhedral spinel
178	0.702	0.761	3.592	0.279	81.011	0.112	0.550	1.152	0.043	0	0.052	0	88.254	PC11-4_62-64-5-5	Euhedral spinel
179	0.504	0.973	4.328	1.482	77.810	0.192	0.889	2.072	0	0.012	0	0.017	88.279	PC11-4_62-64-5-6	Euhedral spinel
180	0.404	0.728	4.800	11.683	65.994	0.192	0.676	3.494	0	0.022	0.053	0	88.046	PC11-4_62-64-5-7	Euhedral spinel
181	0.435	0.813	5.616	4.436	74.102	0.185	0.868	2.710	0	0	0.057	0	89.222	PC11-4_62-64-5-8	Euhedral spinel
182	0.415	1.028	5.454	4.555	73.882	0.182	0.841	2.498	0	0	0.016	0.008	88.879	PC11-4_62-64-5-9	Euhedral spinel
183	0.605	0.909	3.681	0.321	79.240	0.102	0.353	0.632	0	0	0.016	0.068	85.927	PC11-4_62-64-5-10	Euhedral spinel
184	0.551	0.881	5.506	0.601	73.774	0.251	0.553	1.283	0	0.012	0	0.045	83.457	PC11-4_62-64-5-11	Euhedral spinel
185	0.521	0.903	4.819	0.250	78.339	0.168	0.456	1.230	0	0	0	0	86.686	PC11-4_62-64-5-12	Euhedral spinel
186	0.390	0.743	5.856	11.499	66.179	0.159	0.805	2.468	0	0.027	0	0.025	88.151	PC11-4_62-64-5-13	Euhedral spinel
187	3.698	1.952	2.048	0.200	52.952	0.210	0.176	0.914	0.315	0	0.028	0.595	63.088	PC11-4_62-64-5-14	Euhedral spinel
188	3.432	1.835	1.015	0.106	25.147	0.215	0.085	0.220	0.241	0.042	0	0.442	32.780	PC11-4_62-64-5-15	Euhedral spinel
189	4.967	1.700	2.033	0.123	45.147	0.114	0	1.044	0.284	0.094	0.118	0.713	56.337	PC11-4_62-64-5-16	Matrix
190	2.158	1.208	4.300	2.222	39.259	0.094	0.575	1.510	0.110	0	0.031	0.607	52.074	PC11-4_62-64-5-17	Matrix
191	4.391	1.707	1.959	0.534	27.920	0.213	0.080	1.152	0.294	0.085	0.105	0.815	39.255	PC11-4_62-64-5-18	Matrix
192	3.277	1.035	1.096	0.108	26.942	0.127	0.009	0.627	0.246	0.015	0.071	0.513	34.066	PC11-4_62-64-5-19	Matrix
193	0.478	0.830	4.165	2.219	77.167	0.260	1.007	1.841	0	0.015	0.064	0	88.046	PC11-4_62-64-6-1	Euhedral spinel
194	0.311	0.617	5.359	11.817	64.697	0.235	1.730	3.819	0	0.044	0	0	88.629	PC11-4_62-64-6-2	Euhedral spinel
195	0.302	0.472	5.673	33.426	41.984	0.184	1.675	8.141	0	0	0.044	0	91.901	PC11-4_62-64-6-3	Euhedral spinel
196	0.260	0.478	4.713	25.023	50.695	0.273	1.639	7.593	0	0	0	0	90.674	PC11-4_62-64-6-4	Euhedral spinel
197	0.384	1.067	3.144	0.300	79.558	0.171	0.952	1.766	0	0	0	0.045	87.387	PC11-4_62-64-6-5	Dendritic spinel
198	0.495	1.007	4.223	3.064	73.885	0.132	1.019	1.074	0.005	0	0	0	84.904	PC11-4_62-64-6-6	Euhedral spinel
199	0.387	0.718	4.812	3.321	75.662	0.089	1.231	2.645	0	0.010	0	0	88.875	PC11-4_62-64-6-7	Euhedral spinel
200	0.430	0.819	3.626	4.025	75.399	0.188	1.046	2.144	0	0	0.08	0.042	87.799	PC11-4_62-64-6-8	Euhedral spinel
201	0.430	0.848	4.241	0.722	78.068	0.198	1.045	1.589	0	0	0	0	87.141	PC11-4_62-64-6-9	Dendritic spinel
202	0.376	0.471	5.669	23.615	53.338	0.272	1.667	4.971	0	0	0	0	90.379	PC11-4_62-64-6-10	Euhedral spinel
203	3.036	0.981	3.607	1.092	69.404	0.156	0.859	1.749	0.038	0.115	0.048	0.158	81.243	PC11-4_62-64-6-11	Matrix
204	4.227	2.449	1.026	0.088	27.418	0.222	0.073	0.846	0.341	0.007	0.052	0.795	37.544	PC11-4_62-64-6-12	Matrix
205	4.668	2.327	1.452	0.168	34.430	0.124	0.067	1.047	0.416	0.025	0.119	0.854	45.697	PC11-4_62-64-6-13	Matrix
206	5.501	1.935	2.344	0.168	37.552	0.165	0.174	1.223	0.268	0.129	0.115	0.793	50.367	PC11-4_62-64-6-14	Matrix
207	0.751	0.651	8.783	35.007	41.740	0.254	1.495	6.128	0.074	0.025	0	0.052	94.960	PC11-4_62-64-8-1	Euhedral spinel
208	0.462	1.786	10.067	0.262	76.316	0.129	0.629	0.893	0	0	0	0	90.544	PC11-4_62-64-8-2	Euhedral spinel
209	0.457	1.708	11.652	4.944	70.626	0.130	0.569	1.601	0	0	0.04	0	91.727	PC11-4_62-64-8-3	Euhedral spinel
210	0.460	1.854	9.084	0.150	75.641	0.185	0.492	0.694	0.030	0	0	0	88.590	PC11-4_62-64-8-4	Euhedral spinel
211	0.610	1.926	9.926	0.240	76.155	0.136	0.668	0.780	0	0.017	0	0.037	90.495	PC11-4_62-64-8-5	Euhedral spinel
212	0.306	0.826	10.671	24.594	51.686	0.240	0.900	3.029	0.015	0	0.018	0.035	92.320	PC11-4_62-64-8-6	Euhedral spinel
213	0.436	1.537	12.206	4.684	70.964	0.163	0.727	1.185	0.005	0.034	0	0.056	91.997	PC11-4_62-64-8-7	Euhedral spinel
214	0.539	1.869	9.935	0.815	75.392	0.179	0.538	0.847	0	0	0.019	0.006	90.139	PC11-4_62-64-8-8	Euhedral spinel
215	0.131	0.807	9.741	24.607	52.015	0.183	1.166	3.410	0.025	0	0.012	0.019	92.116	PC11-4_62-64-8-9	Euhedral spinel
216	0.458	1.754	10.245	0.975	75.448	0.162	1.274	0.897	0.038	0	0.013	0	91.264	PC11-4_62-64-8-10	Euhedral spinel
217	0.525	1.841	9.200	0.132	77.459	0.208	0.426	0.808	0	0.002	0	0	90.601	PC11-4_62-64-8-11	Euhedral spinel
218	0.647	1.941	10.503	0.123	76.474	0.119	0.357	0.658	0	0.012	0	0	90.834	PC11-4_62-64-8-12	Euhedral spinel
219	12.849	3.475	0.935	0.043	48.208	0.476	0.067	2.101	0.761	0.062	0.116	0.914	70.007	PC11-4_62-64-8-13	Matrix
220	9.219	3.143	0.959	0.070	36.858	0.274	0.125	1.468	0.579	0	0.139	0.903	53.737	PC11-4_62-64-8-14	Matrix
221	12.217	3.546	0.994	0.040	43.845	0.403	0.129	1.767	0.720	0.032	0.109	1.018	64.820	PC11-4_62-64-8-15	Matrix
222	11.185	3.148	1.005	0.043	42.031	0.551	0	1.720	0.745	0.042	0.08	0.985	61.535	PC11-4_62-64-8-16	Matrix
223	11.783	3.334	1.026	0.086	44.238	0.372	0.034	1.624	0.670	0.042	0.133	0.958	64.300	PC11-4_62-64-8-17	Matrix
224	93.536	0	0.109	0	0.412	0	0	0.009	0	0.017	0	0	94.083	PC11-4_62-64-8-18	Quartz
225	13.024	3.522	1.043	0	46.248	0.440	0.107	1.619	0.797	0.007	0.118	1.036	67.961	PC11-4_62-64-8-19	Matrix
226	6.578	1.917	2.156	0.039	35.720	0.326	0.009	0.957	0.340	0.050	0.132	0.672	48.896	PC11-4_62-64-8-20	Matrix
227	11.858	3.408	0.871	0.033	43.192	0.450	0.159	2.142	0.567	0.062	0.174	0.895	63.811	PC11-4_62-64-8-21	Matrix
228	14.908	2.940	2.254	0.064	44.488	0.479	0.068	2.021	0.754	0.141	0.125	0.853	69.095	PC11-4_62-64-8-22	Matrix
229	0.301	0.552	8.513	36.079	39.953	0.240	1.502	6.567	0.025	0.024	0	0.012	93.768	PC11-4_62-64-10-1	Euhedral spinel
230	0.388	0.752	9.496	34.728	38.994	0.286	1.396	5.213	0	0.005	0.078	0	91.336	PC11-4_62-64-10-2	Euhedral spinel
231	0.407	1.890	13.506	1.850	70.471	0.183	0.995	1.895	0	0.007	0.03	0.012	91.246	PC11-4_62-64-10-3	Euhedral spinel
232	0.417	2.146	14.325	0.686	70.503	0.170	0.917	2.003	0	0	0.033	0.014	91.214	PC11-4_62-64-10-4	Euhedral spinel
233	0.336	0.757	9.687	33.927	40.521	0.357	1.463	6.314	0.071	0	0	0.006	93.439	PC11-4_62-64-10-5	Euhedral spinel
234	1.073	1.680	16.523	2.913	65.055	0.307	1.235	2.988	0	0.089	0	0.057	91.920	PC11-4_62-64-10-6	Euhedral spinel
235	0.376	0.659	8.852	34.282	38.956	0.204	1.634	7.320	0.051	0	0.046	0	92.380	PC11-4_62-64-10-7	Euhedral spinel
236	0.325	0.931	8.844	32.040	42.876	0.157	1.639	7.176	0	0.012	0	0	94.000	PC11-4_62-64-10-8	Euhedral spinel
237	8.726	2.777	2.427	0.184	40.621	0.212	0.015	1.521	0.480	0.294	0.124	1.138	58.519	PC11-4_62-64-10-9	Matrix
238	8.769	2.885	2.885	0.173	45.738	0.222	0.043	1.519	0.546	0.149	0.113	1.324	64.366	PC11-4_62-64-10-10	Matrix

FeO*, total iron as FeO. Grey shaded columns are not extraterrestrial materials.

Table S4 (continued)

Analytical No.	SiO_2 (wt%)	TiO_2 (wt%)	Al_2O_3 (wt%)	Cr_2O_3 (wt%)	FeO* (wt%)	MnO (wt%)	NiO (wt%)	MgO (wt%)	CaO (wt%)	K_2O (wt%)	Na_2O (wt%)	P_2O_5 (wt%)	Total (wt%)	Comment and sample no.	Target mineral
239	12.916	2.367	2.880	0.108	34.477	0.269	0.031	1.699	0.453	0.738	0.173	0.756	56.867	PC11-4_62-64-10-11	Matrix
240	0.189	0.606	7.278	38.185	39.448	0.140	1.667	7.855	0	0.005	0	0	95.373	PC11-4_64-66-1-1	Euhedral spinel
241	0.240	0.611	7.727	33.861	42.678	0.156	1.744	7.137	0.005	0.010	0.023	0.011	94.203	PC11-4_64-66-1-2	Euhedral spinel
242	0.527	0.054	0.141	0.119	87.526	0.202	0.146	0.521	0	0.024	0.075	0	89.335	PC11-4_64-66-1-3	Dendritic spinel
243	0.434	1.439	10.746	4.810	70.956	0.211	1.226	2.325	0	0	0.018	0	92.165	PC11-4_64-66-1-4	Euhedral spinel
244	0.478	0.693	0.581	0.088	84.671	0.147	0.144	0.232	0	0	0.016	0.005	87.055	PC11-4_64-66-1-5	Dendritic spinel
245	0.234	0.687	9.711	26.159	50.202	0.343	1.473	4.332	0	0.029	0	0.002	93.172	PC11-4_64-66-1-6	Euhedral spinel
246	18.69	2.591	0.977	0.063	55.478	0.278	0.172	2.271	0.841	0.008	0.208	1.049	82.626	PC11-4_64-66-1-7	Matrix
247	16.886	1.689	1.566	0.056	51.260	0.105	0.098	1.979	0.712	0.234	0.231	0.908	75.724	PC11-4_64-66-1-8	Matrix
248	14.012	2.362	0.904	0.118	49.647	0.162	0.306	2.623	0.545	0.040	0.151	0.855	71.725	PC11-4_64-66-1-9	Matrix
249	18.877	1.439	1.016	0.141	54.988	0.186	0.123	2.268	0.704	0.055	0.155	1.011	80.963	PC11-4_64-66-1-10	Matrix
250	0.228	0.634	8.064	32.283	44.105	0.212	1.737	6.228	0	0.022	0.035	0	93.548	PC11-4_64-66-4-1	Euhedral spinel
251	0.321	0.652	7.469	35.982	41.852	0.232	1.672	7.570	0	0.022	0	0	95.772	PC11-4_64-66-4-2	Euhedral spinel
252	0.295	0.720	9.565	27.436	48.321	0.179	1.670	5.057	0.005	0	0.015	0.006	93.269	PC11-4_64-66-4-3	Euhedral spinel
253	0.447	1.895	8.202	1.342	75.586	0.105	1.201	1.773	0	0.046	0	0.024	90.621	PC11-4_64-66-4-4	Euhedral spinel
254	0.231	0.883	8.860	21.969	54.634	0.175	1.042	3.864	0	0	0	0.037	91.695	PC11-4_64-66-4-5	Euhedral spinel
255	0.548	1.676	7.142	0.556	78.385	0.144	0.873	1.049	0	0	0	0.031	90.404	PC11-4_64-66-4-6	Euhedral spinel
256	0.328	0.929	8.671	16.334	59.917	0.178	1.721	3.338	0	0	0	0	91.416	PC11-4_64-66-4-7	Euhedral spinel
257	0.977	0.609	0.489	0.149	82.393	0.094	0.110	0.344	0	0.019	0.075	0.041	85.300	PC11-4_64-66-4-8	Dendritic spinel
258	0.328	0.618	7.499	39.399	38.827	0.259	0.911	7.361	0	0.039	0	0	95.241	PC11-4_64-66-5-1	Euhedral spinel
259	0.341	0.670	7.765	35.234	41.428	0.246	0.984	7.035	0	0.056	0.003	0.019	93.781	PC11-4_64-66-5-2	Euhedral spinel
260	0.257	1.558	11.434	15.483	59.507	0.208	0.741	2.690	0	0.010	0	0.003	91.891	PC11-4_64-66-5-3	Euhedral spinel
261	0.303	0.954	9.597	30.183	46.011	0.229	0.835	5.198	0.008	0	0	0.009	93.327	PC11-4_64-66-5-4	Euhedral spinel
262	0.454	2.223	12.071	1.391	73.663	0.132	0.460	1.169	0	0	0	0.001	91.564	PC11-4_64-66-5-5	Euhedral spinel
263	0.532	2.321	11.297	0.515	75.560	0.234	0.319	0.917	0	0.024	0.009	0	91.728	PC11-4_64-66-5-6	Euhedral spinel
264	0.766	0.705	9.290	34.797	41.072	0.216	1.283	6.386	0.061	0.032	0	0.008	94.616	PC11-4_64-66-5-7	Euhedral spinel
265	0.510	2.292	10.775	0.545	76.109	0.148	0.556	1.043	0	0.024	0.108	0.016	92.126	PC11-4_64-66-5-8	Euhedral spinel
266	0.278	0.650	9.423	37.546	40.941	0.262	1.302	5.983	0	0.010	0	0	96.395	PC11-4_64-66-5-9	Euhedral spinel
267	0.858	2.693	9.760	0.206	78.068	0.217	0.219	0.461	0	0	0.044	0	92.526	PC11-4_64-66-5-10	Euhedral spinel
268	0.233	0.635	9.198	38.401	39.345	0.259	1.397	6.531	0	0.046	0	0.043	96.088	PC11-4_64-66-5-11	Euhedral spinel
269	0.777	2.438	15.779	0.676	69.453	0.182	0.437	1.027	0	0.027	0.042	0.009	90.847	PC11-4_64-66-5-12	Euhedral spinel
270	1.202	2.633	12.893	0.395	74.284	0.218	0.313	0.698	0	0	0	0	92.636	PC11-4_64-66-5-13	Euhedral spinel
271	0.658	2.552	12.775	0.555	75.338	0.092	0.427	0.628	0	0	0	0	93.025	PC11-4_64-66-5-14	Euhedral spinel
272	0.229	0.892	9.969	31.772	45.782	0.169	0.989	5.240	0	0.068	0.099	0.005	95.214	PC11-4_64-66-5-15	Euhedral spinel
273	0.408	0.869	10.945	29.526	46.602	0.225	0.763	4.531	0	0	0.038	0	93.907	PC11-4_64-66-5-16	Euhedral spinel
274	0.389	2.544	10.79	1.607	74.150	0.082	0.457	1.085	0	0	0.006	0.036	91.146	PC11-4_64-66-5-17	Euhedral spinel
275	0.217	0.788	10.539	33.007	44.322	0.352	1.066	5.038	0.023	0	0.035	0	95.387	PC11-4_64-66-5-18	Euhedral spinel
276	0.477	2.052	12.393	4.654	69.473	0.265	0.334	1.408	0	0	0	0.005	91.061	PC11-4_64-66-5-19	Euhedral spinel
277	0.652	1.914	8.064	0.197	76.703	0.204	0.327	0.533	0	0	0.016	0	88.610	PC11-4_64-66-5-20	Euhedral spinel
278	14.839	2.839	0.961	0.169	46.371	0.520	0.049	2.895	0.735	0.027	0.223	0.934	70.562	PC11-4_64-66-5-21	Matrix
279	13.202	1.728	2.148	0.117	48.396	0.319	0.097	1.581	0.565	0.070	0.203	0.611	69.037	PC11-4_64-66-5-22	Matrix
280	18.171	2.365	0.873	0.138	54.265	0.546	0.073	2.467	0.870	0.055	0.265	0.945	81.033	PC11-4_64-66-5-23	Matrix
281	9.110	2.651	2.991	0.511	58.463	0.317	0.167	3.666	0.566	0.005	0.158	1.606	80.211	PC11-4_64-66-5-24	Matrix
282	21.156	1.978	0.838	0.112	55.265	0.499	0.177	2.313	0.894	0.030	0.205	0.846	84.313	PC11-4_64-66-5-25	Matrix
283	19.563	2.186	0.811	0.138	55.237	0.523	0.088	2.717	0.866	0.065	0.131	0.900	83.225	PC11-4_64-66-5-26	Matrix
284	18.182	2.216	0.961	0.141	54.887	0.556	0.265	2.594	0.877	0.035	0.201	0.842	81.757	PC11-4_64-66-5-27	Matrix
285	18.499	2.253	0.867	0.220	54.343	0.691	0.107	2.801	0.899	0.037	0.212	0.918	81.847	PC11-4_64-66-5-28	Matrix
286	6.041	2.300	2.685	0.201	46.897	0.143	0	2.191	0.416	0.047	0.168	1.316	62.405	PC11-4_64-66-5-29	Matrix
287	8.012	2.900	4.149	0.201	58.290	0.123	0.103	1.256	0.502	0.054	0.211	1.249	77.050	PC11-4_64-66-5-30	Matrix
288	17.695	1.758	4.026	0.162	50.051	0.132	0.012	1.603	0.438	1.076	0.283	0.947	78.183	PC11-4_64-66-5-31	Matrix
289	11.755	2.184	2.706	0.168	53.034	0.187	0.049	1.694	0.663	0.258	0.382	1.129	74.209	PC11-4_64-66-5-32	Matrix
290	16.990	1.685	3.530	0.119	49.560	0.192	0.055	1.908	0.534	0.907	0.306	0.944	76.730	PC11-4_64-66-5-33	Matrix
291	17.564	1.948	3.283	0.067	45.941	0.205	0.080	2.273	0.529	0.820	0.331	0.972	74.013	PC11-4_64-66-5-34	Matrix
292	10.517	1.742	3.067	0.175	57.132	0.147	6.896	1.431	0.629	0.125	0.152	1.461	83.474	PC11-4_64-66-5-35	Spherical spinel
293	10.307	2.107	3.227	0.116	57.345	0.134	3.296	1.306	0.587	0.102	0.312	1.314	80.153	PC11-4_64-66-5-36	Spherical spinel
294	9.863	1.035	1.283	0.152	49.495	0.272	23.288	0.881	0.524	0.083	0.178	0.808	87.862	PC11-4_64-66-5-37	Spherical spinel
Min. (*n* = 284)	0.080	0	0.109	0	0	0	0	0	0	0	0	0	32.780		
Max. (*n* = 284)	98.455	51.114	40.967	41.800	92.412	0.922	23.288	34.355	20.946	5.111	11.747	1.778	103.003		
Avg. (*n* = 284)	8.966	1.635	6.558	6.108	54.600	0.219	0.656	2.416	0.506	0.203	0.220	0.310	82.397		
1SD (*n* = 284)	17.611	3.091	6.628	11.385	22.133	0.165	1.523	2.983	2.116	0.748	0.916	0.472	14.252		

FeO*, total iron as FeO.

Table S5: Ferric- and ferrous-iron components with endmember compositions when assuming spinel stoichiometry (AB_2O_4) for seven constituent elements (Mg, Al, Fe, Cr, Ni, Mn and Ti) using EPMA data of each spinel grain.

Analytical No.	Fe^{2+}	Fe^{3+}	Fe^{3+}/Fe_{tot}	Spinel (%)	Hercynite (%)	Picrochromite (%)	Chromite (%)	Magnesioferrite (%)	Magnetite (%)	Trevorite (%)	Jacobsite (%)	Ulvöspinel (%)	Comment and sample no.	Target mineral
4	0.832	1.217	0.594	3.4	18.9	2.0	11.2	9.7	45.7	4.8	0.6	3.7	PC11-4_56-58-1-1	Euhedral spinel
5	0.858	1.305	0.603	3.1	20.0	1.0	6.7	9.3	51.3	4.4	0.3	3.9	PC11-4_56-58-1-2	Euhedral spinel
6	0.585	0.511	0.466	6.9	12.1	19.6	34.4	9.8	9.2	5.8	0.8	1.4	PC11-4_56-58-1-3	Euhedral spinel
7	0.858	1.271	0.597	3.0	19.0	1.4	8.9	9.1	49.5	4.3	0.6	4.2	PC11-4_56-58-1-4	Euhedral spinel
8	0.853	1.234	0.591	3.2	20.3	1.5	9.7	8.8	48.2	4.2	0.5	3.6	PC11-4_56-58-1-5	Euhedral spinel
9	0.950	1.894	0.666	0.1	2.1	0.0	0.3	4.6	87.1	2.2	0.8	2.7	PC11-4_56-58-1-6	Dendritic spinel
10	0.923	1.890	0.672	0.2	2.3	0.0	0.5	6.0	84.4	3.1	0.9	2.5	PC11-4_56-58-1-7	Dendritic spinel
11	0.957	1.931	0.669	0.0	1.3	0.0	0.5	3.2	90.8	2.0	0.6	1.6	PC11-4_56-58-1-8	Dendritic spinel
12	0.985	1.922	0.661	0.0	1.1	0.0	0.4	1.5	92.2	1.9	0.6	2.4	PC11-4_56-58-1-9	Dendritic spinel
13	0.963	1.894	0.663	0.1	1.9	0.0	0.5	3.4	88.3	2.3	0.7	2.8	PC11-4_56-58-1-10	Dendritic spinel
14	0.973	1.898	0.661	0.1	1.6	0.0	0.4	3.0	89.2	2.0	0.7	3.0	PC11-4_56-58-1-11	Dendritic spinel
20	0.815	1.278	0.611	3.4	15.4	2.4	10.7	12.4	47.1	3.7	0.7	4.2	PC11-4_56-58-2-2	Euhedral spinel
21	0.670	0.594	0.470	5.9	13.6	14.8	34.0	9.6	15.5	3.8	0.8	2.0	PC11-4_56-58-2-3	Euhedral spinel
22	0.841	1.213	0.591	3.3	18.0	2.2	12.3	9.9	46.7	3.4	0.6	3.6	PC11-4_56-58-2-4	Euhedral spinel
23	0.928	1.516	0.620	1.8	15.4	0.2	1.5	8.4	65.2	2.0	0.3	5.3	PC11-4_56-58-2-5	Euhedral spinel
24	0.861	0.954	0.526	2.8	17.8	3.8	24.5	6.9	37.0	2.9	1.0	3.4	PC11-4_56-58-2-6	Euhedral spinel
25	0.851	1.470	0.633	2.3	13.5	1.1	6.4	11.2	58.8	2.7	0.8	3.3	PC11-4_56-58-2-7	Euhedral spinel
26	0.670	0.805	0.546	5.3	11.9	12.4	28.0	13.0	22.9	3.3	1.0	2.1	PC11-4_56-58-2-8	Euhedral spinel
27	0.626	0.658	0.513	5.9	11.3	16.5	31.7	11.9	16.1	4.1	0.8	1.8	PC11-4_56-58-2-9	Euhedral spinel
28	0.908	1.501	0.623	2.0	16.3	0.2	1.4	8.7	63.0	2.6	0.6	5.0	PC11-4_56-58-2-10	Euhedral spinel
35	0.921	1.502	0.620	1.9	16.5	0.1	0.9	8.3	63.7	2.6	0.4	5.5	PC11-4_56-58-4-1	Euhedral spinel
36	0.860	1.427	0.624	2.6	17.2	0.6	4.2	9.8	56.6	4.3	0.6	4.0	PC11-4_56-58-4-2	Euhedral spinel
37	0.785	0.653	0.454	3.8	16.4	8.4	36.6	6.5	21.1	4.5	0.6	2.2	PC11-4_56-58-4-3	Euhedral spinel
38	0.848	1.449	0.631	3.1	16.8	0.5	2.9	11.9	56.5	3.7	0.4	4.3	PC11-4_56-58-4-4	Euhedral spinel
39	0.656	0.766	0.539	6.2	14.1	12.1	27.3	12.3	20.2	5.2	0.5	2.0	PC11-4_56-58-4-5	Euhedral spinel
40	0.936	1.472	0.611	1.6	19.1	0.0	0.6	6.0	63.8	2.7	1.0	5.0	PC11-4_56-58-4-6	Euhedral spinel
47	0.494	0.407	0.452	7.8	8.7	29.4	32.6	10.2	5.8	3.6	0.8	1.2	PC11-4_56-58-8-1	Euhedral spinel
48	0.534	0.498	0.482	7.1	9.5	24.4	32.3	11.5	8.1	4.5	0.9	1.8	PC11-4_56-58-8-2	Euhedral spinel
49	0.845	1.084	0.562	4.1	20.0	2.9	14.4	9.9	41.4	2.2	0.7	4.3	PC11-4_56-58-8-3	Euhedral spinel
50	0.815	0.995	0.550	4.4	19.2	4.3	18.6	10.0	36.3	2.6	0.9	3.6	PC11-4_56-58-8-4	Euhedral spinel
51	0.886	1.289	0.593	3.2	20.0	1.0	6.4	9.5	52.3	1.6	1.0	4.9	PC11-4_56-58-8-5	Euhedral spinel
52	0.975	1.135	0.538	1.7	22.2	0.9	12.3	4.4	50.7	1.2	0.5	6.2	PC11-4_56-58-8-6	Euhedral spinel
59	0.628	0.621	0.497	6.1	11.6	17.1	32.2	11.4	15.2	3.4	1.0	1.9	PC11-4_58-60-4-1	Euhedral spinel
60	0.897	1.499	0.626	2.2	15.6	0.2	1.1	10.1	61.1	3.2	0.6	6.0	PC11-4_58-60-4-2	Euhedral spinel
61	0.868	1.165	0.573	3.1	19.2	2.1	12.7	8.8	45.5	3.1	0.8	4.7	PC11-4_58-60-4-3	Dendritic spinel
62	0.886	1.416	0.615	2.3	15.6	0.8	5.3	9.9	57.4	3.1	0.4	5.2	PC11-4_58-60-4-4	Euhedral spinel
63	0.942	1.892	0.668	0.1	1.8	0.0	0.4	6.1	86.0	1.4	1.2	3.0	PC11-4_58-60-4-5	Dendritic spinel
64	0.968	1.912	0.664	0.1	1.7	0.0	0.4	3.0	90.2	1.5	0.8	2.3	PC11-4_58-60-4-6	Dendritic spinel
65	0.537	0.522	0.492	8.4	11.0	22.8	30.1	11.9	9.4	3.9	0.8	1.6	PC11-4_58-60-4-7	Euhedral spinel
66	0.899	1.217	0.575	2.7	20.7	1.2	9.5	7.6	49.7	3.1	0.5	5.0	PC11-4_58-60-4-8	Euhedral spinel
67	0.699	0.883	0.558	5.5	13.7	9.7	24.0	13.5	26.5	3.2	0.9	2.8	PC11-4_58-60-4-9	Dendritic spinel
68	0.615	0.604	0.496	6.9	12.1	17.7	31.2	11.6	14.3	3.6	0.6	2.0	PC11-4_58-60-4-10	Euhedral spinel
110	0.975	1.136	0.538	1.8	33.8	0.1	1.3	3.2	49.9	2.9	0.8	6.3	PC11-4_60-62-7-1	Euhedral spinel
111	1.032	1.280	0.554	0.8	27.2	0.0	0.4	2.0	60.4	1.2	0.4	7.6	PC11-4_60-62-7-2	Euhedral spinel
112	0.919	1.239	0.574	2.8	26.2	0.3	2.6	6.6	50.9	3.6	1.0	6.1	PC11-4_60-62-7-3	Euhedral spinel
113	0.990	1.265	0.561	1.1	29.0	0.0	1.0	2.5	57.8	2.0	0.9	5.6	PC11-4_60-62-7-4	Euhedral spinel
114	0.926	1.243	0.573	2.5	28.3	0.1	1.5	5.6	52.0	3.8	0.8	5.4	PC11-4_60-62-7-5	Euhedral spinel
115	1.003	1.354	0.575	0.8	25.9	0.0	0.3	2.1	63.4	1.2	1.0	5.3	PC11-4_60-62-7-6	Euhedral spinel
116	1.006	1.097	0.522	0.6	37.8	0.0	0.8	1.0	50.3	2.5	1.1	5.8	PC11-4_60-62-7-7	Euhedral spinel
117	1.103	1.637	0.598	0.1	4.8	0.0	0.0	1.8	79.0	0.7	0.3	13.2	PC11-4_60-62-7-8	Dendritic spinel
118	0.995	1.854	0.651	0.0	4.7	0.0	0.3	1.0	90.1	1.1	0.5	2.2	PC11-4_60-62-7-9	Dendritic spinel
119	0.987	1.163	0.541	2.0	32.4	0.0	0.8	3.8	52.2	1.7	0.5	6.6	PC11-4_60-62-7-10	Dendritic spinel
120	1.021	1.331	0.566	0.2	26.9	0.0	0.6	0.6	63.2	0.1	2.7	5.7	PC11-4_60-62-7-11	Euhedral spinel
124	0.757	1.460	0.659	3.5	13.1	1.4	5.2	16.2	49.8	6.3	0.7	3.8	PC11-4_60-62-10-1	Euhedral spinel
125	0.712	1.450	0.671	3.9	11.2	2.3	6.6	19.7	46.5	5.5	0.8	3.4	PC11-4_60-62-10-2	Euhedral spinel
126	0.916	1.585	0.634	1.3	14.6	0.1	0.9	6.8	68.2	3.5	0.8	4.0	PC11-4_60-62-10-3	Euhedral spinel
127	0.769	1.227	0.615	3.1	12.7	4.0	16.4	12.6	43.1	4.6	1.1	2.4	PC11-4_60-62-10-4	Euhedral spinel
128	0.929	1.583	0.630	1.1	14.0	0.2	2.0	6.1	69.8	2.6	0.7	3.5	PC11-4_60-62-10-5	Euhedral spinel
129	0.666	0.993	0.599	4.1	9.8	10.2	24.3	15.2	28.5	5.1	0.8	2.0	PC11-4_60-62-10-6	Euhedral spinel
130	0.986	1.822	0.649	0.2	3.2	0.0	0.2	5.7	84.6	0.2	0.7	5.3	PC11-4_60-62-10-7	Dendritic spinel
131	0.633	0.937	0.597	5.0	10.0	12.0	23.8	16.5	25.0	4.2	1.1	2.3	PC11-4_60-62-10-8	Euhedral/dendritic spinel
132	0.826	0.993	0.546	3.0	16.8	4.2	23.7	7.8	36.8	4.2	0.8	2.7	PC11-4_60-62-10-9	Euhedral/dendritic spinel
133	0.850	1.424	0.626	2.4	14.1	1.3	7.5	10.9	56.4	3.2	0.7	3.5	PC11-4_60-62-10-10	Euhedral spinel
138	0.478	0.410	0.461	8.0	8.4	30.1	31.9	10.5	5.3	4.1	0.6	1.1	PC11-4_60-62-10-15	Euhedral spinel
139	0.494	0.410	0.454	7.3	8.3	29.3	33.4	10.1	5.3	4.1	1.0	1.2	PC11-4_60-62-10-16	Euhedral spinel
140	0.842	1.059	0.557	3.6	18.5	3.4	17.6	9.2	40.1	2.9	0.8	4.0	PC11-4_60-62-10-17	Euhedral spinel
141	0.788	0.932	0.542	5.1	21.7	4.4	18.6	9.6	31.4	4.6	1.0	3.5	PC11-4_60-62-10-18	Euhedral spinel
142	0.939	1.462	0.609	1.7	15.5	0.3	2.6	7.8	62.0	2.7	0.6	6.9	PC11-4_60-62-10-19	Euhedral spinel
143	0.868	1.301	0.600	2.8	18.1	1.3	8.0	9.5	51.2	3.6	0.8	4.8	PC11-4_60-62-10-20	Euhedral spinel

Table S5 (continued)

Analytical No.	Fe^{2+}	Fe^{3+}	Fe^{3+}/Fe_{tot}	Spinel (%)	Hercynite (%)	Picrochromite (%)	Chromite (%)	Magnesioferrite (%)	Magnetite (%)	Trevorite (%)	Jacobsite (%)	Ulvöspinel (%)	Comment and sample no.	Target mineral
144	1.025	1.586	0.608	0.5	11.5	0.0	0.6	3.6	74.2	0.8	0.7	8.1	PC11-4_60-62-10-21	Euhedral spinel
145	1.022	1.438	0.585	0.7	19.0	0.0	0.4	3.0	66.9	1.6	0.5	8.0	PC11-4_60-62-10-22	Euhedral spinel
146	0.923	1.271	0.579	2.1	20.3	0.8	7.7	6.5	53.3	3.3	0.4	5.5	PC11-4_60-62-10-23	Euhedral spinel
147	0.965	1.941	0.668	0.0	1.4	0.0	0.4	3.0	92.5	0.7	0.9	1.1	PC11-4_60-62-10-24	Dendritic spinel
148	0.983	1.904	0.660	0.1	1.5	0.0	0.4	3.4	90.7	0.2	0.9	2.8	PC11-4_60-62-10-25	Dendritic spinel
149	0.964	1.843	0.657	0.2	4.9	0.0	0.6	3.6	86.6	1.4	0.5	2.2	PC11-4_60-62-10-26	Euhedral spinel
150	0.992	1.730	0.636	0.2	8.3	0.0	0.8	2.5	81.8	1.2	1.1	4.2	PC11-4_60-62-10-27	Dendritic spinel
151	0.949	1.835	0.659	0.3	5.1	0.0	0.4	5.3	84.6	1.1	0.8	2.4	PC11-4_60-62-10-28	Dendritic spinel
152	1.003	1.350	0.574	1.2	23.5	0.0	0.3	3.6	61.6	1.4	0.8	7.4	PC11-4_60-62-10-29	Euhedral spinel
153	0.968	1.402	0.591	1.4	20.8	0.0	0.7	4.8	61.4	2.9	1.0	6.9	PC11-4_60-62-10-30	Euhedral spinel
154	0.767	1.930	0.716	0.1	2.0	0.0	0.3	3.4	72.0	20.1	0.9	1.2	PC11-4_60-62-10-31	Euhedral/dendritic spinel
155	0.992	1.606	0.618	0.6	14.4	0.0	0.2	3.4	75.6	0.5	0.8	4.5	PC11-4_60-62-10-32	Euhedral/dendritic spinel
156	1.007	1.386	0.579	0.9	23.4	0.0	0.3	2.8	64.8	0.8	0.9	6.1	PC11-4_60-62-10-33	Euhedral/dendritic spinel
157	0.966	1.809	0.652	0.2	6.0	0.0	0.3	3.4	84.4	0.5	2.2	2.9	PC11-4_60-62-10-34	Euhedral/dendritic spinel
158	1.024	1.384	0.575	0.7	23.2	0.0	0.3	2.2	65.6	0.9	0.4	6.6	PC11-4_60-62-10-35	Euhedral spinel
159	0.971	1.913	0.663	0.1	2.1	0.0	0.5	3.0	91.2	0.6	0.8	1.7	PC11-4_60-62-10-36	Dendritic spinel
160	0.791	0.818	0.509	6.0	23.7	5.1	20.4	9.0	27.2	3.9	0.7	3.9	PC11-4_60-62-10-37	Euhedral spinel
161	0.985	1.355	0.579	1.6	23.0	0.0	0.6	4.8	60.9	1.4	0.7	7.0	PC11-4_60-62-10-38	Euhedral spinel
162	0.982	1.300	0.570	1.5	26.0	0.1	1.1	3.8	58.3	1.8	1.0	6.4	PC11-4_60-62-10-39	Euhedral spinel
163	0.978	1.686	0.633	0.4	11.3	0.0	0.3	3.3	78.9	1.5	0.7	3.7	PC11-4_60-62-10-40	Euhedral/dendritic spinel
174	0.829	1.607	0.660	2.2	11.2	0.5	2.7	13.5	62.8	3.6	0.4	3.1	PC11-4_62-64-5-1	Euhedral spinel
175	0.919	1.678	0.646	1.1	11.5	0.0	0.5	7.4	73.9	1.5	1.1	3.0	PC11-4_62-64-5-2	Euhedral spinel
176	0.524	1.024	0.661	5.7	7.6	14.4	19.3	22.6	21.8	6.3	0.4	1.9	PC11-4_62-64-5-3	Euhedral spinel
177	0.871	1.574	0.644	1.9	13.7	0.2	1.4	10.2	63.9	3.9	0.7	4.0	PC11-4_62-64-5-4	Euhedral spinel
178	0.914	1.712	0.652	1.0	9.9	0.1	0.5	7.8	75.0	2.3	0.5	2.9	PC11-4_62-64-5-5	Euhedral spinel
179	0.838	1.613	0.658	2.0	10.8	0.5	2.5	13.0	63.2	3.6	0.8	3.7	PC11-4_62-64-5-6	Euhedral spinel
180	0.746	1.243	0.625	3.3	10.1	5.4	16.5	16.0	42.9	2.6	0.8	2.6	PC11-4_62-64-5-7	Euhedral spinel
181	0.794	1.455	0.647	3.1	12.8	1.6	6.8	14.7	54.0	3.4	0.8	2.9	PC11-4_62-64-5-8	Euhedral spinel
182	0.818	1.442	0.638	2.8	12.7	1.6	7.1	13.6	54.5	3.3	0.7	3.7	PC11-4_62-64-5-9	Euhedral spinel
183	0.967	1.686	0.635	0.6	10.9	0.0	0.6	4.4	78.0	1.5	0.5	3.6	PC11-4_62-64-5-10	Euhedral spinel
184	0.900	1.564	0.635	1.7	15.4	0.1	1.1	8.2	66.5	2.3	1.1	3.5	PC11-4_62-64-5-11	Euhedral spinel
185	0.915	1.629	0.640	1.4	13.2	0.0	0.5	8.0	70.9	1.9	0.7	3.5	PC11-4_62-64-5-12	Euhedral spinel
186	0.816	1.194	0.594	2.8	13.4	3.7	17.7	10.8	45.2	3.1	0.6	2.6	PC11-4_62-64-5-13	Euhedral spinel
187	0.984	1.582	0.617	0.9	8.2	0.1	0.5	9.3	67.4	1.1	1.3	11.1	PC11-4_62-64-5-14	Euhedral spinel
188	1.122	1.385	0.552	0.5	8.6	0.0	0.6	4.5	60.9	1.0	2.8	21.0	PC11-4_62-64-5-15	Euhedral spinel
193	0.842	1.602	0.656	1.7	10.6	0.6	3.8	11.5	63.4	4.1	1.1	3.1	PC11-4_62-64-6-1	Euhedral spinel
194	0.682	1.227	0.643	3.9	10.8	5.8	16.0	16.9	37.1	6.5	0.9	2.2	PC11-4_62-64-6-2	Euhedral spinel
195	0.464	0.635	0.578	6.6	6.9	26.1	27.2	16.3	9.4	5.4	0.6	1.4	PC11-4_62-64-6-3	Euhedral spinel
196	0.468	0.891	0.656	5.7	6.1	20.3	21.8	22.2	15.8	5.6	1.0	1.5	PC11-4_62-64-6-4	Euhedral spinel
197	0.859	1.714	0.666	1.3	8.3	0.1	0.5	12.2	68.8	3.9	0.7	4.1	PC11-4_62-64-6-5	Dendritic spinel
198	0.908	1.537	0.629	1.1	11.9	0.5	5.8	6.7	65.3	4.3	0.6	3.9	PC11-4_62-64-6-6	Euhedral spinel
199	0.782	1.542	0.664	2.7	11.2	1.2	5.2	15.4	56.5	4.8	0.4	2.6	PC11-4_62-64-6-7	Euhedral spinel
200	0.820	1.564	0.656	1.7	9.0	1.3	6.7	13.0	60.1	4.2	0.8	3.1	PC11-4_62-64-6-8	Euhedral spinel
201	0.860	1.650	0.657	1.5	11.2	0.2	1.3	10.4	67.0	4.3	0.9	3.3	PC11-4_62-64-6-9	Dendritic spinel
202	0.626	0.871	0.582	4.6	9.8	13.0	27.5	14.5	22.3	5.8	1.0	1.5	PC11-4_62-64-6-10	Euhedral spinel
207	0.611	0.492	0.446	7.1	12.9	18.9	34.6	9.4	9.8	4.6	0.8	1.9	PC11-4_62-64-8-1	Euhedral spinel
208	0.972	1.318	0.576	1.7	25.7	0.0	0.4	4.4	58.6	2.3	0.5	6.2	PC11-4_62-64-8-2	Euhedral spinel
209	0.927	1.112	0.545	3.2	27.0	0.9	7.7	6.4	46.7	2.0	0.5	5.6	PC11-4_62-64-8-3	Euhedral spinel
210	0.991	1.351	0.577	1.3	24.3	0.0	0.3	3.7	61.2	1.9	0.7	6.7	PC11-4_62-64-8-4	Euhedral spinel
211	0.983	1.314	0.572	1.5	25.7	0.0	0.4	3.9	58.8	2.5	0.5	6.7	PC11-4_62-64-8-5	Euhedral spinel
212	0.803	0.641	0.444	4.7	20.9	7.3	32.4	6.4	21.9	3.0	0.8	2.5	PC11-4_62-64-8-6	Euhedral spinel
213	0.942	1.106	0.540	2.4	29.1	0.6	7.5	4.7	47.5	2.6	0.6	5.1	PC11-4_62-64-8-7	Euhedral spinel
214	0.980	1.297	0.570	1.6	25.6	0.1	1.4	4.2	57.9	2.0	0.7	6.5	PC11-4_62-64-8-8	Euhedral spinel
215	0.771	0.678	0.468	4.9	18.7	8.4	31.6	7.6	21.8	3.9	0.6	2.5	PC11-4_62-64-8-9	Euhedral spinel
216	0.946	1.292	0.577	1.7	25.9	0.1	1.7	4.3	55.0	4.7	0.6	6.0	PC11-4_62-64-8-10	Euhedral spinel
217	0.984	1.360	0.580	1.4	23.9	0.0	0.2	4.2	61.4	1.6	0.8	6.5	PC11-4_62-64-8-11	Euhedral spinel
218	1.004	1.292	0.563	1.3	27.2	0.0	0.2	3.2	59.6	1.3	0.5	6.7	PC11-4_62-64-8-12	Euhedral spinel
229	0.582	0.475	0.449	7.4	12.1	20.9	34.3	9.6	8.7	4.7	0.8	1.6	PC11-4_62-64-10-1	Euhedral spinel
230	0.660	0.422	0.390	6.8	15.4	16.8	37.7	7.2	8.5	4.5	1.0	2.2	PC11-4_62-64-10-2	Euhedral spinel
231	0.897	1.117	0.555	4.3	30.5	0.4	2.8	7.7	44.0	3.5	0.7	6.2	PC11-4_62-64-10-3	Euhedral spinel
232	0.902	1.104	0.550	4.7	31.9	0.2	1.0	8.1	43.3	3.2	0.6	7.0	PC11-4_62-64-10-4	Euhedral spinel
233	0.600	0.475	0.442	8.1	14.0	18.9	33.0	9.5	8.6	4.6	1.2	2.2	PC11-4_62-64-10-5	Euhedral spinel
234	0.813	0.980	0.546	7.6	33.2	0.9	3.9	10.2	33.6	4.2	1.1	5.3	PC11-4_62-64-10-6	Euhedral spinel
235	0.534	0.493	0.480	8.7	11.7	22.6	30.4	11.4	7.5	5.1	0.7	1.9	PC11-4_62-64-10-7	Euhedral spinel
236	0.556	0.558	0.501	8.4	11.8	20.4	28.7	12.7	9.5	5.1	0.5	2.7	PC11-4_62-64-10-8	Euhedral spinel
240	0.517	0.492	0.488	7.3	9.0	25.5	31.8	11.7	7.3	5.1	0.5	1.7	PC11-4_64-66-1-1	Euhedral spinel
241	0.543	0.565	0.510	7.4	10.4	21.6	30.5	12.5	9.8	5.5	0.5	1.8	PC11-4_64-66-1-2	Euhedral spinel
242	0.945	1.982	0.677	0.0	0.4	0.0	0.2	4.1	93.4	0.6	0.9	0.2	PC11-4_64-66-1-3	Dendritic spinel
243	0.844	1.180	0.583	4.3	23.6	1.3	7.1	9.7	44.1	4.3	0.8	4.8	PC11-4_64-66-1-4	Euhedral spinel

Table S5 (continued)

Analytical No.	Fe^{2+}	Fe^{3+}	Fe^{3+}/Fe_{tot}	Spinel (%)	Hercynite (%)	Picrochromite (%)	Chromite (%)	Magnesioferrite (%)	Magnetite (%)	Trevorite (%)	Jacobsite (%)	Ulvöspinel (%)	Comment and sample no.	Target mineral
244	0.996	1.902	0.656	0.0	1.8	0.0	0.2	1.8	92.0	0.6	0.7	2.8	PC11-4_64-66-1-5	Dendritic spinel
245	0.701	0.662	0.486	6.0	17.1	10.9	30.9	9.2	18.0	4.8	1.2	2.1	PC11-4_64-66-1-6	Euhedral spinel
250	0.590	0.578	0.495	6.9	11.9	18.5	31.9	11.3	11.4	5.5	0.7	1.9	PC11-4_64-66-4-1	Euhedral spinel
251	0.529	0.539	0.505	7.3	9.6	23.4	30.9	12.4	8.6	5.1	0.8	1.9	PC11-4_64-66-4-2	Euhedral spinel
252	0.660	0.638	0.491	6.8	15.7	13.1	30.3	10.3	15.6	5.4	0.6	2.2	PC11-4_64-66-4-3	Euhedral spinel
253	0.894	1.367	0.605	2.8	19.7	0.3	2.2	9.2	54.2	4.5	0.4	6.6	PC11-4_64-66-4-4	Euhedral spinel
254	0.745	0.779	0.511	5.3	16.6	8.8	27.6	10.1	24.8	3.5	0.6	2.8	PC11-4_64-66-4-5	Euhedral spinel
255	0.946	1.457	0.606	1.5	18.6	0.1	1.0	5.9	63.0	3.4	0.6	6.0	PC11-4_64-66-4-6	Euhedral spinel
256	0.749	0.942	0.557	4.7	17.3	6.0	21.9	10.8	29.7	6.0	0.7	3.0	PC11-4_64-66-4-7	Euhedral spinel
257	0.988	1.910	0.659	0.0	1.6	0.0	0.3	2.8	91.7	0.5	0.4	2.6	PC11-4_64-66-4-8	Dendritic spinel
258	0.565	0.446	0.441	7.0	9.8	24.7	34.5	10.0	8.6	2.8	0.8	1.8	PC11-4_64-66-5-1	Euhedral spinel
259	0.572	0.519	0.476	7.3	10.5	22.2	32.1	11.4	10.6	3.1	0.8	2.0	PC11-4_64-66-5-2	Euhedral spinel
260	0.849	0.824	0.493	4.7	23.5	4.3	21.3	7.7	30.2	2.5	0.7	4.9	PC11-4_64-66-5-3	Euhedral spinel
261	0.688	0.554	0.446	6.8	15.5	14.4	32.7	9.3	14.9	2.7	0.8	2.8	PC11-4_64-66-5-4	Euhedral spinel
262	0.976	1.172	0.546	2.4	29.1	0.2	2.3	5.1	51.3	1.6	0.5	7.4	PC11-4_64-66-5-5	Euhedral spinel
263	0.997	1.227	0.552	1.8	28.1	0.1	0.9	4.2	55.1	1.2	0.9	7.8	PC11-4_64-66-5-6	Euhedral spinel
264	0.607	0.478	0.440	7.7	13.4	19.4	33.6	9.5	9.7	4.0	0.7	2.0	PC11-4_64-66-5-7	Euhedral spinel
265	0.982	1.254	0.561	2.0	26.6	0.1	0.9	4.9	55.2	2.0	0.6	7.8	PC11-4_64-66-5-8	Euhedral spinel
266	0.636	0.433	0.405	7.0	13.9	18.6	37.1	7.9	9.0	3.9	0.8	1.8	PC11-4_64-66-5-9	Euhedral spinel
267	1.045	1.285	0.552	0.8	25.4	0.0	0.4	2.3	60.3	0.8	0.8	9.2	PC11-4_64-66-5-10	Euhedral spinel
268	0.603	0.420	0.411	7.4	12.9	20.8	36.1	8.3	7.7	4.2	0.8	1.8	PC11-4_64-66-5-11	Euhedral spinel
269	0.991	1.014	0.506	2.7	37.6	0.1	1.1	3.9	44.6	1.5	0.7	7.9	PC11-4_64-66-5-12	Euhedral spinel
270	1.022	1.143	0.528	1.5	31.9	0.0	0.7	3.0	52.2	1.1	0.8	8.7	PC11-4_64-66-5-13	Euhedral spinel
271	1.025	1.154	0.530	1.3	31.6	0.0	0.9	2.7	53.2	1.5	0.3	8.4	PC11-4_64-66-5-14	Euhedral spinel
272	0.688	0.526	0.433	6.8	15.8	14.6	33.9	8.7	14.0	3.1	0.6	2.6	PC11-4_64-66-5-15	Euhedral spinel
273	0.730	0.530	0.421	6.7	18.6	12.1	33.6	7.7	15.7	2.4	0.7	2.6	PC11-4_64-66-5-16	Euhedral spinel
274	0.994	1.198	0.547	2.1	26.5	0.2	2.7	5.0	53.0	1.7	0.3	8.6	PC11-4_64-66-5-17	Euhedral spinel
275	0.692	0.483	0.411	6.8	16.9	14.3	35.5	7.6	12.2	3.3	1.1	2.3	PC11-4_64-66-5-18	Euhedral spinel
276	0.954	1.063	0.527	2.9	29.1	0.7	7.3	5.5	45.5	1.2	1.0	6.8	PC11-4_64-66-5-19	Euhedral spinel
277	1.010	1.394	0.580	0.9	22.1	0.0	0.4	2.9	64.6	1.3	0.8	7.0	PC11-4_64-66-5-20	Euhedral spinel
292	0.593	1.601	0.730	1.5	9.8	0.1	0.4	11.8	32.7	34.8	0.8	8.2	PC11-4_64-66-5-35	Spherical spinel
293	0.797	1.542	0.659	1.6	10.8	0.0	0.3	11.0	48.1	17.2	0.7	10.3	PC11-4_64-66-5-36	Spherical spinel
294#	-0.188	1.806	1.116	0.4	4.2	0.0	0.3	7.6	-32.8	114.1	1.4	4.7	PC11-4_64-66-5-37	Spherical spinel

#Only this sample (Analytical No. 294) has a negative value of Fe^{2+} fraction and Fe^{3+}/Fe_{tot} exceeding 1 when assuming spinel stoichiometry.
